# QTL discovery for agronomic and quality traits in diploid potato clones using PotatoMASH amplicon sequencing

**DOI:** 10.1093/g3journal/jkae164

**Published:** 2024-07-19

**Authors:** Lea Vexler, Maria de la O Leyva-Perez, Agnieszka Konkolewska, Corentin R Clot, Stephen Byrne, Denis Griffin, Tom Ruttink, Ronald C B Hutten, Christel Engelen, Richard G F Visser, Vanessa Prigge, Silke Wagener, Gisele Lairy-Joly, Jan-David Driesprong, Ea Høegh Riis Sundmark, A Nico O Rookmaker, Herman J van Eck, Dan Milbourne

**Affiliations:** Teagasc, Crop Science Department, Oak Park, Carlow R93 XE12, Ireland; Plant Breeding, Wageningen University & Research, P.O. Box 386, Wageningen 6700 AJ, The Netherlands; The Graduate School Experimental Plant Sciences, Droevendaalsesteeg 1, 6708 PB Wageningen, The Netherlands; Teagasc, Crop Science Department, Oak Park, Carlow R93 XE12, Ireland; Teagasc, Crop Science Department, Oak Park, Carlow R93 XE12, Ireland; Plant Breeding, Wageningen University & Research, P.O. Box 386, Wageningen 6700 AJ, The Netherlands; The Graduate School Experimental Plant Sciences, Droevendaalsesteeg 1, 6708 PB Wageningen, The Netherlands; Teagasc, Crop Science Department, Oak Park, Carlow R93 XE12, Ireland; Teagasc, Crop Science Department, Oak Park, Carlow R93 XE12, Ireland; Flanders Research Institute for Agriculture, Fisheries and Food (ILVO), Plant Sciences Unit, Caritasstraat 39, Melle 9090, Belgium; Department of Plant Biotechnology and Bioinformatics, Faculty of Sciences, Ghent University, Technologiepark 71, Ghent 9052, Belgium; Plant Breeding, Wageningen University & Research, P.O. Box 386, Wageningen 6700 AJ, The Netherlands; Plant Breeding, Wageningen University & Research, P.O. Box 386, Wageningen 6700 AJ, The Netherlands; Plant Breeding, Wageningen University & Research, P.O. Box 386, Wageningen 6700 AJ, The Netherlands; SaKa Pflanzenzucht GmbH & Co. KG, Eichenallee 9, Windeby 24340, Germany; SaKa Pflanzenzucht GmbH & Co. KG, Eichenallee 9, Windeby 24340, Germany; Germicopa Breeding, 1 Allée Loeiz, Quimper 29000, France; Meijer Potato, Bathseweg 47, Rilland 4411 RK, The Netherlands; Danespo A/S, Dyrskuevej 15, Give DK-7323, Denmark; AVERIS Seeds, Valtherblokken zuid 40, Valthermond 7876 TC, The Netherlands; Plant Breeding, Wageningen University & Research, P.O. Box 386, Wageningen 6700 AJ, The Netherlands; The Graduate School Experimental Plant Sciences, Droevendaalsesteeg 1, 6708 PB Wageningen, The Netherlands; Teagasc, Crop Science Department, Oak Park, Carlow R93 XE12, Ireland

**Keywords:** diploid potato, diploid breeding, haplotypes, SNPs, GWAS, QTL, PotatoMASH, Plant Genetics and Genomics

## Abstract

We genotyped a population of 618 diploid potato clones derived from six independent potato-breeding programmes from NW-Europe. The diploids were phenotyped for 23 traits, using standardized protocols and common check varieties, enabling us to derive whole population estimators for most traits. We subsequently performed a genome-wide association study (GWAS) to identify quantitative trait loci (QTL) for all traits with SNPs and short-read haplotypes derived from read-backed phasing. In this study, we used a marker platform called PotatoMASH (Potato Multi-Allele Scanning Haplotags); a pooled multiplex amplicon sequencing based approach. Through this method, neighboring SNPs within an amplicon can be combined to generate multiallelic short-read haplotypes (haplotags) that capture recombination history between the constituent SNPs and reflect the allelic diversity of a given locus in a different way than single bi-allelic SNPs. We found a total of 37 unique QTL across both marker types. A core of 10 QTL was detected with SNPs as well as with haplotags. Haplotags allowed to detect an additional 14 QTL not found based on the SNP set. Conversely, the bi-allelic SNP set also found 13 QTL not detectable using the haplotag set. We conclude that both marker types should routinely be used in parallel to maximize the QTL detection power. We report 19 novel QTL for nine traits: Skin Smoothness, Sprout Dormancy, Total Tuber Number, Tuber Length, Yield, Chipping Color, After-cooking Blackening, Cooking Type, and Eye depth.

## Graphical Abstract

**Graphical Abstract:**
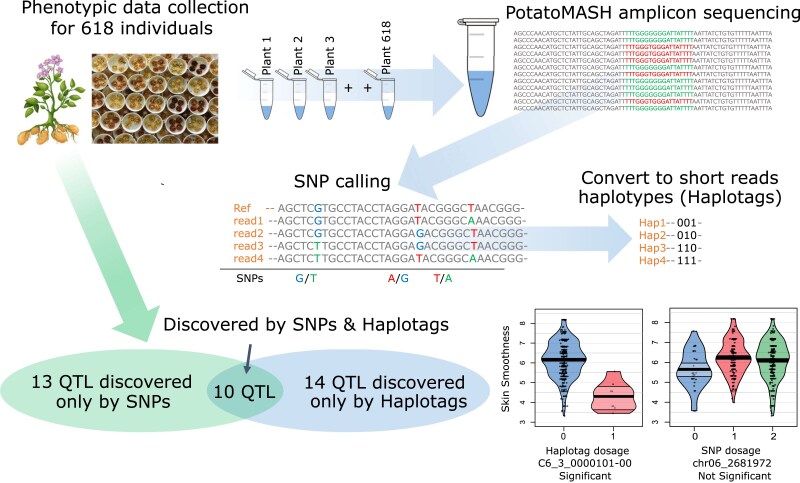

## Introduction

Potato is an important food crop and is a key element in the global food security, as well as being a valuable cash crop (FAO Crops statistics database: http://faostat.fao.org/). Given the importance of potato, and the potential impact of factors such as climate change and world population increase, the ability to rapidly and precisely breed potato varieties combining large numbers of favorable traits has been widely recognized. Outbreeding and tetraploidy of modern cultivated potato are complicating factors to achieve greater genetic gains in potato breeding. The potential to rapidly harness recurrent selection to fix favorable alleles and purge deleterious ones across cycles of selection is limited. In response, several groups have started programmes to increase the effectiveness of recurrent selection by breeding at the diploid level (Lindhout *et al*. 2011; Zhang *et al*. 2021; Bradshaw 2022; Song and Endelman 2023) through utilization of self-compatible diploids. Selfing allows fixation of alleles linked to important traits, after which inbreeding depression is addressed by crossing divergent, high performing inbred lines, producing uniform F1 progeny exhibiting hybrid vigor (Lindhout *et al*. 2011; Jansky *et al*. 2016; Zhang *et al*. 2021). Diploid potato has a genetically encoded gametophytic self-incompatibility system (Kao and McCubbin 1996; Hosaka and Hanneman 1998). The ability to self-fertilize and backcross lines efficiently is mediated by the *Sli* locus, originally described by Hosaka and Hanneman (1998), and more recently mapped by Clot *et al.* (2020). The latter study found that *Sli* is not only available in clones derived from *Solanum chacoense* but also in material derived from the early variety Rough Purple Chili. Hence, the *Sli* gene is widely present in tetraploid varieties and diploid material derived from these varieties.

The “precision breeding” approach exemplified by utilizing self-compatibility to accumulate and fix traits in potato requires tools to manage the genetic diversity at important loci like *Sli* into diploid breeding material. As well as characterizing the genetic location and origin of *Sli*, Clot *et al*. (2020) developed and validated diagnostic KASP markers to enable efficient marker assisted selection (MAS) for the *Sli* locus. Other traits important to this breeding approach, are those related to sexual polyploidizations (Clot *et al*. 2024), as well as tolerance to inbreeding depression (van Lieshout *et al*. 2020; Zhang, Yin, *et al*. 2022). These resources will facilitate the reproductive aspects of breeding potato at the diploid level, enabling MAS strategies to manage the introgression of key alleles to facilitate the process. In addition to this, it would be useful to develop a resource for genome-based breeding methods to support the improvement of other important traits in potato at the diploid level such as disease resistance, and agronomic and quality traits to develop specific ideotypes to serve different market segments (e.g. fresh consumption, processing, starch). Identifying marker-trait associations is essential to drive MAS for rapid breeding. One powerful strategy to discover markers linked to complex traits is to perform genome-wide association studies (GWAS). In the last decades, numerous association studies have been conducted on potatoes, mostly at the tetraploid level, reflecting the above desire to characterize important traits directly in breeding-relevant material (Malosetti *et al*. 2007; D’hoop *et al*. 2008; Li *et al*. 2010; Baldwin *et al*. 2011; Lindhout *et al*. 2011; Urbany *et al*. 2011; D’hoop *et al*. 2014; Rosyara *et al*. 2016; Schönhals *et al*. 2017; Sharma *et al*. 2018; Klaassen *et al*. 2019; Byrne *et al*. 2020; Prodhomme *et al*. 2020; Vos *et al*. 2022; Zhang, Qu, *et al*. 2022). Genetic studies in diploid potato have largely been based on mapping specific traits using biparental crosses, with relatively few, and generally smaller scale GWAS studies in diploid germplasm sets (Díaz *et al*. 2021; Parra-Galindo *et al*. 2021; Yang *et al*. 2021), and, to our knowledge, this study is the most extensive diploid potato panel, multitrait GWAS so far.

In potato, genotyping-by-sequencing (GBS) as marker discovery and screening strategy usually yields tens to hundreds of thousands of SNPs: e.g. 186k (Sverrisdóttir *et al*. 2017), 40k (Byrne *et al*. 2020), 22.5k markers (Wang *et al*. 2021); and SNP arrays in potato typically contain up to tens of thousands of markers: e.g. 20k (Vos *et al*. 2022) and 8.3k markers (Mosquera *et al*. 2016; Rosyara *et al*. 2016). In a previous study, we developed a marker system called PotatoMASH (Leyva-Pérez *et al*. 2022), with the specific ambition of exploring the potential of low cost, genome-wide genotyping for application in potato breeding and genetics. PotatoMASH surveys 339 loci using a multiplex amplicon sequencing approach followed by deep NGS sequencing (2 × 150 bp Illumina sequencing). The question of what is the minimum number of loci that would provide reasonable genome coverage for effective downstream analysis such as GWAS is in the basis of the development of PotatoMASH. It was previously found that “useful” levels of linkage disequilibrium (LD) extended between 0.6 and 1.5 Mb depending on the population under examination and the LD criterion used. In addition, almost no LD decay was observed across the pericentromeric heterochromatin (Vos *et al*. 2015; Sharma *et al*. 2018). This is why PotatoMASH was designed to detect variation at 339 loci evenly distributed every 1 Mb across the euchromatic portion of the genome (Leyva-Pérez *et al*. 2022), so no site could be more than 0.5 Mb from at least one locus. On the other hand, SNPs are almost entirely bi-allelic, and surveying a single SNP locus per megabase will not efficiently survey the diversity of real haplotypes at any one locus. Because of the high SNP density in potato germplasm, PotatoMASH actually yields >2,000 SNPs, and additional tools can be used for read-backed phasing (Schaumont *et al*. 2022), to create short haplotypes (165–180 bp) that can be used as a multiallelic marker system. These multiallelic haplotags better represent the real allelic composition at a locus and may have better discriminatory power than SNPs for quantitative trait loci (QTL) detection in genome-wide association analysis. Proof of concept of the detection power of PotatMASH was provided by detecting the same QTL associated with fry color that was originally detected in a GWAS involving 40 K GBS-derived SNP markers (Byrne *et al*. 2020). In addition, we observed that the multiallelic haplotags potentially had better discriminatory power than SNPs in GWAS, since the QTL was only detected when using multiallelic haplotags and not SNPs (Leyva-Pérez *et al*. 2022).

In this study, we describe a set of 618 diploid potato genotypes, assembled by a consortium of six breeding programmes (DIFFUGAT project https://diffugat.eu/). This material will form the basis of the diploid breeding approaches described above. Phenotypic data were collected on 23 traits over 3 years (2019–2021) This collaborative project aims to improve commercially relevant traits in a diploid genetic background with several essential reproductive traits such as (1) self-compatibility to allow fixation of genetic gains, (2) 2n gametes to allow sexual polyploidization and hybridization with varieties, and (3) a high level of male and female fertility.

The objectives of this study were (1) to characterize this panel for a set of traits that are routinely phenotyped during the selection process in these breeding programmes; (2) to map loci underlying the control of these traits using GWAS; (3) to test haplotags based QTL detection in a wide variety of traits. A longer-term goal is to utilize this information to develop marker-based tools to facilitate selection in this germplasm and extended sets of breeding clones related to it within individual programmes.

## Materials and methods

### Plant materials and phenotypic evaluation

We used a panel of 618 diploid potato clones provided by a consortium composed of commercial breeders and research institutes. The panel represents clones from diploid breeding programs, where commercially relevant traits are combined with traits important for diploid breeding, such as fertility, self-compatibility, and 2n gamete production. Contributions were made by C. Meijer B.V., The Netherlands—225 individuals; Wageningen University, The Netherlands—134; Danespo A/S, Denmark—101; SaKa Pflanzenzucht GmbH & Co. KG, Germany—93; Germicopa Breeding, France—60; and Averis Seeds B.V., The Netherlands—17 individuals. Because of the commercial nature of the material, pedigree information could not always be provided. In general, the panel is composed of elite diploid breeding clones, primary dihaploids extracted from tetraploid varieties and donors of resistance and fertility traits.

For intellectual property reasons, the breeding material was not shared between companies. Instead, consortium members evaluated their own material using an augmented design, with replicated checks shared across the 6 locations over 3 years (2019–2021). Each company implemented field trial design according to their own system, but the check varieties were included across programmes: Two control varieties were used in 2019 (Lady Claire and Fontane), and 2 additional control varieties were introduced to the experiments in years 2020 and 2021 (Darling and Laperla). Those 4 controls were used to estimate the environmental variance across the sites. Some additional controls were introduced locally within each company in accordance with their local protocols for field trials. The size of each experimental unit was 8 plants per plot, except for Averis, who planted 14 plants per plot, and we accounted for this in measurements that are influenced by number of plants: Yield, Total Tuber Number and Dry Matter Content, by rescaling the measurement proportionally to 8 plants. All companies used a planting distance of 75 cm between the ridges and 30 to 35 cm between plants. Fungicide treatment, fertilizer, and irrigation were applied according to each company's own growing protocol and according to needs each season. More experimental information is provided in Supplementary File 1.

An overview of all 23 morphological, agronomic, and quality traits examined in this study is shown in Table 1. All consortium members used an agreed standardized set of protocols for scoring each trait. While most phenotyping efforts need no further clarification, some observation methods are briefly outlined below. Tuber length (TPM) was measured by counting how many randomly picked tubers are required to fill a PVC gutter of 1 m length (Supplementary File 2). This means that higher scores are given to shorter tubers. Enzymatic Browning (EnzB) was scored on strings of tuber tissue 2 h after being scraped from peeled raw potatoes using a coarse kitchen grater. Presentability of tubers (PTY) is a holistic trait as defined by breeders” experience and includes regularity and goodness of shape, size, eyes, and skin phenotypes. Skin Smoothness (SkinS) relates to the feel and washability of tubers. Skin Brightness (Gloss) is a visual assessment referring to a glossy or shiny skin finish. Cooking Type (CT) was evaluated by boiling samples of two tubers per plot for 25 min. After-cooking blackening (ACB) was assessed on the cooled-down potato 1 day after cooking. Processing quality was assessed using Chipping Color data from three treatments: (1) tubers stored at 8°C for 4 months before crisping (QDC1-8), tubers stored at 8°C for 6 months before crisping (QDC2-8) and tubers stored at 4°C for 6 months before crisping (QDC2-4). The color is assessed for three potato tubers, cut into slices of 1 mm and fried at 180°C until water (“bubbles”) has disappeared from the crisps.

**Table 1.** Overview of phenotypic traits, scales and numbers of genotypes (including controls) that were assessed for each trait and number of observations over three years.

Trait	Abbr.	Scale	Number of genotypes (including controls)	Number of observations over 3 yr
Yield	YLD	In kg per plant, fresh weight at harvest	567	1650
Canopy stage 1 6 wk after planting	Can1	1 = plants have not yet emerged to 9 = largest canopy in the trial	475	1347
Canopy stage 2 10 wk after planting	Can2	1 = plants have not yet emerged to 9 = largest canopy in the trial	550	1295
Tuber Length	TPM	Tubers per meter count was used with correction table	307	907
Total Tuber Number	TTN	Count of tubers	523	1452
Tuber Shape	TSH	1 = very round, 2 = round, 3 = round-oval, 4 = round-oval to oval, 5 = oval, 6 = oval to long-oval, 7 = long-oval, 8 = long, 9 = very long	569	1646
Yellow Skin Color	YSC	1 = white, 2 = cream, 3 = light yellow, 4 = yellow, 5 = dark yellow, 6 = brown	536	1542
Yellow Flesh Color	FC	1 = clear white, 2 = white, 3 = cream, 4 = light yellow, 5 = yellow, 6 = dark yellow, 7 = very dark yellow	549	1575
Eye Depth	EYE	1 = very deep to 9 = very shallow	567	1648
Presentability of Tubers	PTY	1 = very bad to 9 = very good	565	1644
Skin Smoothness	SkinS	1 = rough to 9 = very smooth	567	1488
Skin Brightness	Gloss	1 = dull to 9 = clear	554	1411
Sensitivity to Common Scab	Scab	1 = heavy symptoms to 9 = no symptoms	424	1278
Enzymatic Browning	EnzB	1 = ink black, 2 = uniformly black, 3 = discoloration to black, 4 = darkening of red and gray discoloration, 5 = bright red and dark gray discoloration, 6 = start of red/gray discoloration, 7 = clear start of discoloration, 8 = very slight discoloration, 9 = no discoloration	552	1467
Cooking Type	CT	2 = very floury, loose boiling, sloughing, 4 = floury, crumbly and fairly loose, 6 = slightly floury and fairly firm, 8 = not floury, firm cooking, 9 = extreme firmness	555	1393
After-cooking blackening	ACB	1 = very dark to 9 = pure color (no darkening at all)	558	1498
Chipping Color 1st time point stored at 8°C	QDC_1–8	1 = very dark to 9 = pure color (no darkening at all)	559	1593
Chipping Color 2nd time point stored at 8°C	QDC_2-8	1 = very dark to 9 = pure color (no darkening at all)	367	857
Chipping Color 2nd time point stored at 4°C	QDC_2-4	1 = very dark to 9 = pure color (no darkening at all)	505	1353
Dry Matter Content	DM	% relative to fresh weight	566	1626
Sprout Dormancy	SD	1 = heavy sprouting (early) to 9 = no sprouting	536	1416
Tuber Regularity	REG	1 = bad to 9 = good	565	1646
Maturity	MAT	1 = plants still green and flowering to 9 = plants reached senescence	427	857

### Statistical analysis of phenotypic data

**Fig. 1.**
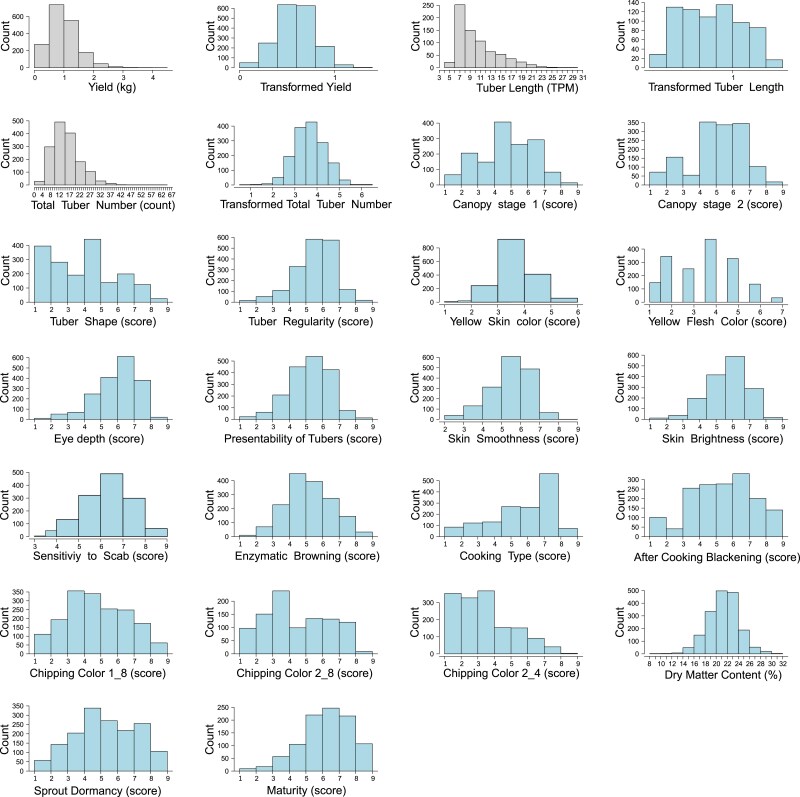
Frequency distribution histograms for all traits of this study, across all companies. The horizontal axis indicates the data range of traits, and the vertical axis indicates the frequency of individuals. For traits Yield, Tuber Length and Total Tuber Number per plant: the histograms for Yield, Tuber Length and Total Tuber Number (in gray) represent the raw data, and the blue histograms represent the transformed data that we used in the downstream analysis.

All statistical analyses and data visualizations were performed using R version 4.2.1 unless otherwise specified in results. Visual inspection of the distribution of the data and quantile–quantile (QQ) plots of residuals vs quantiles revealed some obvious deviations from homoscedasticity or normality in the continuous traits: Yield, Tuber Length, and Total tuber number per plant (Fig. 1). The data of those traits were transformed with Yeo-Johnson transformation using the R package “car” (Fox and Weisberg 2019). Although the majority of the traits were measured on an ordinal scale, an inspection of diagnostic plots for residuals indicated no strong violations of the assumption of normal error distributions (Supplementary File 3) and were all analyzed as quantitative traits, as previously performed (D’hoop *et al*. 2008), assuming the error variation to be normally distributed with constant variance.

Check varieties were used in the estimates of the Best Linear Unbiased Estimators (BLUEs) of phenotypic means of all 23 traits across years and locations but were excluded from the GWAS. We used a multiple linear regression package lme4, using the lm function (Bates *et al*. 2015) to calculate the BLUEs with the following equation:


Trait=Genotype+Year+Location+Location*Year+Error,

where genotype is the clone name and does not include any genetic information such as pedigree due to intellectual property rights and Location is the site of each company. Location*Year effect was applied when analyzing data from more than one company. All independent variables: Genotype, Year, Location and Location*Year, were considered as fixed effects due to the low number of levels.

Least square means, calculated for the BLUEs with the R package “emmeans” (Lenth *et al*. 2021), served as the final phenotypic data used in the association analysis.

A Pearson’s correlation matrix between the vegetation indices and the vegetative growth parameters was generated using the package corrplot for R. Correlation coefficients were tested at *P* = 0.05.

Broad-sense heritabilities (*H*^2^) were calculated for each breeding population separately on an entry-mean basis according to the formula:


$$H^{2}=\sigma_{g}^{2}/(\sigma_{g}^{2}+\sigma_{\mathrm{gy}}^{2}/n_{\mathrm{year}}+\sigma_{e}^{2}/n_{\mathrm{year}}),$$

where σ_g_^2^ is the genotypic variance, σ_gy_^2^ is the genotype-by-year variance, σ_e_^2^ the error variance, and *n*_year_ is the number of years.

The k-matrix of the genomic data was calculated with the R package GWASpoly (Rosyara *et al*. 2016) and the modeled least square means were used to calculate the Marker-based heritabilities, (h^2^_SNPs_ and h^2^_haplotags_) with the R package “heritability” with the *marker_h2_means* function (Kruijer *et al*. 2014; Kruijer and White 2023).

### Genotypic data

#### Data collection with PotatoMASH

Leaf material was sampled in 2019, the first year of the field trials, freeze-dried and stored with silica gel until use. Approximately 5 mg of dry tissue was used to extract DNA with Mag-BIND Plant DNA DS Kit (Omega-VWR M1130-00, Philadelphia, USA), using the KingFisher Flex automated extraction & purification system (Thermo Scientific, Austin, TX, USA).

PotatoMASH libraries were obtained and haplotyping was performed as in Leyva-Pérez *et al*. (2022) (https://www.protocols.io/view/potatomash-library-construction-e6nvw53zdvmk/v2) with the following adjustments to the bioinformatics pipeline: Merged and filtered reads were mapped to the *S. tuberosum* genome v6.1 (Pham *et al*. 2020). Variant calling was then filtered with a minimum allele frequency of 0.01 and a maximum of 0.99: *vcftools –bcf PotatoMASH.bcf –out PotatoMASH –min-alleles 2 –max-alleles 2 –recode –recode-INFO-all –minQ 30 –minDP 6 –maf 0.01 –max-maf 0.99 –remove-filtered-all –max-missing 0.5*.

Haplotypes nomenclature is given by the software SMAP (Schaumont *et al*. 2022) as a binary string code for the set of SNPs called in a specific locus, where the reference allele of each SNP is coded as “0” and the alternative allele is coded as “1” in a specific haplotype (Fig. 4c). The final haplotag name is the PotatoMASH locus name plus the binary string in which 0 means same base as reference genome, 1 is alternative base, and “-” is an indel at that SNP position (e.g. C1_1_000110-10).

For the 334 polymorphic loci, the average locus correctness score (number of samples with sum of discrete haplotag dosage calls equals 2, divided by total number of samples with sufficient read depth for that locus, expressed as percentage) was 92. SMAP also calculates the sample correctness score per sample (number of loci where the sum of discrete haplotag dosage calls equals 2, divided by the total number of loci with sufficient read depth, expressed as percentage). Since the average locus correctness score was high for the 334 loci, we assumed that individuals with low sample correctness score would be due to technical errors or putative cross contamination. Therefore, we removed 21 genotypes with a sample correctness lower than 40. A final panel of 558 genotyped individuals was used for the GWAS. SNPs and haplotags datasets are provided in Supplementary Files 4 and 5.

#### Population structure

Population structure was evaluated using a principal component analysis (PCA) calculated with Plink 1.9 using SNPs with a minimum allele frequency >0.01 (Purcell *et al*. 2007). The population genetic structure was assessed using the Bayesian clustering method implemented in STRUCTURE version 2.3.4 (Pritchard *et al*. 2000). An admixture model and correlated allele frequencies were chosen for estimating the proportion of ancestral contribution in each accession. We tested various K-values ranging from 1 to 10 with 3 independent replications at each K, 10,000 generations burn-in period and 10,000 Markov Chain Monte Carlo (MCMC) repetitions. Calculation of Delta K: (1) Mean L(K) (±SD) was done over 3 independent runs for each K value. (2) Rate of change of the likelihood distribution (mean ± SD) was calculated as L′(K) = L(K) − L(K − 1). 3. Absolute values of the second order rate of change of the likelihood distribution (mean ± SD) were calculated according to the formula: |L′′(K)| = |L′(K + 1) − L′(K)|. 4. ΔK calculated as ΔK = mean|L′′(K)|/sd[L(K)] (Evanno *et al*. 2005). Visualizing admixture plot was done with the fastSTRUCTURE software distruct.py function (Raj *et al*. 2014).

#### Genome-wide association studies

Two datasets were used for the GWAS. We first identified SNPs across the sequenced amplicons and used these as a data set in a GWAS. We then used this SNP set to construct short haplotypes with SMAP *haplotype-sites* tool (see “Data collection with PotatoMASH” section) combined with discrete genotype calling, that were then used simply as presence-absence markers for GWAS. The distinct haplotags were treated as “pseudoSNPs” for the purpose of the analysis.

Association analysis for both SNP and haplotag data was done with the R package GWASpoly (Rosyara *et al*. 2016). Population structure was controlled using the K model and QQ plots were used to assess if there was sufficient control of population structure. The function GWASpoly with additive and nonadditive models was used to test for association at each marker. Marker curation was carried out using the maximum genotype frequency option with default parameter setting (geno.freq = 1–5/*N*, where *N* is the number of genotypes), so markers present in fewer than 5 individuals are removed. The genome-wide false discovery rate was controlled using the M.eff method (a Bonferroni-type correction but using an effective number of markers that accounts for linkage disequilibrium (LD) between markers) at level = 0.05. We did not use the leave-one-chromosome-out (LOCO) approach due to the inflation of the *P*-values as observed with the QQ plots.

## Results

### Phenotypic data

Taken together, phenotyping of the panel of 618 diploids resulted in 32,590 data points, collected over 3 years, across 6 locations, for a total of 23 agro-morphological and quality traits (Fig. 1, Table 2). These data were unbalanced given that some locations/breeders focused on a single niche market (e.g. starch). A strong year-by-location interaction was observed (Supplementary File 6) using control varieties planted across all sites. From these raw data best linear unbiased estimators (BLUEs) were calculated while taking the year-by-location interaction into account with the regression models.

**Table 2.** Mean, standard deviation (SD), minimum (Min), maximum (Max) values, and Broad sense (*H*^2^) Heritability (%) for all traits.

Trait	Min	Max	Mean	SD	Average *H*^2^ across all companies	Number of companies tested
Canopy stage 1	1	9	5.24	1.69	60.1	5
Canopy stage 2	1	9	5.53	1.73	75.2	6
Yield	0.003	4.26	0.98	0.5	85.5	6
Tuber Length	5.37	28.11	10.37	4.01	87.1	4
Total Tuber Number	0.75	61.25	15.42	6.72	69.2	5
Tuber Shape	1	9	4.4	2.07	90.4	6
Tuber Regularity	1	9	6.05	1.23	64.9	6
Yellow Skin Color	1	6	4.12	0.8	63.6	6
Yellow Flesh Color	1	7	3.6	1.5	88.8	6
Eye depth	1	9	6.47	1.31	82.6	6
Presentability of Tubers	1	9	5.65	1.24	74.5	6
Skin Smoothness	2	9	5.95	1.09	60.3	6
Skin Brightness	1	8	5.58	1.09	62.1	5
Sensitivity to Common Scab	3	9	6.76	1.16	50.6	6
Enzymatic Browning	1.5	9	5.51	1.36	81.7	6
Cooking Type	2	9	6.62	1.68	74.3	6
After-cooking Blackening	1	9	5.9	1.9	72.7	6
Chipping Color 1_8	1	9	5.25	1.86	77.8	6
Chipping Color 2_8	1	9	4.93	1.96	83.6	3
Chipping Color 2_4	1	9	3.82	1.77	81.8	4
Dry Matter Content	8.35	31.08	21.35	2.84	88.7	6
Sprout Dormancy	1	9	5.81	1.82	74.9	5
Maturity	1	9	6.6	1.44	82.0	4

We could not calculate the broad sense heritability (*H*^2^) across all companies, as only the control varieties were shared. The heritabilities presented in Table 2 are the average of the estimated trait heritability for each company and varied mostly between moderate to high values, ranging from 50 to 90%. Traits largely controlled by single loci such as Tuber shape (TSH), Yellow flesh color (FC), and Maturity (MAT), typically show *H*^2^ between 82 and 90%, according to expectations. Some of the complex polygenic traits like Dry Matter Content (DM) and Yield, also show an exceptionally high *H^2^* of 85–89%. Furthermore, the majority of the processing and quality traits such as Enzymatic Browning (EnzB), Cooking Type (CT), After-cooking Blackening (ACB), and Chipping Color showed moderately high *H*^2^ values (73–84%). Marker-based heritability, were also calculated using both marker types, SNPs and haplotags, and were lower than the broad sense heritability for all traits (Supplementary File 7).

### Correlations between traits

**Fig. 2.**
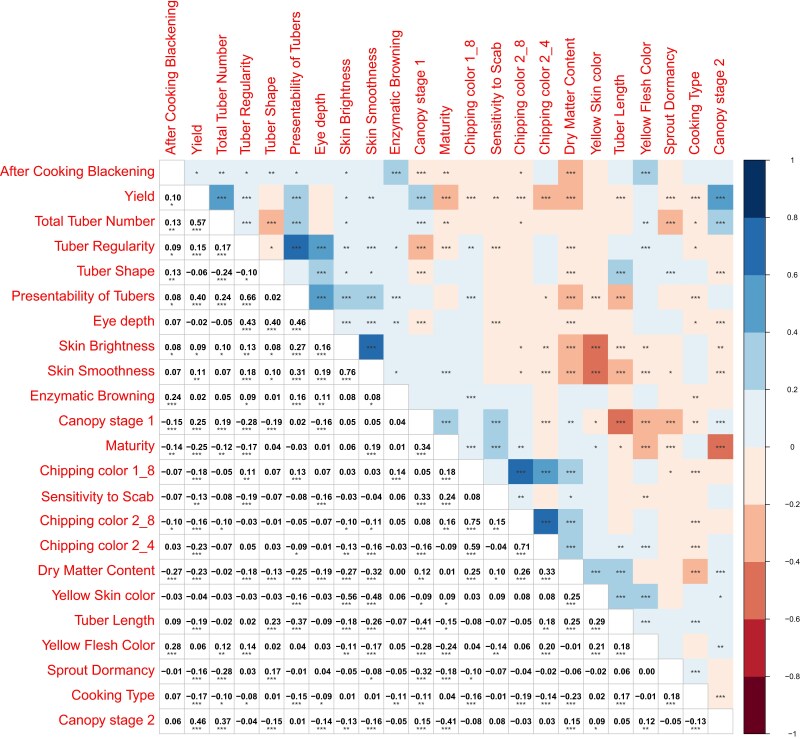
Matrix of pairwise Pearson's correlation between all traits. Positive correlations are displayed in blue and negative correlations in red. Color intensity is proportional to the correlation coefficients according to the scale displayed on the right. Marking of significance level: ***0.001, **0.01, *0.05.

The correlations between traits are shown in Fig. 2, and are based on the phenotype estimated means (Supplementary File 8). The highest positive correlations were observed between Skin Smoothness and Skin Brightness (while both had a negative correlation with Yellow Skin Color). High correlations were also observed for tuber visibility traits such as Tuber Regularity, Tuber Presentability, and Eye depth. Yield showed a negative correlation with Maturity and a positive correlation with Canopy development. Canopy stage 1 and Canopy stage 2 positively correlated with Total Tuber Number.

### Genotyping, variant calling, and genetic diversity

**Fig. 3.**
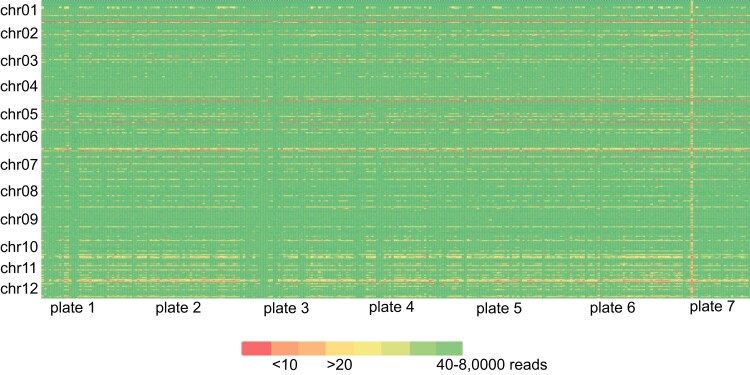
Coverage of the 339 PotatoMASH core loci. Heat map of the number of merged and filtered reads of 618 samples (in columns, each plate is of ∼96 samples) that mapped to each locus (in rows).

After merging and filtering reads, we retained on average 275,855 reads per sample, which corresponds to an average of 813 reads per amplicon per sample, well exceeding the required minimum of 20× read depth recommended for SMAP haplotype calling for diploids. The amplification efficiency of the primer pairs (either low or high) was consistent across most samples (Fig. 3).

**Fig. 4.**
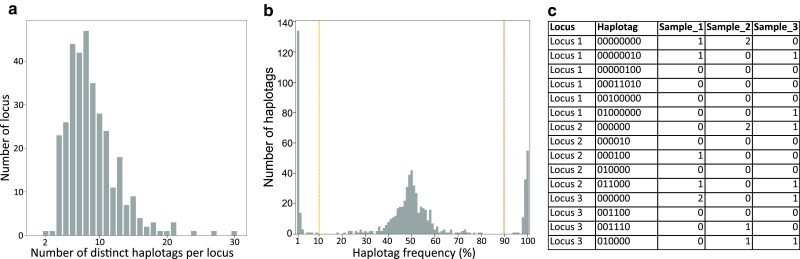
a) Haplotag diversity distribution of 334 loci across the individuals in the panel. b) Haplotag frequency spectrum of one individual, the haplotag frequency is calculated by the relative read depth (%) for each haplotag within its locus. c) Example of tabular data generated by SMAP haplotype-sites with 3 genotypes (samples), 3 loci, 15 haplotags, and diploid discrete dosage calls for each locus/sample. Loci 1 and 3 include haplotags not detected in samples 1–3 but in other genotypes not shown (samples 4-558).

After filtering, 2,730 SNPs were identified across the panel (Supplementary File 4). Out of 339 PotatoMASH target loci, SMAP *haplotype-sites* could identify 334 loci with polymorphic, multiallelic haplotypes. A total of 2,955 short multiallelic haplotags were identified across the panel (Supplementary File 5), ranging from 2–30 haplotags per locus, while most loci had 8–9 haplotags per locus (Table 3, Fig. 4a). This is higher than previously reported by Leyva-Pérez *et al*. (2022) in a tetraploid population where 2–14 haplotags per locus (on average 6 haplotags per locus) were reported. The higher haplotype diversity suggests higher genetic diversity in the used diploid panel.

**Table 3.** Summary of genotyping and variant calling with PotatoMASH.

Total samples	558
SNPs called	7503
SNPs filtered	2730
Polymorphic loci	334
Number of haplotypes	2955
Haplotypes per locus	2–30 (8.8 avg.)
2 haplotags called per locus per individual (either homozygous or heterozygous)	91%

As expected in our diploid panel, 2 haplotags (either homozygous or heterozygous) were successfully called at each locus, for each individual, in 91% of cases. SMAP analyses the relative read depth per haplotag per locus per individual, and outputs the distribution across all loci to check, if that fits the typical frequency spectrum expected for diploids (Fig. 4b). Thirty-nine of the individuals showed a tetraploid typical frequency spectrum and were excluded. As final output, we obtained a table with discrete dosage calls for each haplotag per locus, per sample (Fig. 4c), which was used for downstream analysis.

### Population structure

**Fig. 5.**
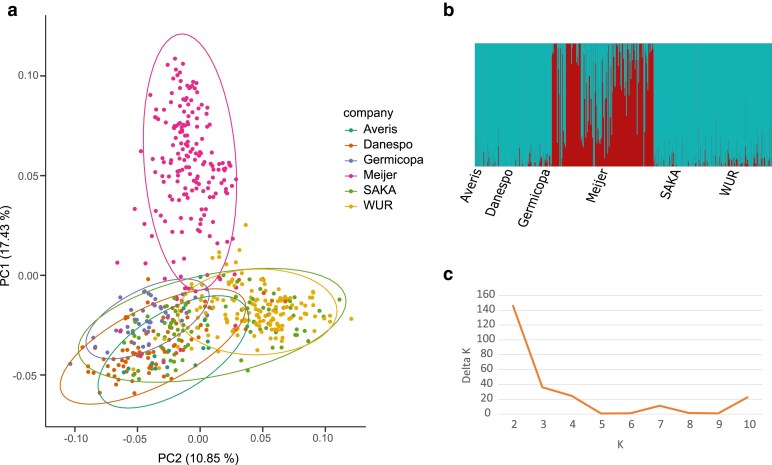
a) Principle component analysis (PCA) with SNP data of all six companies. b) Estimation of hypothetical sub-populations using K-values. c) The number of identified sub-populations (K) vs DeltaK estimated based on Evanno method.

We examined population structure by principal component analysis (PCA) using the SNP data and observed 2 main clusters, with separation mainly occurring on the 1st principal component which explains ∼17% of the genetic variation, indicating that the diploid population of Meijer deviates from the gene pools of the other breeding programs (Fig. 5a). We also examined the underlying population structure of the panel through Bayesian-based approach using STRUCTURE v 2.3.4. and with the log mean probability and deltaK (change in log probability) per K (number of sub-populations) generated the highest peak at K = 2 (Fig. 5c), and this confirmed the conclusion of two sub-populations. We therefore decided to perform QTL discovery using three sets of potato genotypes: the “full panel”, the sub-populations “only Meijer” and the rest not including Meijer (referenced as “no Meijer”). In this way, we were hoping to capture QTL that were robust across all subpopulations in addition to subpopulation-specific QTL.

### GWAS of multiple traits

To capture all the potential QTL, we performed six GWAS (three genotype-sets described above with the two marker-sets, SNPs and haplotags).

**Fig. 6.**
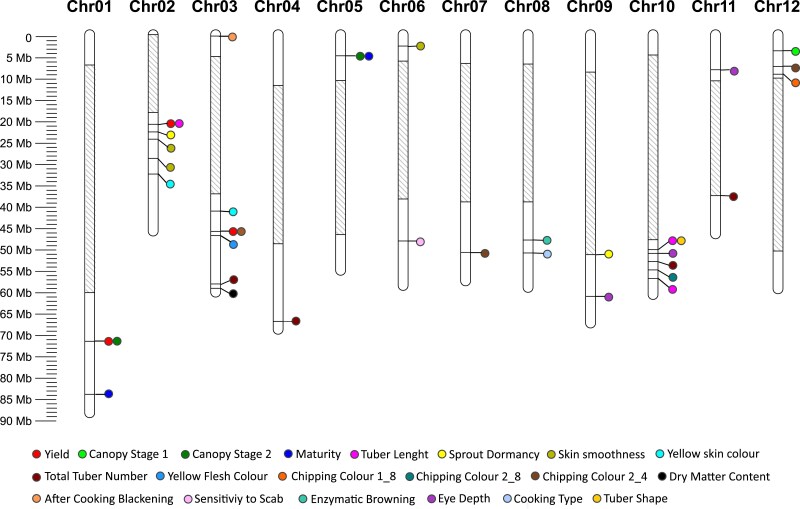
Physical map with the positions (Mb) of all QTL. Gray regions on the chromosome indicate pericentromeric heterochromatin, without PotatoMASH amplicons.

We identified 37 QTL for 20 out of 23 traits. For three traits: Tuber regularity, Skin brightness and Presentability of tubers we did not detect QTL. Of the 37 QTL identified, only 10 QTL were detected with both SNPs and haplotags. Fourteen QTL were only detected by haplotags, and 13 QTLs were only detected by SNPs (Fig. 6; Table 4). The full information of all the significant markers, including their marker's effects, are provided in Supplementary File 9.

**Table 4.** Overview of the 37 QTLs for the 20 trait, with the significant markers for each population: underlined—QTL only with SNPs, italics—QTL only with haplotags, bold—QTL with both. Columns from left to right; Trait, given name of QTL, QTL location in potato genome DMv6, name of haplotag and SNP for each population, previously reported QTL and positions in Mb when available, in potato genome DMv4 and Literature column citing the previous works in which these QTLswere found.

	QTL		Full panel		No Meijer		Only Meijer		Literature	
Trait	Name	chr (Mb in DMv6.1)	Haplotags	SNPs	Haplotags	SNPs	Haplotags	SNPs	chr (Mb in DMv4.3 when available)	Reference
Yield	*YLD_C1_19*	*chr01 (71.77)*					*C1_19_00110*		1, 2, 5, 6, 9, 7, 11, 12	Bradshaw *et al*. (2008), McCord *et al*. (2011), Hurtado-Lopez *et al*. (2015), Manrique-Carpintero *et al*. (2015), Rak *et al*. (2017), Schönhals *et al*. (2017), da Silva Pereira, Mollinari, Schumann, *et al*. (2021)
	YLD_C2_4	chr02 (20.99)						chr02_20959684, chr02_20959746		
	**YLD_C3_17**	**chr03 (46.06)**	**C3_17_011000**	**chr03_46058754**	**C3_17_011000**	**chr03_46058754**				
Skin Smoothness	**SkinS_C2_8**	**chr02 (24.47)**			**C2_8_0000001000**	**chr02_24470953**				
	*SkinS_C2_13*	*chr02 (28.96)*	*C2_13_100010000*		*C2_13_100010000*					
	*SkinS_C6_3*	*chr06 (2.68)*	*C6_3_0000101-00*		*C6_3_0000101-00*					
Cooking Type	*CT_C8_21*	*chr08 (51.08)*					*C8_21_0000000000*		1, 2, 6, 9, 10, 11	Kloosterman (2006), D’hoop *et al*. (2008), D’hoop *et al*. (2014)
After-cooking Blackening	ACB_C3_1	Chr03 (3.38)		chr03_337574, chr03_337585					1, 2, 3, 4, 5, 6, 7, 11	Bradshaw *et al*. (2008), D’hoop *et al*. (2008), D’hoop *et al*. (2014)
Dry Matter Content	DM_C3_30	chr03 (59.35)						chr03_59353418	2, 3, 5, 6, 7, 8	Bradshaw *et al*. (2008), McCord *et al*. (2011), da Silva Pereira *et al*. (2021b)
Canopy Stage 1	Can1_C12_0	chr12 (3.78)		chr12_3783754		chr12_3783754				
Canopy Stage 2	*Can2_C1_19*	*chr01 (71.77)*					*C1_19_00110*			
	**Can2_C5_6-C5_8**	**chr05 (4.94-7.25)**	**C5_6_0000000**	**chr05_4941391, chr05_4941406, chr05_7251491, chr05_7251555**			**C5_6_0000000, C5_7_0001111110**	**chr05_4941391, chr05_4941406, chr05_6204154, chr05_7251491, chr05_7251555**		
Enzymatic Browning	*EnzB_C8_18*	*chr08 (48.05)*					*C8_18_0010011001001010100*		1, 3, 4, 5, 6, 7, 8, 11	Werij *et al*. (2007), D’hoop *et al*. (2008), Urbany *et al*. (2011), D’hoop *et al*. (2014)
Tuber Shape	**TSH_C10_7-C10_12**	**chr10 (48-53.13)**	**C10_7_01111000010, C10_8_0001111100, C10_8_0001111110, C10_8_0110000000, C10_9_000101000100000, C10_9_011100000110110**	**chr10_49148246, chr10_49148249, chr10_49148293, chr10_49148305, chr10_49148316, chr10_50323192, chr10_50323244**	**C10_8_0001111100, C10_9_000101000100000, C10_12_00010000000**	**chr10_49148293, chr10_49148305, chr10_49148316, chr10_50323192, chr10_50323244**	**C10_8_0001111110, C10_8_0110000000, C10_9_000101000100000, C10_9_011100000110110,**	**chr10_49148246, chr10_49148249, chr10_49148293, chr10_49148305, chr10_49148316, chr10_49148341, chr10_50323151, chr10_50323153, chr10_50323192, chr10_50323225, chr10_50323244, chr10_50323263**	10 (48.7)	van Eck *et al*. (1994), Rosyara *et al*. (2016), Sharma *et al*. (2018), Pandey *et al*. (2022)
Eye depth	**EYE_C9_21-C9_23**	**chr09 (61.25-62.91)**					C9_21_000100010100, C9_23_000001	**chr09_61254396, chr09_62045322**	3, (53.1), 5 (43.9), 10 (48.6)	Li *et al*. (2005), ŚLiwka *et al*. (2008), Rosyara *et al*. (2016), Sharma *et al*. (2018), Pandey *et al*. (2022)
	**EYE_C10_6-C10_10**	**chr10 (4.74-51.2)**	**C10_9_000101000100000**	**chr10_4741749, chr10_49148293, chr10_49148305, chr10_49148316, chr10_50323244,**		**chr10_4741749, chr10_49148293, chr10_49148305, chr10_49148316**	**C10_8_0001111110, C10_9_011100000110110, C10_9_000101000100000, C10_10_01010000**	**chr10_49148246, chr10_49148293, chr10_49148305, chr10_49148316, chr10_50323151, chr10_50323153, chr10_50323225, chr10_50323244, chr10_50323263,**		
	EYE C11_8	chr11 (8.19)		chr11_8190769						
Maturity	MAT_C1_32	Chr01 (84.18)		chr01_84177600					1, 2, 3, 5, 7	(McCord *et al*. (2011), Kloosterman *et al*. (2013), da Silva Pereira *et al*. (2021b)
	**MAT_C5_6-C5_7**	**chr05 (4.94)**	**C5_6_0000000, C5_6_0011010, C5_7_0001111110**	**chr05_4941391, chr05_4941406, chr05_4941464**	**C5_6_0000000,**	**chr05_4941391, chr05_4941406**	**C5_6_0000000, C5_7_0001111110**	**chr05_4941391, chr05_4941406, chr05_6204154**		
Tuber Length	TPM_C2_4	chr02 (20.96)				chr02_20959691			10 (50), 9	Zhang, Qu, *et al*. (2022)
	**TPM_C10_8-C10_10**	**chr10 (49.15-51.02)**	**C10_8_0001111110, C10_10_00100100**				**C10_8_0001111110, C10_9_000101000100000, C10_9_011100000110110, C10_10_01010000,**	**chr10_49148246, chr10_49148249, chr10_49148293, chr10_49148305, chr10_49148316, chr10_49148341, chr10_50323151, chr10_50323153, chr10_50323192, chr10_50323225, chr10_50323244, chr10_50323263**		
	*TPM_C10_16*	*chr10 (57.02)*	*C10_16_000000000000*							
Total Tuber Number	TTN_C3_29	chr03 (58.36)		chr03_58369063, chr03_58369113		chr03_58369063, chr03_58369113			1, 4, 5, 6, 8, 9, 12	Manrique-Carpintero *et al*. (2015), Zhang *et al*. (2022)
	*TTN_C4_33*	*chr4 (67.1)*	*C4_33_00100010*		*C4_33_00100010*					
	*TTN_C10_12*	*chr10 (52.17)*	*C10_12_00000100000*							
	*TTN_C11_12*	*chr11 (37.67)*			*C11_12_000100000*					
Sensitivity to Common Scab	*Scab_C6_18*	*chr06 (48.3)*	*C6_18_011111111110*		*C6_18_011111111110*				1, 2, 3, 4, 5, 6, 10, 11, 12	Bradshaw *et al*. (2008), Braun *et al*. (2017), Yuan *et al*. (2020), Zorrilla *et al*. (2021), da Silva Pereira, Mollinari, Qu, *et al*. (2021)
Chipping Color 1_8	QDC1-8_C12_11	chr12 (9.26)		chr12_9258876					for “off the field’: 4 (68), 10 (55.2)	Byrne *et al*. (2020)
Chipping Color 2_8	QDC2-8_C10_14	chr10 (55.08)				chr10_55081844			1, 6, 10, 11	Bradshaw *et al*. (2008), D’hoop *et al*. (2014)
Chipping Color 2_4	*QDC2-4_C3_17*	*chr03 (46.06)*			*C3_17_000010*				11, 6,	Bradshaw *et al*. (2008)
	QDC2-4_C7_23	chr07 (50.94)						chr07_50949343, chr07_50949356		
	*QDC2-4_C12_9*	*Chr12 (7.44)*	*C12_9_0010010001100*							
Yellow Flesh Color	**FC_C3_15-C3_20**	**chr03 (44.04-49.75)**	**C3_15_00000000, C3_17_000000, C3_18_00000000, C3_18_00011000, C3_20_000000**	**chr03_44040545, chr03_46058758. chr03_47024967, chr03_47024968, chr03_49750222**	**C3_15_00000000, C3_17_000000, C3_18_00000000, C3_18_00011000**	**chr03_45076941, chr03_47024967, chr03_47024968, chr03_49750222**	**C3_17_000000, C3_18_00000000, C3_18_00011000**	**chr03_47024967, chr03_47024968**	1 (63.8), 3 (44.1), 3 (48.5), 3 (49.3), 3 (50.8)	Sharma *et al*. (2018), Pham *et al*. (2020), Pandey *et al*. (2022)
Yellow Skin Color	*YSC_C2_17*	*chr02 (32.62)*	*C2_17_0010010*							
	YSC_C3_12-C3_13	chr03 (41.3-42.87)		chr03_41301966, chr03_41302030		chr03_41301966, chr03_41302030, chr03_42866260				
Sprout Dormancy	**SD_C2_6**	**chr02 (22.77)**			**C2_6_01101**	**chr02_22766739, chr02_22766770**			1, 3, 4, 5, 6, 7, 8, 11	Bradshaw *et al*. (2008), D’hoop *et al*. (2008), Urbany *et al*. (2011), D’hoop *et al*. (2014)
	SD_C9_11	chr09 (51.47)		chr09_51478036, chr09_51478037, chr09_51478039						

### Differences in QTL detected across populations

Differences in the sets of QTL were observed across populations. Only5 QTL, for Tuber Shape, Eye depth, Yellow Flesh Color (2 QTL) and Maturity were detected across all 3 populations with at least 1 marker shared for each trait across all populations. Another 15 QTL were detected either in the “no Meijer” sub-population (6) or in the “only Meijer” sub-population (9). Eleven QTL were shared between the full panel and one of the other two sub-populations. Ten QTL were detected with the full panel but were not significant in either sub-population.

### Identification of previously characterized QTL

**Fig. 7.**
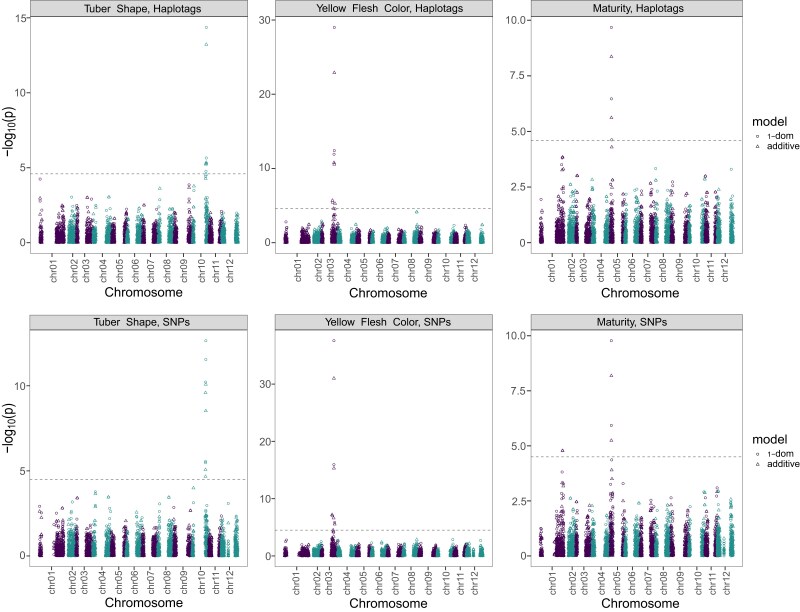
Manhattan plots for the reference traits: Tuber Shape, Yellow flesh color, and Maturity. Top: analysis with Haplotag data. Bottom: analysis with SNP data.

Some of the traits evaluated here, were previously described in detail in the literature and enabled us to validate our approach (Fig. 7). Indeed, we identified a highly significant QTL (-log_10_(*P*-value) = 14.36) on chr10 across the 3 phenotypic datasets for Tuber Shape, which was detected both with SNP and haplotag markers (Fig. 7, Supplementary File 9). This QTL corresponds to the well described *Ro* locus (van Eck *et al*. 1994; van Eck *et al*. 2022).

The well-known *Y* (Yellow) locus and the causal gene involved in yellow flesh color beta-hydroxylase (*Chy2* or *BCH)* (Bonierbale *et al*. 1988; Thorup *et al*. 2000; Brown *et al*. 2006; Wolters *et al*. 2010), One isoform (PGSC0003DMG400009501) of the *Bch* gene was reported to be located at 44.1 Mb in DMv4.03 (Pandey *et al*. 2022) and aligns with position 42.9 Mb on chr03 of the DMv6.1 reference genome sequence (Pham *et al*. 2020). We identified 1 QTL for Yellow flesh color on chr03 spanning the region from 44.04 Mb to 49.75 Mb (PotatoMASH loci C3_15 to C3_20), peaking at 47.03 Mb (PotatoMASH locus C3_18) with LOD score (−log10(*P*-value)) of 44.66 for the significant marker chr03_47024967 (Supplementary File 9). Two additional PotatoMASH loci on chr03 (C3_11 at 40.41 Mb; and C3_30 at 59.35 Mb) also showed significant associations with Yellow Flesh Color.

Furthermore, we also detected a QTL for Maturity on chr05, peaking at 4.94 Mb (PotatoMASH locus C5_6). The haplotag C5_6_0011010 and the 3 SNPs of this haplotag (chr05_4941391, chr05_4941406 and chr05_4941464) were associated with late maturity and the haplotag C5_6_0000000 with early maturity. This QTL is near to the region containing *StCDF1* gene (Soltu.DM.05G005140.1, chr05:4485531..4488495 DMv6.1), which is well established as the gene largely responsible for the plant maturity in potato (Kloosterman *et al*. 2013).

### QTL for agronomic and morphological traits

Eighteen of the QTL identified in this work were confirmed with previous QTL studies in potato at the diploid and tetraploid level (Table 4). We also detected new QTL for complex traits not yet reported before. In total, we discovered 19 novel QTL on 8 chromosomes: 5 QTL on chr02—two QTL for Skin Smoothness, 1 for Sprout Dormancy, 1 for Total Tuber Number, and 1 for Tuber Length. Four QTL on chr03—1 for Yield and 1 for Total Tuber Number. One QTL on chr06 for Skin Smoothness. One QTL on chr07 for Chipping Color. Two QTL on chr08—1 for After-cooking Blackening and 1 for Cooking Type. Two QTL on chr09—1 for Eye depth and 1 for Sprout Dormancy. One QTL on chr10 for Total Tuber Number. Two QTL on chr11—1 for Eye depth and 1 for Total Tuber Number. Three QTL on chr12—1 for Canopy stage 2 and 2 for Chipping Color.

We detected a QTL for Canopy stage 2 (canopy coverage 10 weeks after planting) peaking at the same PotatoMASH locus where the well-known QTL for Maturity was detected, C5_6 (4.94 Mb). The significant haplotag, C5_6_0000000 was associated with earliness and lower canopy cover while SNPs chr05_4941391 and chr05_4941406 were associated with lateness and higher canopy cover. This association between maturity and canopy type is also confirmed by the significant correlation between the phenotypic values of these two traits (*r*^2^ = 0.41). The two additional QTL detected on chr01 and chr12 for early-stage canopy development (6 weeks after planting), could not be associated with plant maturity and seem to be caused by genetically independent loci affecting canopy vigor.

The novel QTL for Yield was detected in chr03, locus C3_17 (46.06 Mb). It was identified in both the full panel and in the sub-population “no Meijer” with a significant SNP chr03_46058754 and with the haplotag specific to this SNP, C3_17_011000, both associated with low yield. A QTL in this region was also detected for Total Tuber Number with the same significant SNP and haplotag both associated with low Total Tuber Number. This could be a new region associated with Yield and yield-related traits and is also supported by the significant correlation between Yield and Total Tuber Number (*r*^2^ = 0.57). Two additional PotatoMASH loci on chr03, C3_7 and C3_29 were associated with low Total Tuber Number although we had not considered them separate loci in the QTL count.

Two additional novel QTL were detected for Total tuber number. One QTL on chr10 at locus C10_12 (53.13 Mb) was identified only in the full panel but not in any of the sub-populations, with a significant haplotag C10_12_00000100000 associated with low Total Tuber Number. No SNP was significant. One QTL was detected on chr11 at locus C11_12 (37.67 Mb), for the “no Meijer” sub-population only, with a significant haplotag C11_12_000100000 associated with low Total Tuber Number. No SNP was significant.

For Tuber Length, we detected one new QTL on chr02 at locus C2_4 (20.96 Mb), with the significant SNP chr02_20959691 with a small positive effect associated with shorter tubers (higher number of tubers per meter, TPM). This association is based on only 127 individuals of the “no Meijer” sub-population. This could be another new region associated with Yield and yield-related traits and is also supported by the significant correlation between Yield and Tuber Length (*r*^2^ = −0.19, high Yield correlates negatively with shorter tubers). A QTL for higher Yield was also detected in the same locus, C2_4, but in the sub-population “only Meijer” and with different SNPs/haplotags suggesting different origins of this locus.

Eye depth is a well-characterized trait and indeed we detected the well-known, large-effect QTL on chr10 in our full panel, spanning across the PotatoMASH loci C10_6 to C10_10 (4.74 to 51.2 Mb) peaking at 50.32 Mb. The deep eye (Eyd) phenotype was found to be associated with round tubers (Ro) (Li *et al*. 2005). The Eyd/eyd locus is located on chr10 and is closely linked with the major locus for Tuber Shape (Ro/ro). In the QTL detected here, the significant haplotags C10_8_0001111110, C10_9_011100000110110, and C10_10_01010000 and SNP alleles chr10_49148246, chr10_49148293, chr10_49148305, chr10_49148316, chr10_50323151, chr10_50323153, chr10_50323225, chr10_50323244, and chr10_50323263, were all associated both with deep eyes and round tubers, being their effects consistent with the genetics known (Li *et al*. 2005). In the opposite direction of effect, we found C10_9_000101000100000 associated with flat eyes and long tubers. We also detected a novel QTL for Eye depth on chr11 at C11_8 (8.19 Mb), with a significant SNP chr11_8190769, associated with deep eyes. No specific haplotag was detected.

Skin Smoothness is a complex trait, and many complementary factors influence tubers’ skin texture, such as soil and climate. Earlier genetic studies by De Jong (1981) involved skin russeting as a phenotypic category, but in our panel no russeting phenotype was observed. In our study, only the skin texture was phenotyped, using a scoring scheme ranging from rough skin to smooth skin. Therefore, our study is the first to identify QTL for Skin Smoothness with no russeting. We detected three QTL for Skin Smoothness: Two QTL were detected on chr02: in PotatoMASH locus C2_8 (position 24.47 Mb) with the significant SNP chr02_24470953 of the haplotag C2_8_0000001000, and in locus C2_13 (position 28.96 Mb) with the significant haplotag C2_13_100010000. Both QTL were associated with smoother skin. The third QTL was found on chr06 at locus C6_3 (position 2.86 Mb), where the haplotag C6_3_0000101-00 was associated with rough skin, but no specific SNP allele underlying this haplotag was significant.

We detected a QTL for Sensitivity to Common Scab on chr06 at locus C6_18 (position 48.3 Mb). The significant haplotag C6_18_011111111110 was associated with susceptibility to Common Scab. This haplotag allele was present in only three individuals of the sub-population “no Meijer”. The specific SNP for this haplotag (the only SNP not shared by the other haplotags in C6_18) was chr06_48297069 but was not statistically significant, most likely due to high missing data in this position (70%). We present this marker allele here, as a potential source for negative selection in future breeding, but further investigation needs to be done for validation.

Cooking Type is a complex trait. Previous studies revealed multiple QTL on multiple chromosomes: ch01, ch02, ch06, ch09, ch10, and ch011 (Kloosterman 2006; D’hoop *et al*. 2008; D’hoop *et al*. 2014). Our study is the first to report a QTL on chr08, at locus C8_21 (position 51.08 Mb), which was detected only for the “only Meijer” sub-population, with the significant haplotag C8_21_0000000000 associated with flouriness. No SNP was significant.

We detected 5 QTL for Chipping Color measured after three storage conditions (Table 1). For Chipping Color after storage at 8°C for 4 months before crisping, we identified 1 novel QTL on chr12 at locus C12_11 (position 9.26 Mb) with the full panel. The significant SNP allele, chr12_9258876, was associated with the dark color of crisps. This SNP allele is shared by a few haplotags, and none of these haplotags was significant.

For Chipping Color after storage at 8°C for 6 months before crisping, we detected 1 QTL on chr10 at locus C10_14 (position 55.08 Mb) with the sub-population “no Meijer”. The significant SNP allele, chr10_55081844, with a positive effect was associated with a light, pure color of crisps. This SNP allele is shared by two haplotags, but none of the haplotags was significant. A previous work with tetraploid clones collected from the breeding program in Teagasc (Ireland) for “off the field” fry color, detected a large effect QTL on chr10 peaking at 56.16 Mb in DMv6.1 (55.28 Mb in DMv4.3) (Byrne *et al*. 2020). Our significant SNP allele at chr10 is at 55,081,844 bp is approximately 1 Mb distance from the one identified by Byrne *et al* (2020).

Three additional novel QTLs for Chipping Color were detected for storage at lower temperature (4°C) for 6 months. One QTL mapped on chr03 at locus C3_17 (position 46.06 Mb) with the sub-population “no Meijer” with the significant haplotag C3_17_000010 associated with dark color of crisps. Two other QTL were detected on this same locus for low Yield and Total Tuber Number, but with a different haplotag, C3_17_011000. Related to this, we found a negative correlation between Yield and Chipping Color 2_4 (*r*^2^ = −0.23). We observed that only a small portion of the population (∼4%) was heterozygous for those 2 alleles, possibly affecting this correlation, but we did not find a significant correlation between Chipping Color and Tuber Number. The second QTL associated with a light, pure color of crisps was detected on chr07 at locus C7_23 (position 50.94 Mb) with the sub-population “only Meijer”, specifically with the significant SNPs alleles chr07_50949343 and chr07_50949356. Those two SNP alleles are in complete LD but are dispersed in many haplotags and no haplotag resulted statistically significant. The third QTL was detected on chr12 at locus C12_9 (position 7.44 Mb) using the full panel, with the significant haplotag C12_9_0010010001100 associated with a darker color of crisps. No SNP allele was significant.

It is useful to remember that the germplasm panel is derived from several independent commercial potato breeding programmes. We did not do an extensive analysis of each population source separately, but the fact that some QTL were exclusively discovered in one or the other of the “sub-populations”, suggests that beneficial alleles in one population may have the potential to augment genetic gain in populations lacking those alleles (alternatively, in some populations, during the breeding efforts, those alleles were successfully purged, or simply never possessed some undesired effect alleles). For example, the 4 significant SNP alleles and haplotags, chr09_61254396, chr09_62045322, C9_21_000100010100, and C9_23_000001, all associated with the undesirable trait of deep eyes in the QTL EYE_C9_21-C9_23, were only found in the “only Meijer” sub-population. Those markers co-segregate in the same 25 individuals (∼13%), and it is possible that they all originated from the same source with deep eyes. On the other hand, in the “no Meijer” population, the SNP chr09_61254396 is present in 11 (∼3%) individuals, the SNP chr09_62045322 is only present in 7 individuals (∼2%), the haplotag C9_21_000100010100 is present in 9 individuals (2.4%) and C9_23_000001 is not present at all. This suggests that the 2 sub-populations do not share the same ancestor or that the subpopulation “no Meijer” have successfully selected against this negative allele. Another example is in the significant haplotag C6_18_011111111110 associated with susceptibility to common Scab that is only present in three individuals in the “no Meijer” sub-population.

### Differences in QTL detected with SNPs vs haplotags

**Fig. 8.**
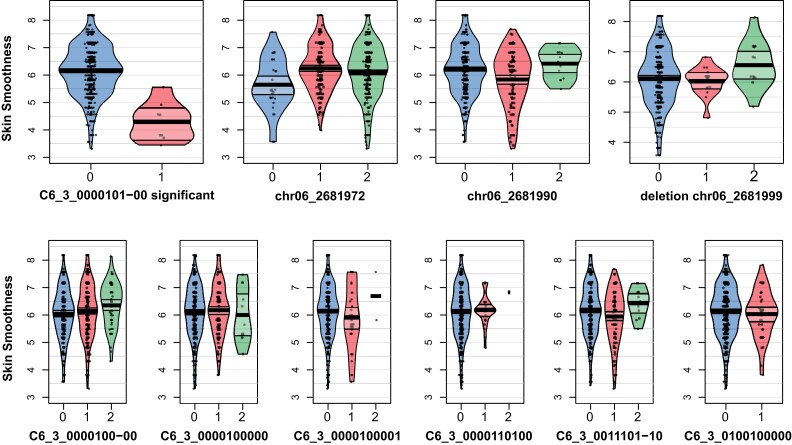
Allele dosage in QTL C6_3 vs the effect on skin smoothness. Top: Allele dosage of the significant haplotag and the nonsignificant SNPs underlying this haplotag. Bottom: other six haplotags of this region that possess those SNPs but their combination in the haplotags was not associated with the trait.

**Fig. 9.**
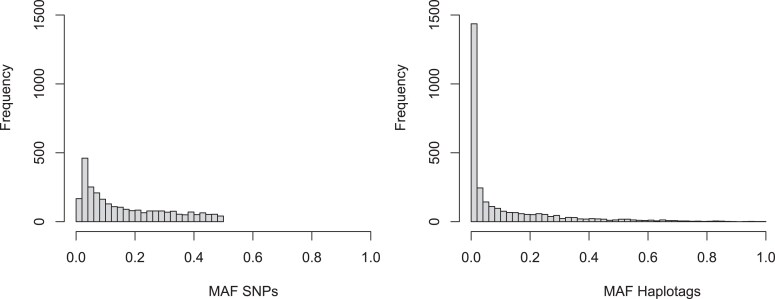
Minor Allele frequency (MAF) distribution of 2,730 SNPs (left) and 2,995 haplotags (right).

Fourteen of the QTL were detected by haplotags only, 13 QTL were identified with SNP data only, and 10 QTL were discovered with both SNPs and haplotags. To gain a better understanding of the ability to detect QTL with either SNPs or haplotags, we manually re-examined all individual QTL. We observed that in most cases of QTL detected with the haplotags only, the significant haplotag presents a specific composition of SNPs, but each individual SNP is dispersed across multiple haplotags of different SNPs compositions. In Table 5, we present 4 examples of this phenomenon. This is also visible when looking at the dosage effect of the markers. One example for this is the new QTL discovered for Skin Smoothness, where the significant haplotag C6_3_0000101-00 has negative effect, while none of the underlying SNPs have a significant effect nor the other haplotags composed by the same SNPs (Fig. 8). In the opposite scenario of the QTL detected only with the SNP data, we observed that the significant SNP was shared in many haplotags (Table 6), which have a lower frequency in the population than the frequency of the significant SNP. To understand this phenomenon, we looked at the minor allele frequency of both SNPs and haplotags and observed that the minor allele frequency in the case of the SNPs is greater than 1% for most SNPs. When looking at haplotags, the frequency of individual haplotypes is much lower, with approximately 1,200 of the haplotags have a frequency below 1% (Fig. 9).

**Table 5.** Four examples of QTL detected by one unique haplotag and not with any of its constituent SNPs. The number of individuals carrying each SNP/haplotag allele is indicated within brackets.

Trait	Significant haplotag (number of individuals)	Underlying SNPs (number of individuals)	Nontrait-associated haplotags sharing the same underlying SNPs (number of individuals)
Total tuber number	C11_12_000**1**00000 (63)	chr11_37673981 (189)	C11_12_000**1**01000 (5) C11_12_001**1**00000 (11) C11_12_010**1**00000 (1) C11_12_010**1**01000 (138)
Skin Smoothness	C6_3_0000**1**0**1-**00 (9)	chr06_2681972 (549) chr06_2681990 (163) deletion_chr06_2681999 (489)	C6_3_0000**1**00**-**00 (329) C6_3_0000**1**0000- (1) C6_3_0000**1**00000 (182) C6_3_0000**1**00001 (38) C6_3_0000**1**10100 (29) 6_3_0011**1**0**1-**10 (148) C6_3_0100**1**00000 (42)
Skin Smoothness	C2_13_1000**1**0000 (15)	chr02_28958095 (310)	C2_13_0000**1**0000 (164) C2_13_0000**1**1000 (11) C2_13_001011000 (126)
Chipping Color 2_4	C12_9_00**1**00**1**000**11**00 (214)	chr12_7435710 (464) chr12_7435746 (531) chr12_7435801 (423) chr12_7435802 (464)	C12_9_00**1**00**1**001**11**00 (7) C12_9_00**1**00**1**100**11**10 (38) C12_9_00**1**00**1**100**11**10 (108) C12_9_00**1**10**1**001**11**00 (11) C12_9_00000**1**0000000 (64) C12_9_00010**1**0000000 (8) C12_9_00010**1**0100000 (97)

**Table 6.** Six QTL detected with SNPs but not with the haplotag dataset, and all the haplotags composed by those SNPs. The number of individuals for each SNP/haplotag is given within brackets.

Trait	QTL	Significant SNPs (number of individuals)	Haplotags sharing this SNPs (number of individuals)
Dry matter content	DMC C3_30	chr03_59353418 (40)	C3_30_0000**1**00 (12), C3_30_0110**1**00 (20), C3_30_0110110 (2), C3_30_0111**1**00 (3)
Chipping color 1_8	QDC1_8 C12_11	chr12_9258876 (281),	C12_13_0**1**00000 (255), C12_13_0**1**10000 (32)
Total tuber number	TTN C3_29	chr03_58369063 (458), chr03_58369113 (550)	C3_29_00**1**000**1**0 (409), C3_29_00**1**010**1**0 (5), C3_29_00**1**100**1**0 (69), C3_29_01**1**100**1**0 (29), C3_29_01**1**101**1**0 (2), C3_29_01**1**111**1**0 (293)
Total tuber number	TTN C3_7	chr03_37250410 (191)	C3_7_000**1**110 (21), C3_7_001**1**110 (143), C3_7_0101010 (29)
Eye depth	EYE C11_8	chr11_8190769 (239)	C11_8_0010011**1**000 (84), C11_8_0010011**1**100 (82), C11_8_0110111**1**000 (73)
Chipping Color 2_4	QDC2_4_C7_23	chr07_50949343 (155), chr07_50949356 (156)	C7_23_000**11**1000 (71), C7_23_0100**1**0000 (2), C7_23_010**11**0000 (30), C7_23_010**11**0010 (45), C7_23_010**11**1000 (3)

## Discussion

Genetic improvement of potato at the diploid level is experiencing a resurgence, largely driven by the use of alleles that can overcome the gametophytic self-incompatibility system in diploid material, allowing the development of strategies to rapidly accumulate and fix traits in a manner not possible at the tetraploid level. The primary goal of this study was to genetically characterize a large pool of diploid potato breeding material that is at the foundation of the diploid breeding efforts of several commercial breeding programmes that are engaged in a collaborative initiative towards innovative potato breeding schemes, combining the analytical breeding strategy (Chase 1963), which makes use of diploids to facilitate genetic studies and selection before returning to the tetraploid level through interploidy crosses, with self-compatibility.

### Phenotypic data

The diploid clones used in this study represent a very diverse collection and the commercial traits display a wide range of phenotypic trait values. In Table 2, we show that for each trait the full scale of trait values was observed, indicative of primitive material, primary dihaploids with compromised vigor, as well as elite material. On average poor trait values were observed for quality traits such as discoloration due to Enzymatic Browning and Chipping Color; notoriously difficult traits to improve. Most clones displayed the firm Cooking Type, which is negatively correlated with (and largely due to) low values for Dry Matter Content. Canopy development, Tuber Shape and Sprout Dormancy are among the most diverse traits. Most clones had relatively round and uniform Tuber Shape and late Maturity. Late maturity is considered beneficial to obtain an extended period of flowering, which facilitates making crosses during the breeding program, which may explain its prevalence in early-stage prebreeding material. However, early maturity is desirable for several market classes of potato. Since early maturity is largely controlled by a single large-effect quantitative trait locus, the effort to regain early maturity should be relatively easy as material advances to more commercial status over cycles of selection (Song and Endelman 2023).

Broad sense heritabilities varied mostly between moderate to high values, ranging from 50 to 90% across all traits. Sensitivity to Common Scab had, on average, the lowest *H*^2^ across companies (50%), suggesting either low reproducibility of disease development across years due to lack of exposure to the pathogen, environmental factors, or that different trial fields used across years are infested by different isolates. This is also visible with the performance of the controls over the years and between companies, where we can see a large variance in the scoring of Sensitivity to scab even in the same year and the same company (Supplementary File 6). Moderately low average *H^2^* values, ranging between 60 and 75%, are typical for traits such as Presentability of Tubers, Skin Smoothness, Skin Brightness, and Tuber Regularity, which have a somewhat ambiguous trait definition and a scale that is not objectively measurable, but rather a result of the so-called “breeders’ eye”. Despite the potential subjectivity of these scores, the *H*^2^ values obtained suggest high repeatability. Genomic heritabilities were also calculated (Supplementary File 7) to catch a more accurate estimate than the average presented in Table 2. However, the results were much lower than expected, with, for example, heritability for tuber shape of 0.41 and 0.4. It could be that the tools available to calculate genomic heritability are not suited to the low number of marker sets we use in this study. This should be explored further in future analyses.

Correlation analysis of all the trait pairs was performed to examine associations between traits (Fig. 2). The traits of Skin Smoothness and Skin Brightness, albeit representing a subjective breeder's score, show the highest correlation of 0.76, suggesting that the trait definitions are somewhat arbitrarily different, or share similar underlying aspects. Both traits also correlate with Yellow Skin Color (0.48 and 0.56), where darker skins imply thicker skin. Another pair of traits: Tuber Regularity and Presentability show a Pearsons correlation of 0.66. This is not unexpected because Regularity is an aspect within Presentability, along with Eye depth (*r*^2^ = 0.43 and 0.46). Three processing traits related to Chipping Color also show high pairwise correlations (0.59, 0.71, 0.75), suggesting that the storage regime of tubers, causing cold sweetening, is less important than the initial Chipping quality at harvest. Byrne *et al*. (2020) made a similar observation on a similarly sized population of tetraploid breeding clones from a single commercial breeding programme. In this diploid gene pool, an unexpected positive correlation of 0.57 was found between Yield and Total Tuber Number. Such a correlation would be rather unexpected for a panel of varieties, selected for yield above a certain threshold. Maturity and Canopy development also show expected correlations where late maturity leads to bigger canopies at both stages (0.34, 0.41) and the Canopy-Yield correlation was 0.25 and 0.46. However, the negative correlation between Yield and Maturity is unexpected (−0.25). Canopy stage 1 and Canopy stage 2 correlates with Total Tuber Number (0.18 and 0.37), which could be due to a common plant architecture, where stronger above- and below-ground branching patterns or stems and stolons may contribute to larger canopy cover and tuber number.

In general, this “snapshot” of the extent of phenotypic diversity in this genepool suggests that variability exists for most important agronomic and quality traits, and further selection in all or individual parts of the panel is expected to allow improvement. In terms of the use of this material in strategies involving inbreeding, we also surveyed the material for the presence of diagnostic KASP markers for the *Sli* locus as described by Clot *et al*. (2020), and found that *Sli* is relatively common in the material, present in 17.5% of the individuals (data not shown). The presence of this locus throughout the material means that efforts to introgress it from exotic sources, with the accompanying issues such as increased timescales of the breeding process and potential linkage drag of unfavorable loci, are unnecessary.

The germplasm panel is composed of material from six different breeding programmes from The Netherlands, Germany, Denmark, and France. These programmes have a mixture of market class targets, including starch, table (domestic and export), processing (crisps and French fries), and specialty (e.g. salad) potatoes. Genome wide marker studies in the European cultivated potato genepool have previously shown some stratification for geographic origin of breeding programme and utility class (Uitdewilligen *et al*. 2015). However, this assessment is not strong, probably due to the relatively recently shared pool of progenitors of the material. When we examined the population structure of our panel, we found two highly distinct groups, one characterized by material from the breeding company Meijer and the other comprising all other material. This is interesting given the fact that diploid material from many Dutch breeding companies, including Meijer, has often originated from the diploid prebreeding programme at WUR, whereas these groups were quite distinct in our analysis. Whilst we treated the panel as two subpopulations for some analyses on this basis, there was also some visible stratification (along the second principal coordinate in Fig. 5) between the other companies. This did not correspond to either market class or geographical location of the programme.

In this experiment, different sets of diploid potato clones were grown at different locations, and trait values were evaluated by different observers. The same 2 or 4 control varieties were included in each trail to allow a fair comparison of phenotypic values. D’hoop *et al*. (2011) already compared phenotypic means from different experimental designs. One of their approaches made use of historical observations, on company specific candidate varieties, retrieved from breeder's field books. In their other approach, all clones (now released as variety) were grown together in a balanced trial with two locations (sandy and clay soil), both with two replications. That study showed that either a single-year balanced field trial, or multiyear–multilocation breeders’ records yield robust phenotypic information that can be used in a genome-wide association study (D’hoop *et al*. 2011). In this study, the differences between company specific panels of diploids were controlled with structure. In particular, the material offered by Meijer was also treated separately. Regarding location differences, in terms of plant material and environmental conditions, the locations may have added variance to the error term, and we may have lost some power but also false negatives.

### GWAS with an amplicon sequencing technique and short read haplotypes

As mentioned earlier, the primary goal of the study was to characterize the foundation breeding material that will contribute to future diploid breeding approaches focused on trait fixation through inbreeding in potato. We focused on agronomic and quality traits that breeders routinely monitor during selection in order to develop a capacity to increase the effectiveness of this process using genome-based methodologies in future. Based on the marker-inferred population structure, we preformed 6 QTL discovery analysis: for the entire population, the 2 sub-populations and with the 2 marker sets, SNPs and haplotags. We identified a total of 37, nonredundant QTL. Discovery of these QTL in the population gives us the potential to manipulate their configuration in future material, for instance, to accumulate and fix beneficial alleles or eliminate detrimental alleles. One potential problem with this approach in previous GWAS studies is that associated SNP markers, whilst associated with traits, may still be dispersed amongst different haplotype blocks in which the effective allele underlying the trait is also variably present. In addition to QTL discovery within this prebreeding panel, the study also allowed us to further explore whether the multiallelic discrimination power of PotatoMASH short read haplotypes (haplotags) can resolve this. The general approach certainly seems promising.

An average of approximately 9 haplotags was observed per PotatoMASH locus (range 2–30). This exceeds the average number we previously detected in a panel of tetraploid breeding clones (average of 6, range 2 to 14) (Leyva-Pérez *et al*. 2022). This greater diversity may result from the wider set of utility classes being surveyed, and because at least some of the diploid material is the result of introgression breeding for resistance loci from wild species. In our previous study on the tetraploid panel, we empirically illustrated the hypothesis that haplotags better represent the actual underlying allelic variation at a locus and may offer advantages over bi-allelic SNPs for QTL detection. We posited that this was due to better representation of regions of identity by descent harboring the causal allelic variant of the QTL, and that haplotags are more likely to be in LD with allelic variants of genes with an effect on trait values. Conversely, some or all of the component SNPs may be dispersed across multiple haplotypes, some of which are not in LD with the effective QTL allele.

In our study, 14 of the QTL were detected by haplotags only, 13 QTL were detected with SNP data only, and 10 QTL were detected with both datasets. We found that in most cases, these differences were due to the genetic architecture: the first situation of QTL detected with the haplotags data only, which was our original expectation, occurs when more unique haplotags are in greater LD with QTL causal alleles, whereas the bi-allelic SNPs were dispersed across multiple haplotags, some of which were not in LD with the effective QTL allele (Table 5, Fig. 8).

A marker allele has to be present at sufficient frequency to support association via a statistical test. We observed much higher number of rare haplotags in our population than low-frequent SNPs (Fig. 9). Therefore, for some traits, we will fail to identify statistically significant associations with haplotags with very low frequencies, which can result in the opposite phenomenon, where a QTL is only identified with SNP data. Only when a haplotag coincides with a haplotype specific SNPs their power to detect a QTL is equal.

### Technical limitations affecting the power of QTL detection

The ability to detect QTL with one marker type over the other also depends on the analytical tools we use. In specific cases, we encountered that some features of our genotyping platform limited the QTL detection either with the haplotag dataset or the SNP dataset

**1)** PotatoMASH regions are designed to be single copy based on the DM reference genome. However, if some potato clones possess duplications with allelic variants of these regions, the reads of both copies may map back to the single copy reference genome sequence region during read mapping (since DM is also used as the reference sequence). This may be difficult to see in the SNP data, but can cause more than the expected two haplotypes in individuals with the duplication. SMAP will reject calling haplotags in these cases, assigning a missing value to that individual, and effectively filtering out instances in which this occurs. For example, in region C8_18, we detected a QTL for Enzymatic Browning in the Meijer population with the haplotag data, but not with SNP data. The SMAP locus correctness score of C8_18 was 60, suggesting the existence of additional read variation mapping to that locus (more than 2 haplotypes) that could not be explained as a single bi-allelic locus for 30% of the individuals. Thus, the SNP calling in this locus would be wrong, but SMAP correctly rejected calling haplotags for this locus in those individuals and the association for Enzymatic Browning in this locus is based on the remaining 60% of the population.
**2)** A SNP allele is significant and is in LD with 1 haplotag but the haplotag is not significant. Close inspection of the two QTL where this occurred showed that the significant SNP allele was present in a low number of individuals and associated as a minor effect QTL allele, for which the logarithm of the odds-score (LOD-score) was just above the threshold (Supplementary File 10, doi:10.6084/m9.figshare.26163616.v2). In this case, if some individuals have missing data for that locus, the haplotag cannot be called and results in a missing value. Therefore, the LOD-score of the specific haplotag may not pass the significance threshold. This could affect in two ways, either that asymmetric missing data play a role, where one allele suffers more missing data or in a symmetric way that both alleles suffer the missing data, and the amount of data is simply too low to form a strong statistical test. One example for that is the SNP chr02_20959691 that was significant for Tuber Length at locus C2_4, but there was a lot of missing data for both markers sets in this position, so the association is based on 127 individuals. The haplotag C2_4_0001000000 is specific to this SNP but was not significant; we observed that out of those 127 individuals, SMAP failed to call haplotags for 11 individuals in this locus and this probably affected the mean phenotypic score of the allelic categories, and the association that was already weak in the first place, was lost when using haplotag data.

## Concluding remarks

Although we do not view the number of SNPs we used as “optimal” for GWAS, we were, in fact, testing the hypothesis that haplotags would detect loci not detected by the component SNPs that were used to derive them. We conclude that short read haplotags can detect additional QTL not detectable by individual SNPs, but that, for the various reasons outlined, the opposite is also true. Thus, the approach we adopted, utilizing both sets of data (even though 1 is derived from the other) is the most optimal for QTL detection. One obstacle we faced when using haplotags for the GWAS is that we had to use them as “pseudoSNPs” to employ standard analysis software, and in this study, we have not explored the full potential of the multiallelic nature of the haplotags. From both a genetic and practical breeding point of view, it would be interesting to gain a better understanding of the nature of allelic interactions within and between loci. Recently, Thérèse Navarro *et al.* (2022) developed a software, mpQTL, for QTL analysis at any ploidy level under biallelic and multiallelic models, but for multiparental populations. Their approach was demonstrated with simulated data of short-range haplotypes of autotetraploid multiparental populations. Combining approaches like this with real-world data of higher genetic diversity panel like the current study, will give insights into the genetic control of traits in highly heterozygous systems.

The increased precision offered by the new paradigm in potato breeding means that genome-based tools will become more effective in augmenting selection, allowing the “shepherding” and subsequent fixation of multiple desirable alleles into single genotypes (or the elimination of detrimental alleles). To this end, we have characterized a panel partially representative of the foundational genepool of the future of diploid breeding across several potato-breeding programmes involved in collaborative efforts in this area.

The availability of low-cost, medium-density genotyping approaches capable of generating genome-wide multiallelic marker data in potato (e.g. PotatoMASH in this study, or the Potato DArTag EiB 1.0 generated by CGIAR https://excellenceinbreeding.org/) demonstrate that it is becoming feasible to implement such systems in breeding selection, implying the routine application to thousands of individuals per annum. These marker panels are also amenable to the addition of trait-specific markers, such as those targeting disease and pest resistance loci. We envisage a future where such assays can be used for a combination of marker assisted selection, genomic selection and monitoring the genomic constitution of inbred lines in terms of global and local homo/heterozygosity in potato breeding.

## Supplementary Material

jkae164_Supplementary_Data

## Data Availability

All data necessary for confirming the conclusions of the article are present within the article's text, figures, and tables and the supplementary files. Scripts and intermediate files for the PotatoMASH bioinformatics pipeline are also available at https://doi.org/10.6084/m9.figshare.c.6926560. The code to reproduce the results and figures of this article are available at https://doi.org/10.6084/m9.figshare.c.6937662. Supplemental material available at G3 online.
