# Supplementary material for: QTL discovery for agronomic and quality traits in diploid potato clones using PotatoMASH amplicon sequencing
Source: G3 (Bethesda). 2024 Jul 19;14(10):jkae164. doi: 10.1093/g3journal/jkae164 (PMC11457057; doi:10.1093/g3journal/jkae164)
Supplement: jkae164_Supplementary_Data [file jkae164_supplementary_data.zip › Supplemental_File_2_G3-2024-405051.docx]

**Vexler et al. QTL discovery for agronomic and quality traits in a panel of diploid potato clones using PotatoMASH amplicon sequencing.**

Supplementary file 2 - Correction table for the number of tubers

**Tuber Size Synonym: Tuber Length**

**Synonym: Number of Tubers Per Meter (TPM)**

*Ontology reference:* <http://www.cropontology.org/term/CO_330:0000353/> *(Tuber size)*
*The method of determination used here is different.*

To determine the average length per tuber, several full grown tubers are randomly selected and placed one after the other in the longitudinal direction in a PVC (rain) gutter over the distance of 1 meter. tIf the last tuber inserted, ends up at, for example, 96 cm, the exact value can be looked up in Table 1.

If tuber numbers are limited (less than a meter length) or tuber size is below the preferred size, measurements should still be taken, but notes added into the comments or metadata. If no tubers at all are available this should be recorded as well.

**Table 1**: Correction table for the number of tubers with a deviating length of 100 cm. Example, if 11 tubers end at 97.5 cm, the following is entered as "number of tubers per 100 cm": 11.3

| **# tubers** | **90** | **92,5** | **95** | **97,5** | **100** | **102,5** | **105** | **107,5** | **110** |  |
| --- | --- | --- | --- | --- | --- | --- | --- | --- | --- | --- |
| **4** | 4,4 | 4,3 | 4,2 | 4,1 | 4,0 | 3,9 | 3,8 | 3,7 | 3,6 | **4** |
| **5** | 5,6 | 5,4 | 5,3 | 5,1 | 5,0 | 4,9 | 4,8 | 4,7 | 4,5 | **5** |
| **6** | 6,7 | 6,5 | 6,3 | 6,2 | 6,0 | 5,9 | 5,7 | 5,6 | 5,5 | **6** |
| **7** | 7,8 | 7,6 | 7,4 | 7,2 | 7,0 | 6,8 | 6,7 | 6,5 | 6,4 | **7** |
| **8** | 8,9 | 8,6 | 8,4 | 8,2 | 8,0 | 7,8 | 7,6 | 7,4 | 7,3 | **8** |
| **9** | 10,0 | 9,7 | 9,5 | 9,2 | 9,0 | 8,8 | 8,6 | 8,4 | 8,2 | **9** |
| **10** | 11,1 | 10,8 | 10,5 | 10,3 | 10,0 | 9,8 | 9,5 | 9,3 | 9,1 | **10** |
| **11** | 12,2 | 11,9 | 11,6 | 11,3 | 11,0 | 10,7 | 10,5 | 10,2 | 10,0 | **11** |
| **12** | 13,3 | 13,0 | 12,6 | 12,3 | 12,0 | 11,7 | 11,4 | 11,2 | 10,9 | **12** |
| **13** | 14,4 | 14,1 | 13,7 | 13,3 | 13,0 | 12,7 | 12,4 | 12,1 | 11,8 | **13** |
| **14** | 15,6 | 15,1 | 14,7 | 14,4 | 14,0 | 13,7 | 13,3 | 13,0 | 12,7 | **14** |
| **15** | 16,7 | 16,2 | 15,8 | 15,4 | 15,0 | 14,6 | 14,3 | 14,0 | 13,6 | **15** |
| **16** | 17,8 | 17,3 | 16,8 | 16,4 | 16,0 | 15,6 | 15,2 | 14,9 | 14,5 | **16** |
| **17** | 18,9 | 18,4 | 17,9 | 17,4 | 17,0 | 16,6 | 16,2 | 15,8 | 15,5 | **17** |
| **18** | 20,0 | 19,5 | 18,9 | 18,5 | 18,0 | 17,6 | 17,1 | 16,7 | 16,4 | **18** |
| **19** | 21,1 | 20,5 | 20,0 | 19,5 | 19,0 | 18,5 | 18,1 | 17,7 | 17,3 | **19** |
| **20** | 22,2 | 21,6 | 21,1 | 20,5 | 20,0 | 19,5 | 19,0 | 18,6 | 18,2 | **20** |
| **21** | 23,3 | 22,7 | 22,1 | 21,5 | 21,0 | 20,5 | 20,0 | 19,5 | 19,1 | **21** |
| **22** | 24,4 | 23,8 | 23,2 | 22,6 | 22,0 | 21,5 | 21,0 | 20,5 | 20,0 | **22** |
| **23** | 25,6 | 24,9 | 24,2 | 23,6 | 23,0 | 22,4 | 21,9 | 21,4 | 20,9 | **23** |
|  | **90** | **92,5** | **95** | **97,5** | **100** | **102,5** | **105** | **107,5** | **110** |  |
