# Supplementary material for: QTL discovery for agronomic and quality traits in diploid potato clones using PotatoMASH amplicon sequencing
Source: G3 (Bethesda). 2024 Jul 19;14(10):jkae164. doi: 10.1093/g3journal/jkae164 (PMC11457057; doi:10.1093/g3journal/jkae164)
Supplement: jkae164_Supplementary_Data [file jkae164_supplementary_data.zip › Supplemental_File_6_G3-2024-405051.pdf]

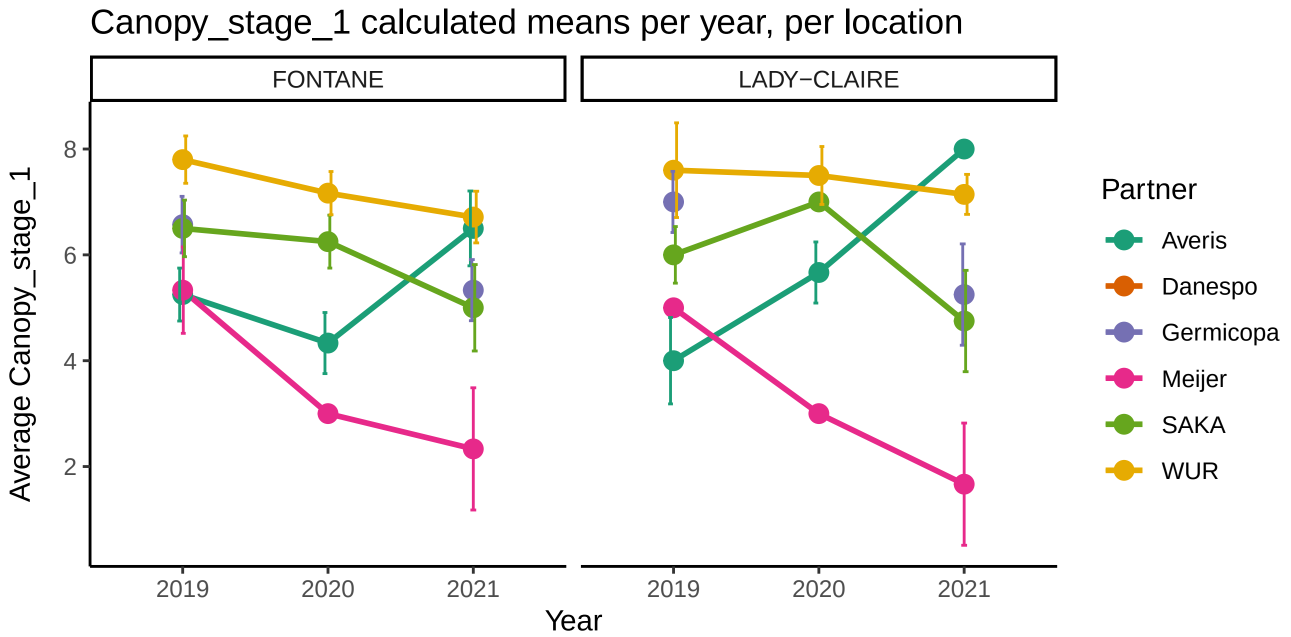
Canopy_stage_1 calculated means per year, per location
FONTANE
LADY−CLAIRE
Average Canopy_stage_1
8
6
4
2
2019
2020
2021
Year
Partner
Averis
Danespo
Germicopa
Meijer
SAKA
WUR

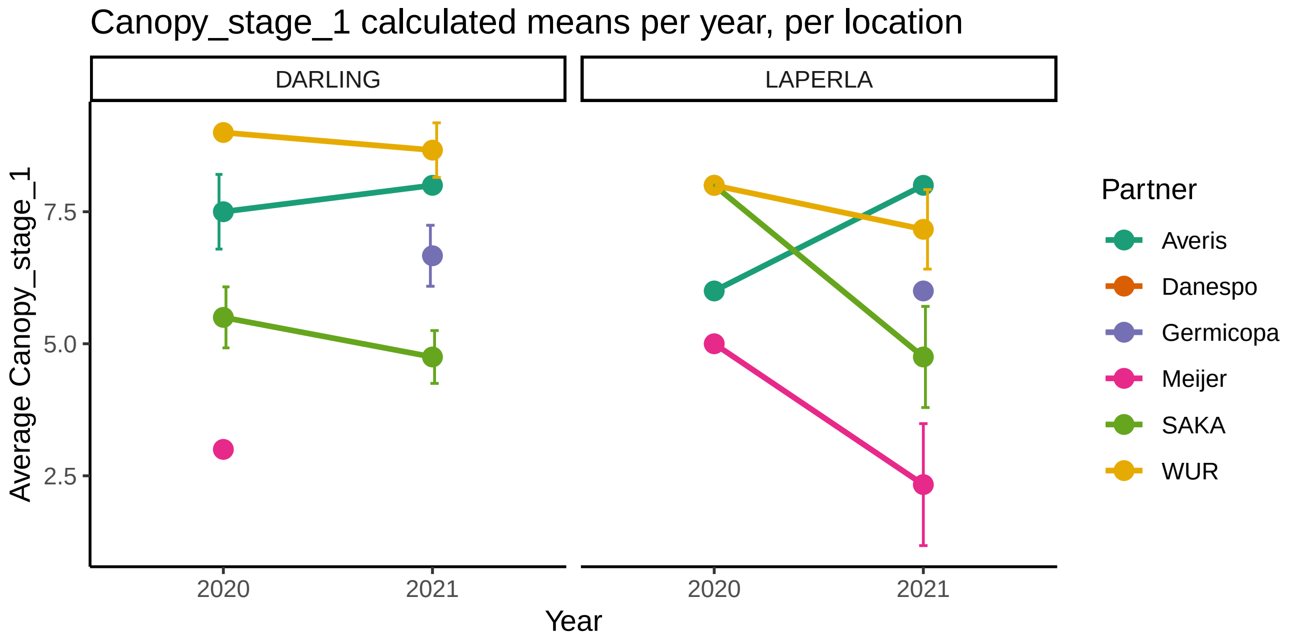
Canopy_stage_1 calculated means per year, per location
DARLING
LAPERLA
Average Canopy_stage_1
7.5
5.0
2.5
2020
2021
Year
Partner
Averis
Danespo
Germicopa
Meijer
SAKA
WUR

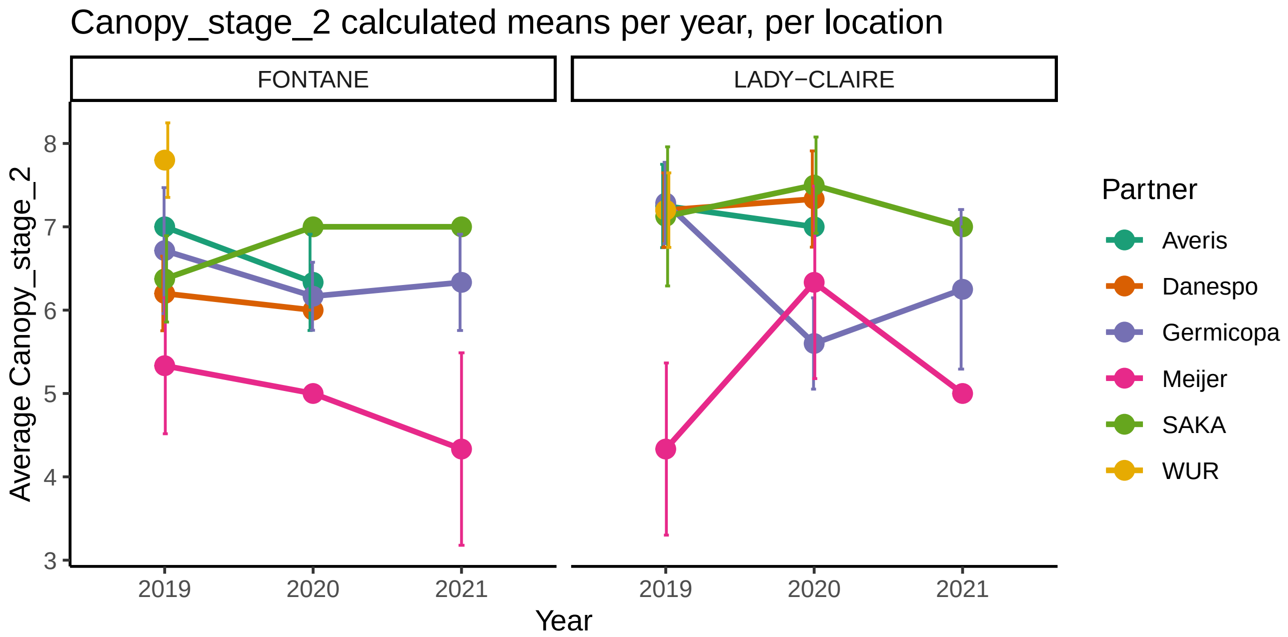

Canopy_stage_2 calculated means per year, per location
FONTANE
LADY−CLAIRE
Average Canopy_stage_2
Year
2019
2020
2021
Partner
Averis
Danespo
Germicopa
Meijer
SAKA
WUR

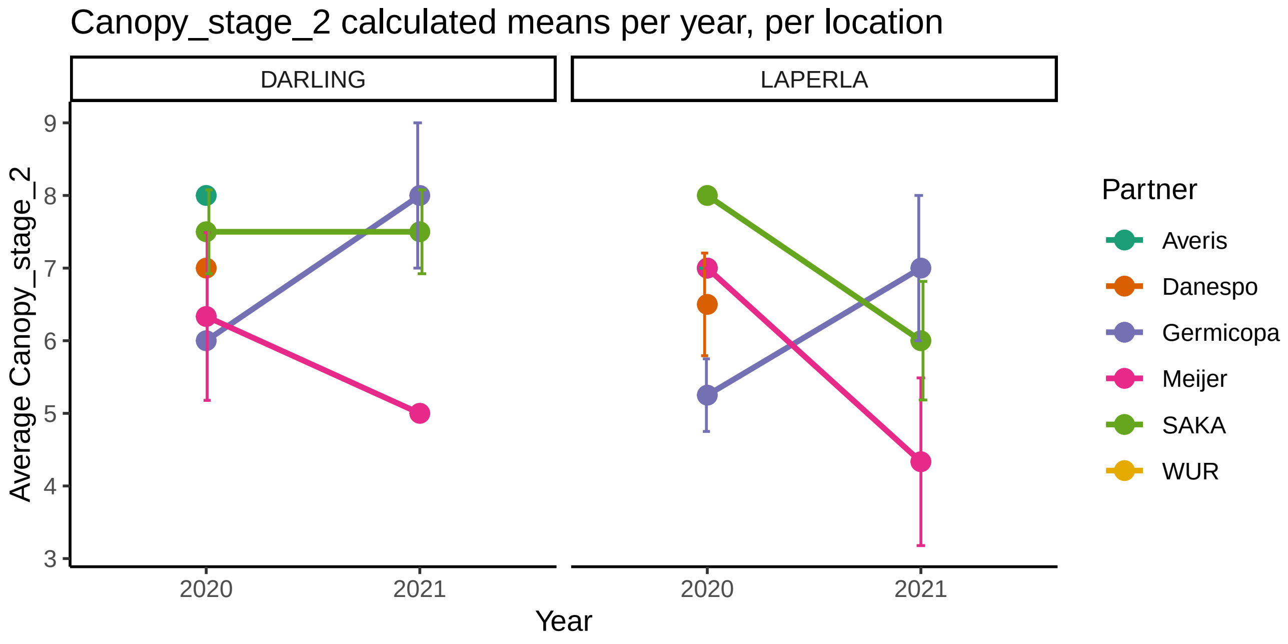

Canopy_stage_2 calculated means per year, per location
DARLING
LAPERLA
Average Canopy_stage_2
Year
2020
2021
Partner
Averis
Danespo
Germicopa
Meijer
SAKA
WUR

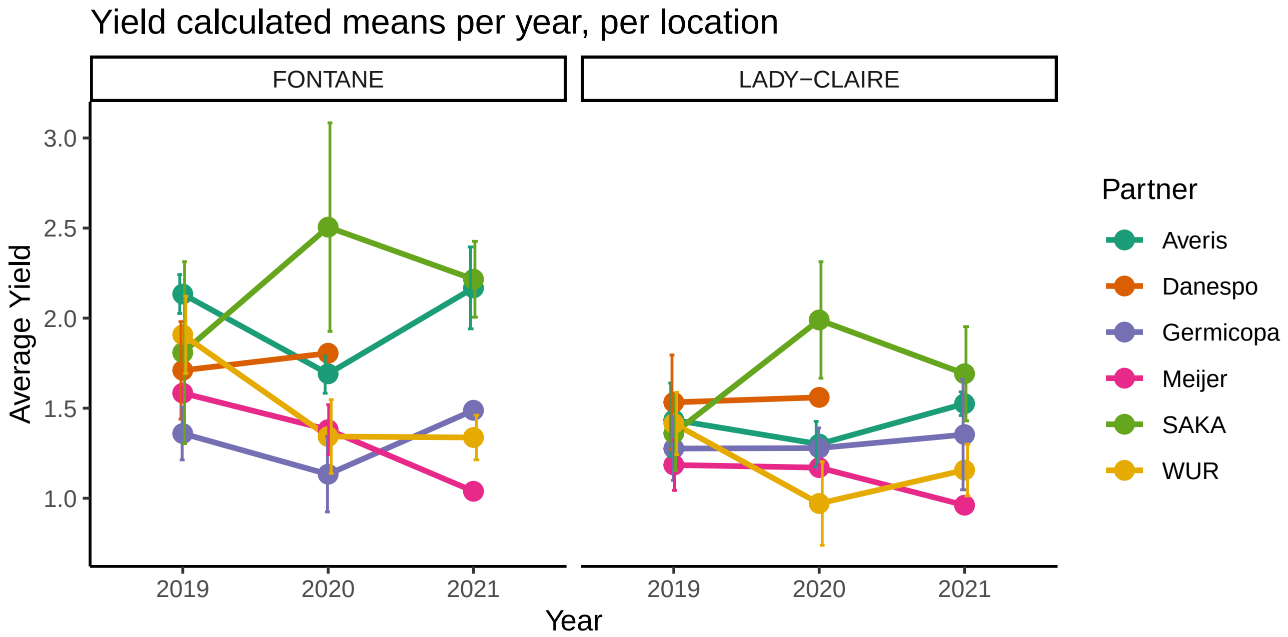
Yield calculated means per year, per location
FONTANE
LADY−CLAIRE
Average Yield
3.0
2.5
2.0
1.5
1.0
2019
2020
2021
Year
Partner
Averis
Danespo
Germicopa
Meijer
SAKA
WUR

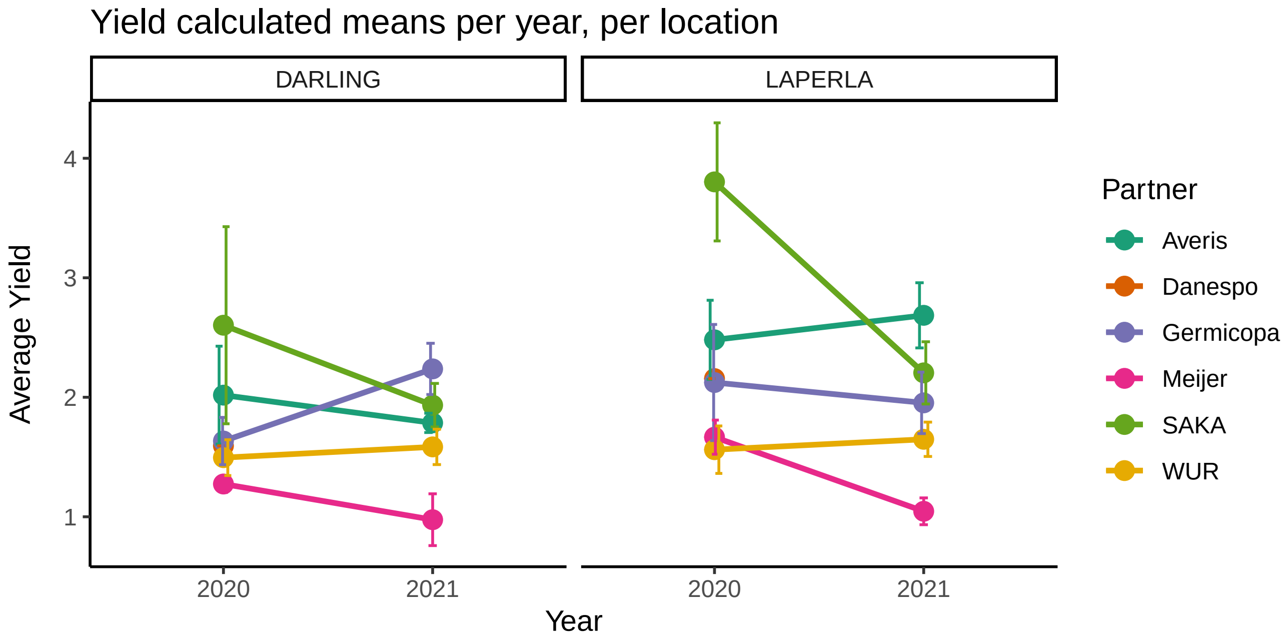
Yield calculated means per year, per location
DARLING
LAPERLA
Average Yield
4
3
2
1
2020
2021
Year
Partner
Averis
Danespo
Germicopa
Meijer
SAKA
WUR

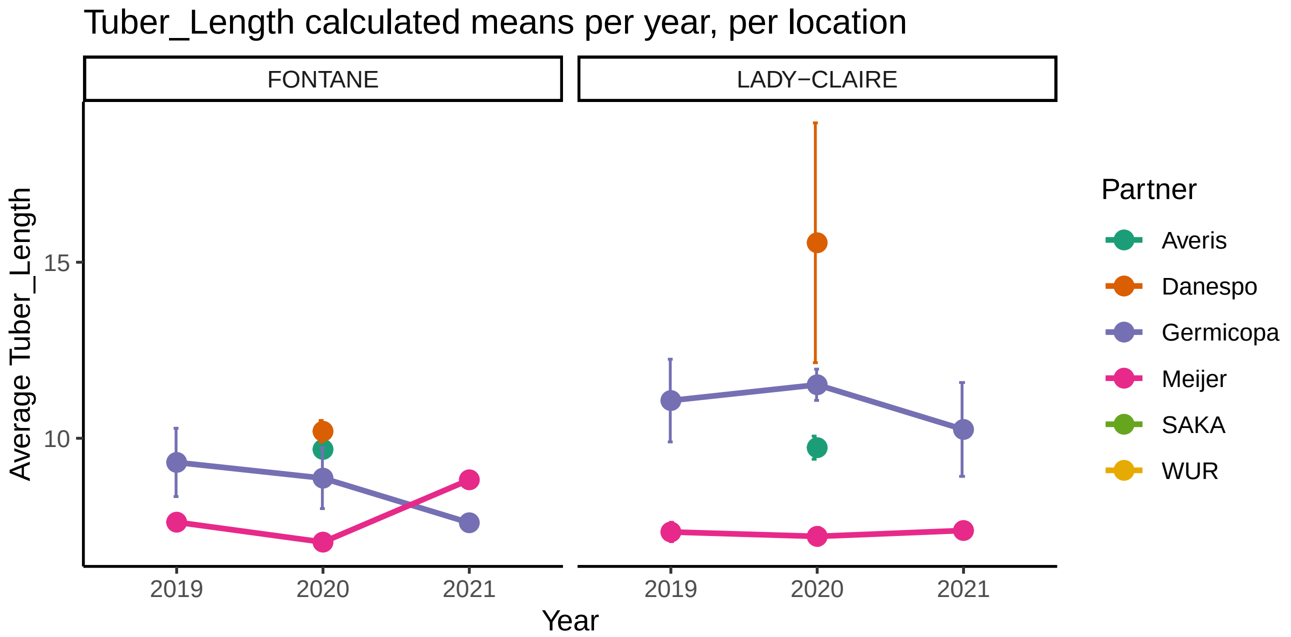
Tuber_Length calculated means per year, per location
FONTANE
LADY−CLAIRE
Average Tuber_Length
15
10
2019
2020
2021
Year
Partner
Averis
Danespo
Germicopa
Meijer
SAKA
WUR

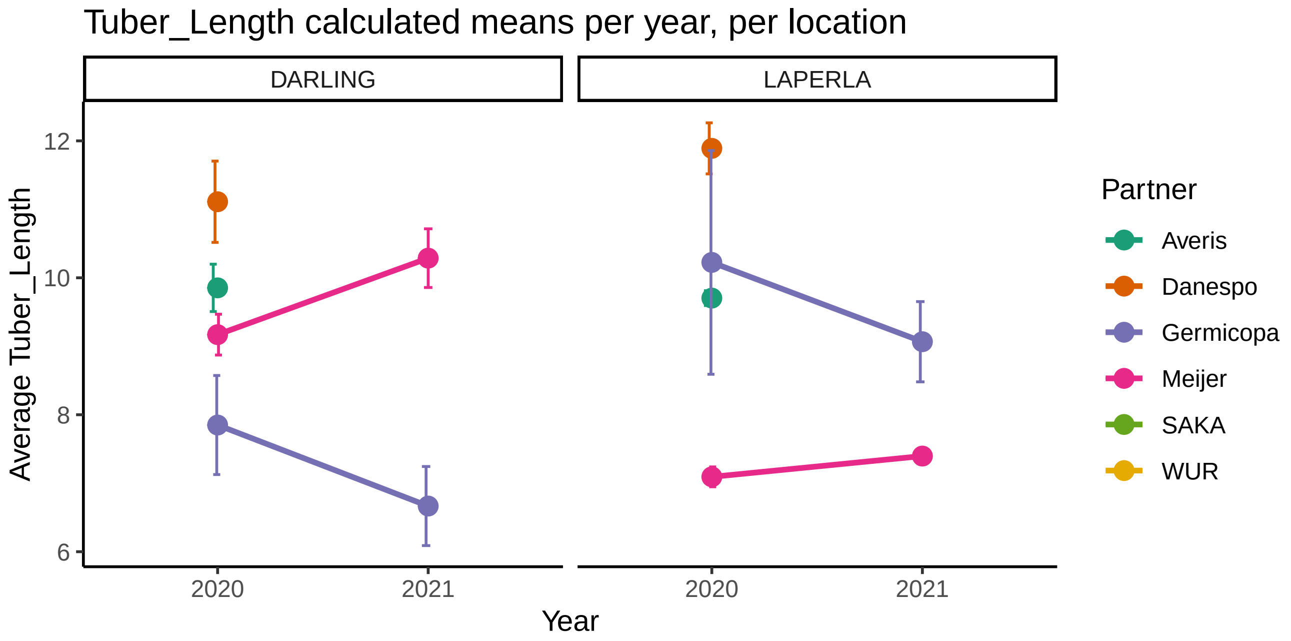
Tuber_Length calculated means per year, per location
DARLING
LAPERLA
Average Tuber_Length
12
10
8
6
2020
2021
Year
Partner
Averis
Danespo
Germicopa
Meijer
SAKA
WUR

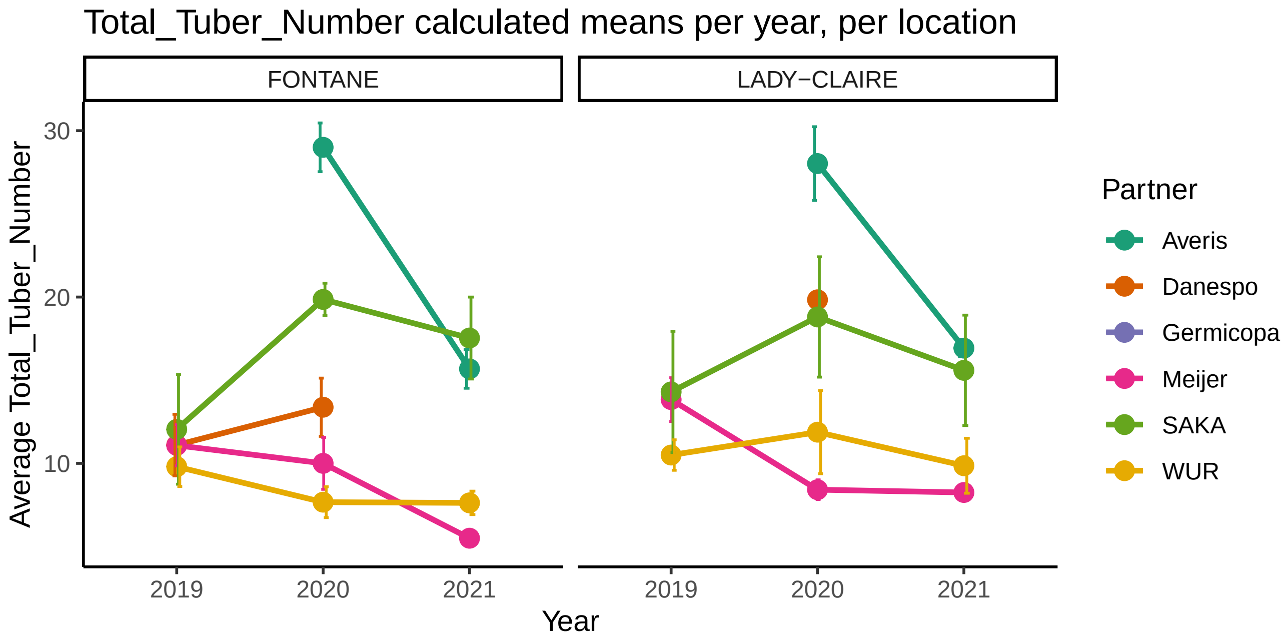

Total_Tuber_Number calculated means per year, per location
FONTANE
LADY−CLAIRE
Average Total_Tuber_Number
30
20
10
2019
2020
2021
Year
Partner
Averis
Danespo
Germicopa
Meijer
SAKA
WUR

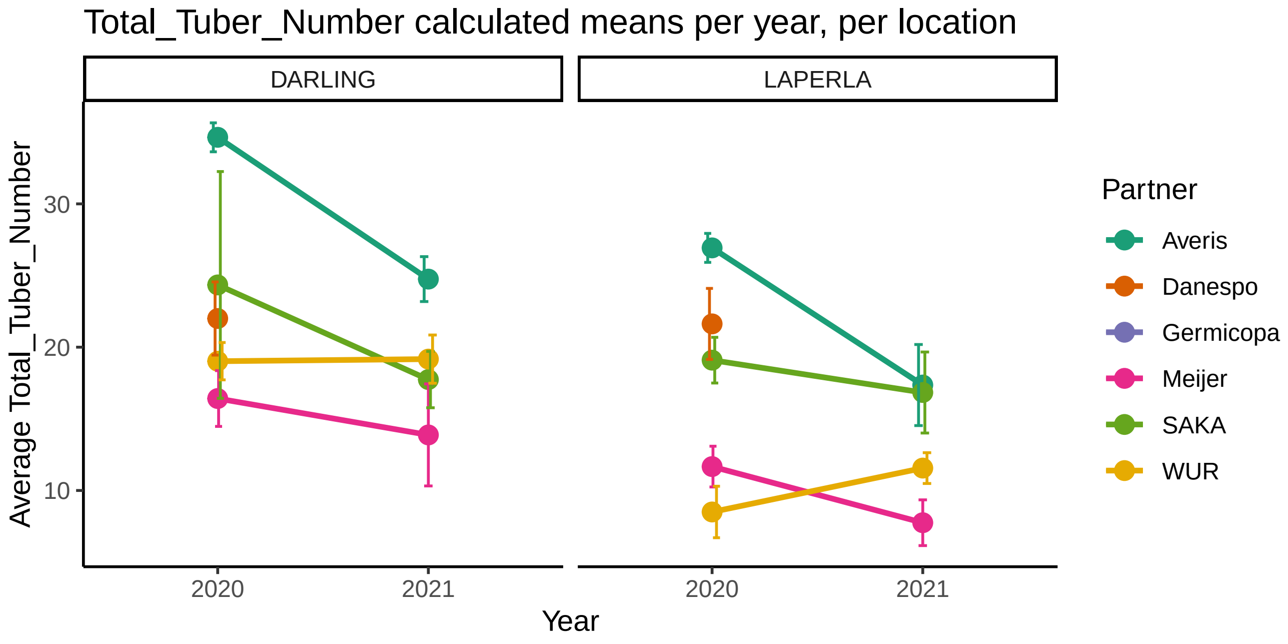

Total_Tuber_Number calculated means per year, per location
DARLING
LAPERLA
Average Total_Tuber_Number
30
20
10
2020
2021
Year
Partner
Averis
Danespo
Germicopa
Meijer
SAKA
WUR

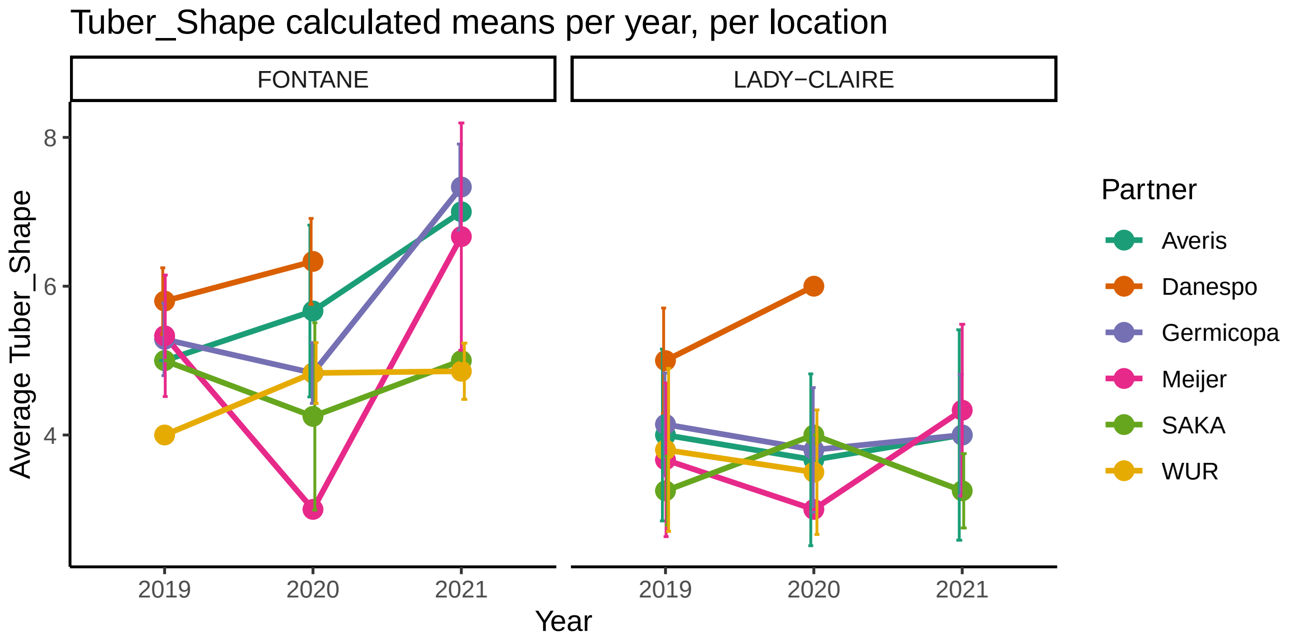
Tuber_Shape calculated means per year, per location
FONTANE
LADY−CLAIRE
Average Tuber_Shape
8
6
4
2019
2020
2021
Year
Partner
Averis
Danespo
Germicopa
Meijer
SAKA
WUR

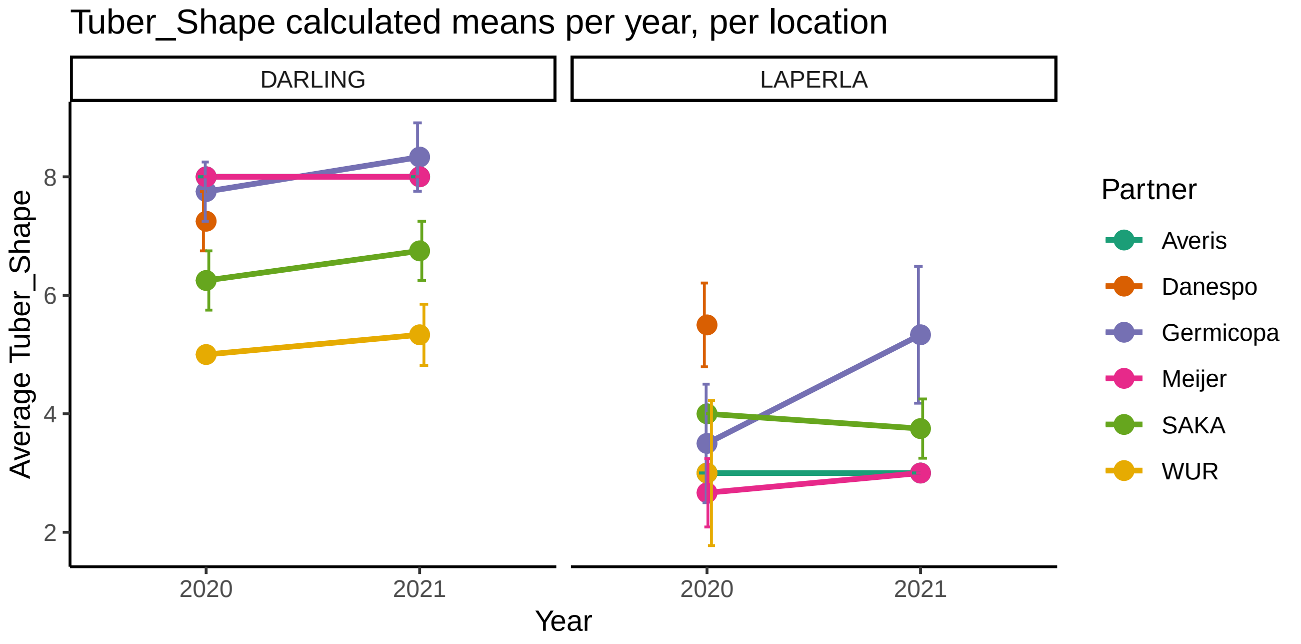
Tuber_Shape calculated means per year, per location
DARLING
LAPERLA
Average Tuber_Shape
8
6
4
2
2020
2021
Year
Partner
Averis
Danespo
Germicopa
Meijer
SAKA
WUR

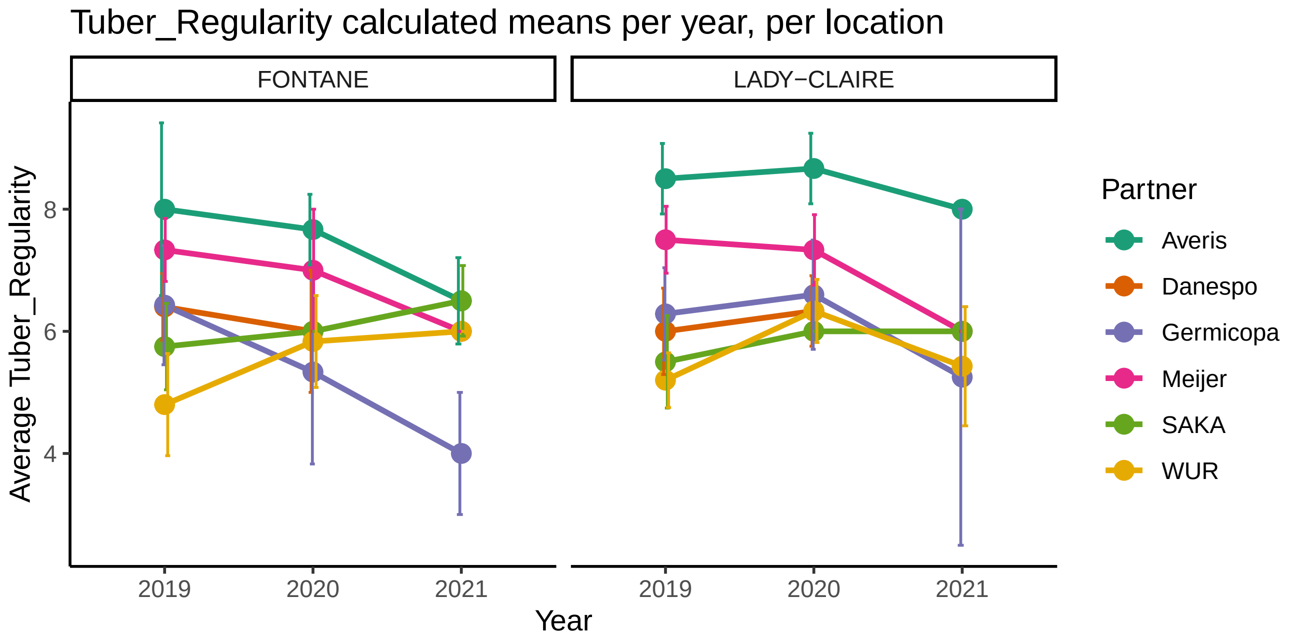

Tuber_Regularity calculated means per year, per location
FONTANE
LADY−CLAIRE
Average Tuber_Regularity
8
6
4
2019
2020
2021
Year
Partner
Averis
Danespo
Germicopa
Meijer
SAKA
WUR

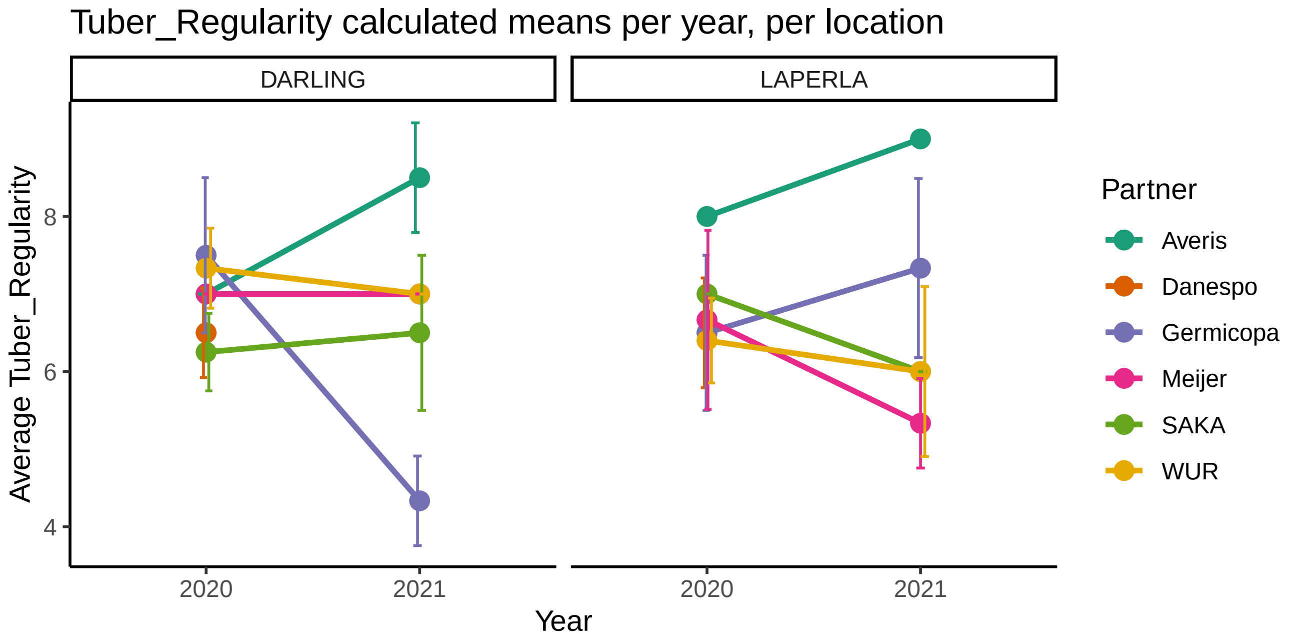

Tuber_Regularity calculated means per year, per location
DARLING
LAPERLA
Average Tuber_Regularity
8
6
4
2020
2021
Year
Partner
Averis
Danespo
Germicopa
Meijer
SAKA
WUR

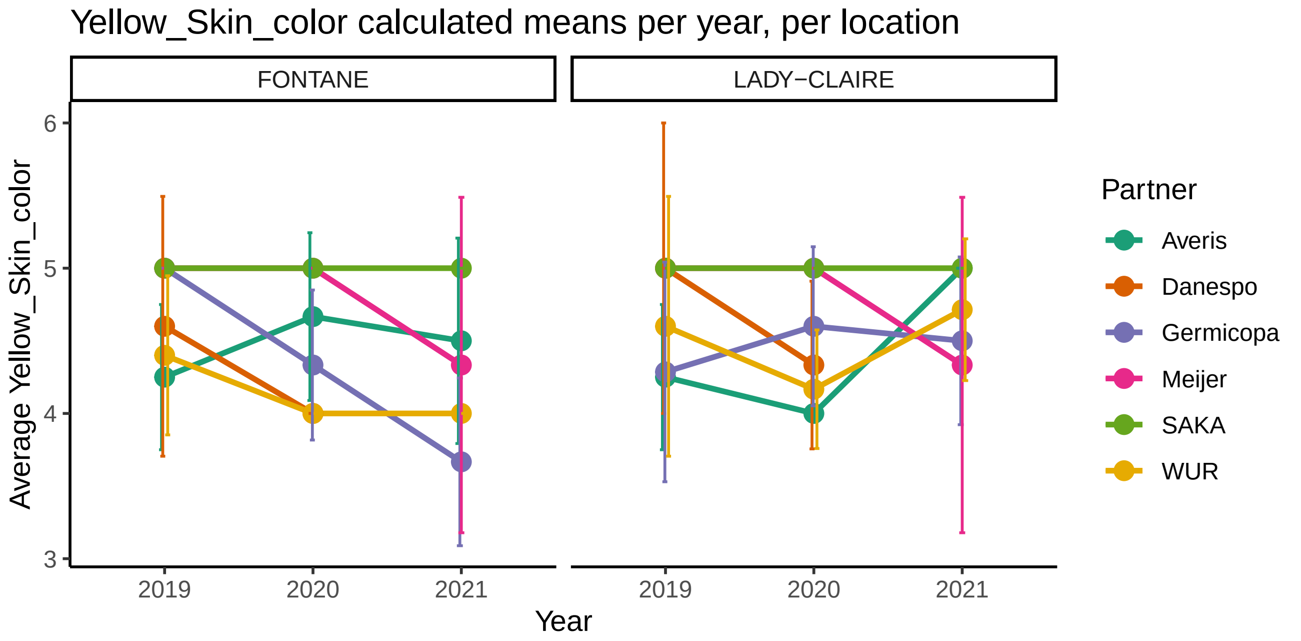
Yellow_Skin_color calculated means per year, per location
FONTANE
LADY−CLAIRE
Average Yellow_Skin_color
Year
Partner
Averis
Danespo
Germicopa
Meijer
SAKA
WUR

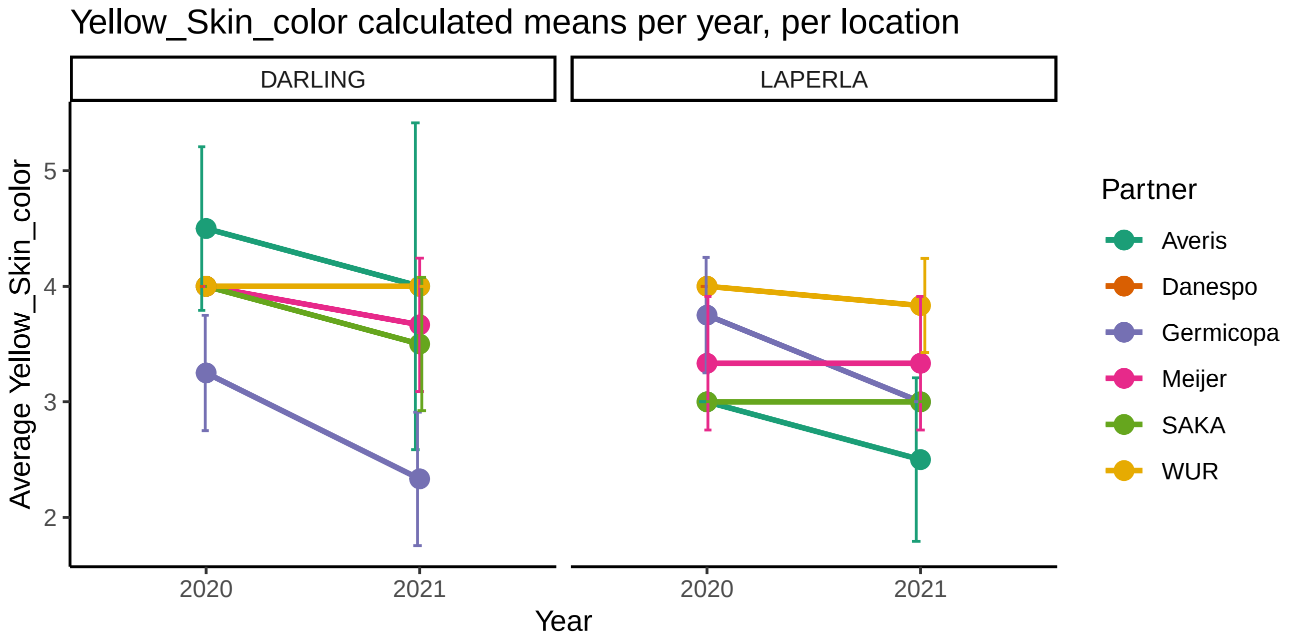
Yellow_Skin_color calculated means per year, per location
DARLING
LAPERLA
Average Yellow_Skin_color
Year
Partner
Averis
Danespo
Germicopa
Meijer
SAKA
WUR

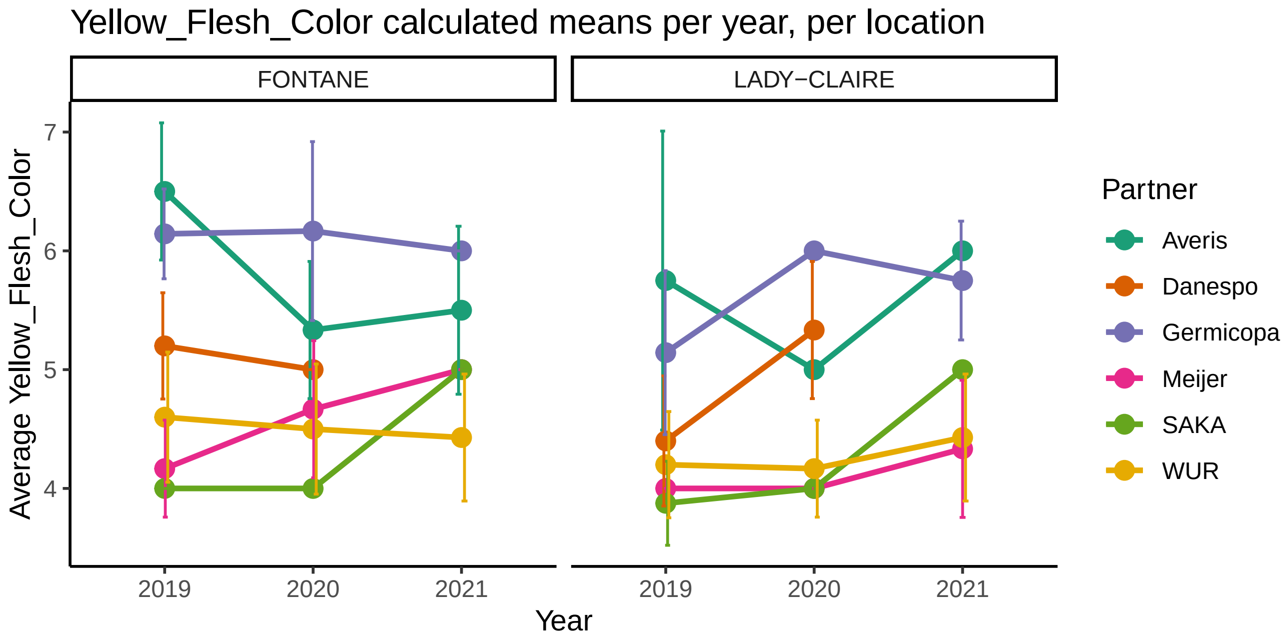

Yellow_Flesh_Color calculated means per year, per location
FONTANE
LADY−CLAIRE
Average Yellow_Flesh_Color
7
6
5
4
2019
2020
2021
Year
Partner
Averis
Danespo
Germicopa
Meijer
SAKA
WUR

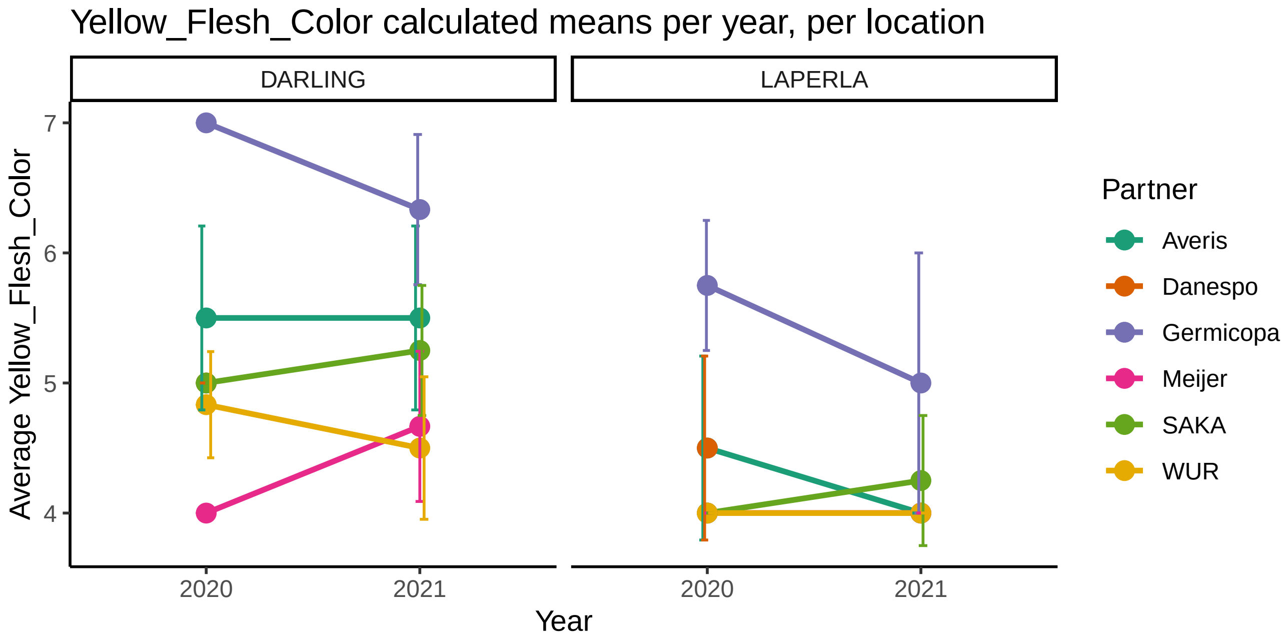

Yellow_Flesh_Color calculated means per year, per location
DARLING
LAPERLA
Average Yellow_Flesh_Color
7
6
5
4
2020
2021
Year
Partner
Averis
Danespo
Germicopa
Meijer
SAKA
WUR

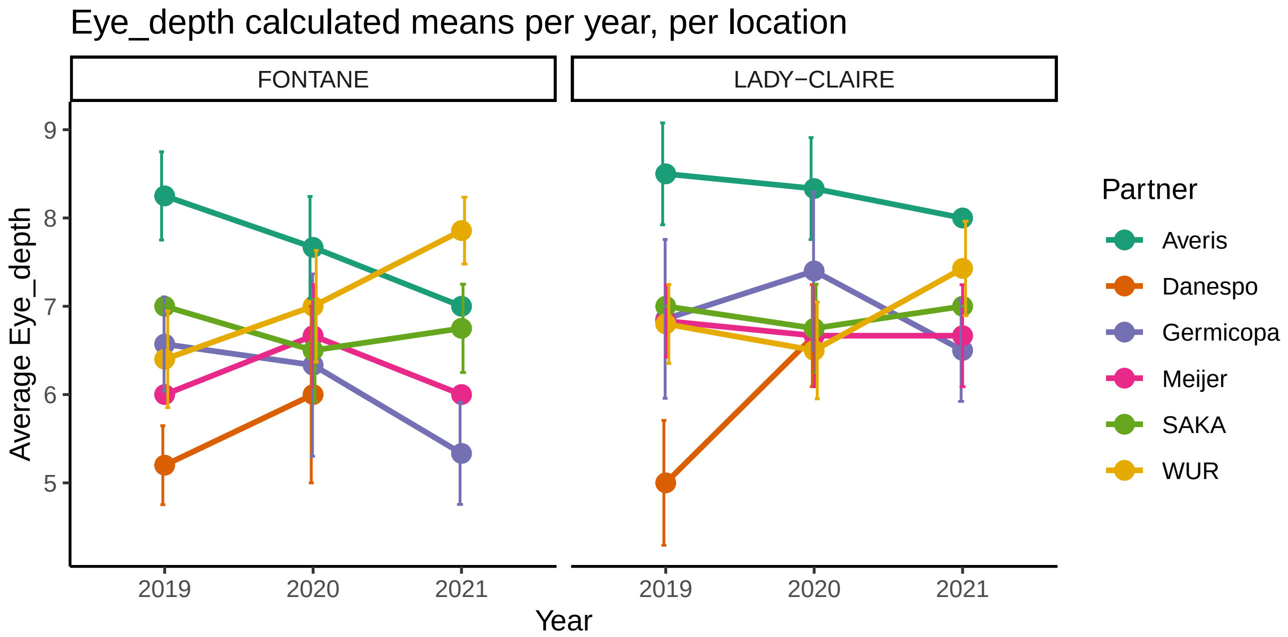
Eye_depth calculated means per year, per location
FONTANE
LADY−CLAIRE
Average Eye_depth
9
8
7
6
5
2019
2020
2021
Year
Partner
Averis
Danespo
Germicopa
Meijer
SAKA
WUR

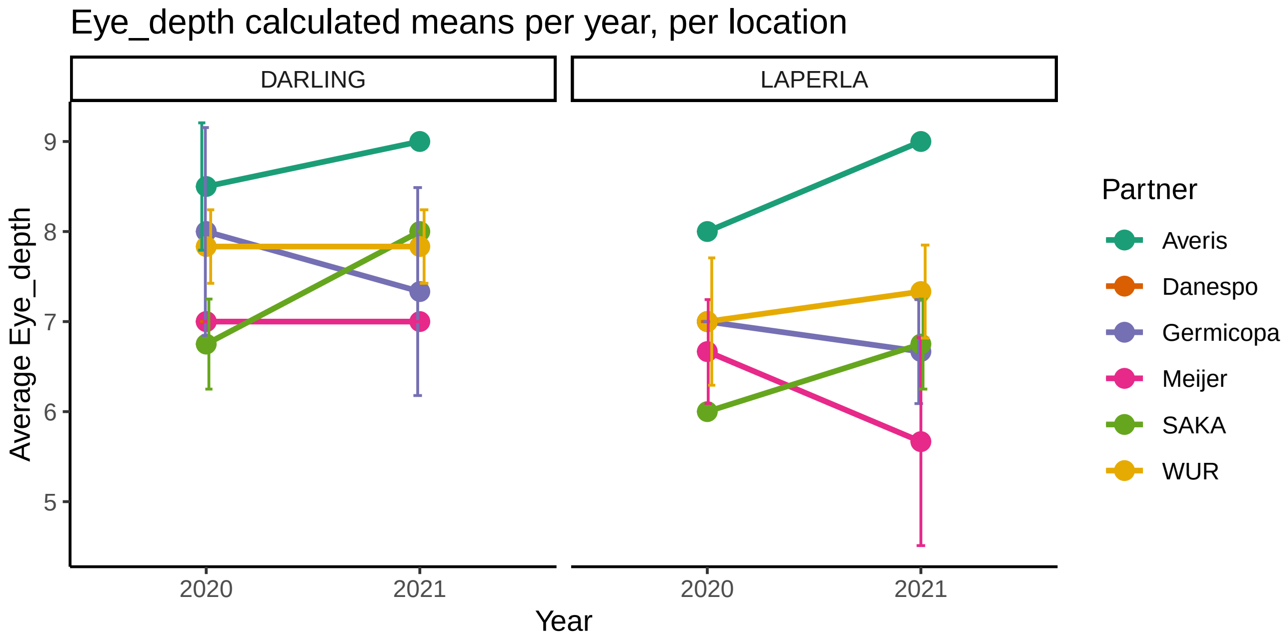
Eye_depth calculated means per year, per location
DARLING
LAPERLA
Average Eye_depth
9
8
7
6
5
2020
2021
Year
Partner
Averis
Danespo
Germicopa
Meijer
SAKA
WUR

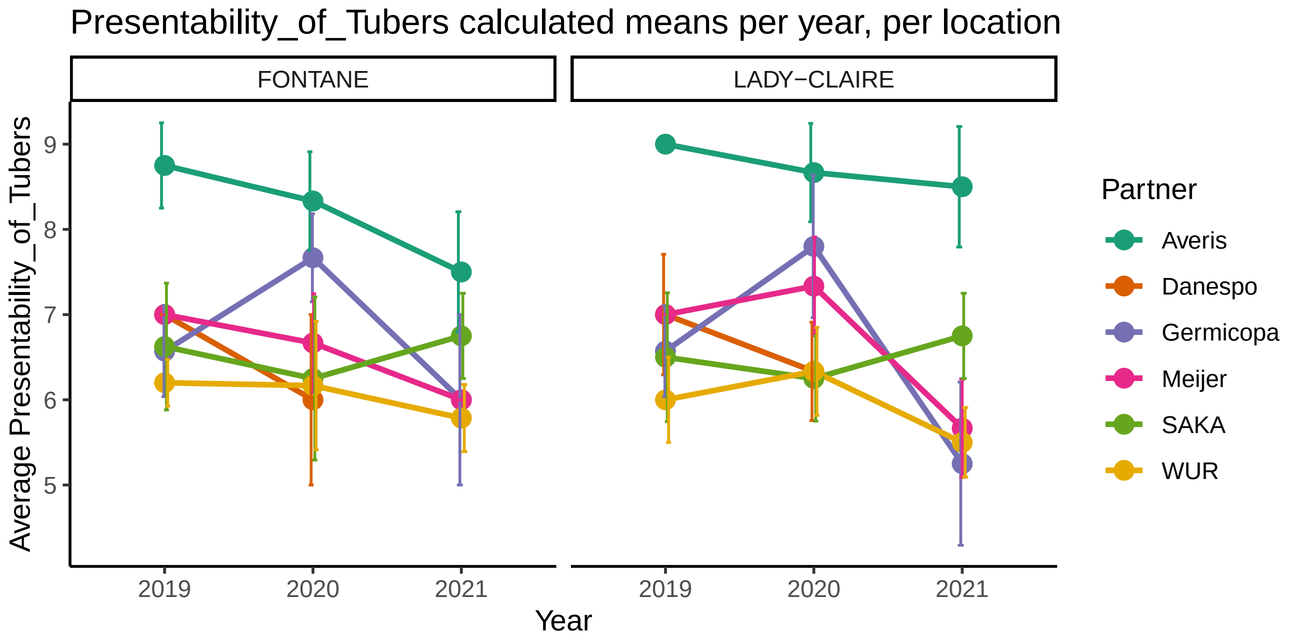

Presentability_of_Tubers calculated means per year, per location
FONTANE
LADY−CLAIRE
Average Presentability_of_Tubers
9
8
7
6
5
2019
2020
2021
Year
Partner
Averis
Danespo
Germicopa
Meijer
SAKA
WUR

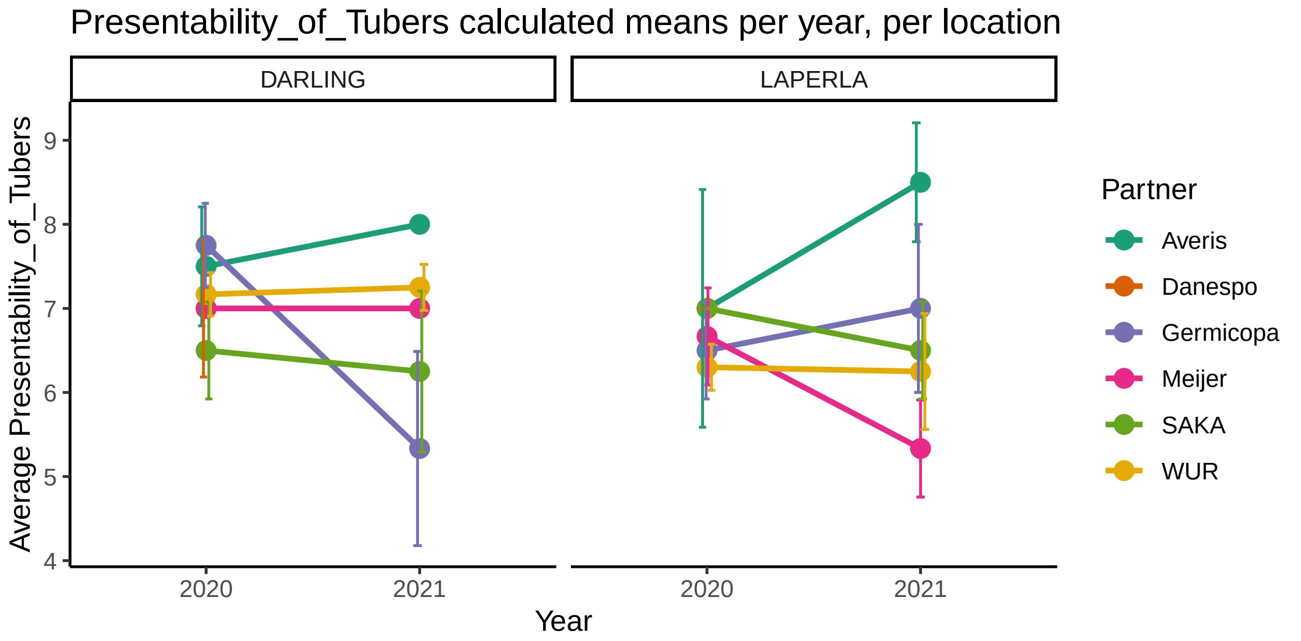

Presentability_of_Tubers calculated means per year, per location
DARLING
LAPERLA
Average Presentability_of_Tubers
9
8
7
6
5
4
2020
2021
Year
Partner
Averis
Danespo
Germicopa
Meijer
SAKA
WUR

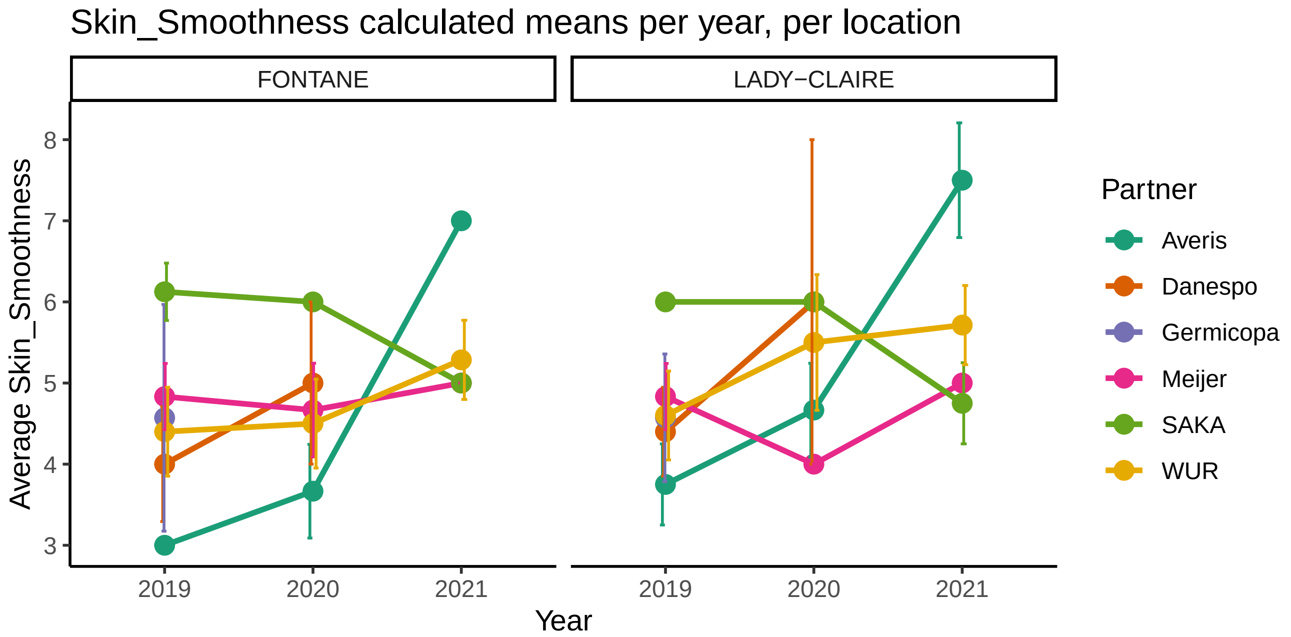
Skin_Smoothness calculated means per year, per location
FONTANE
LADY−CLAIRE
Average Skin_Smoothness
3
4
5
6
7
8
2019
2020
2021
2019
2020
2021
Year
Partner
Averis
Danespo
Germicopa
Meijer
SAKA
WUR

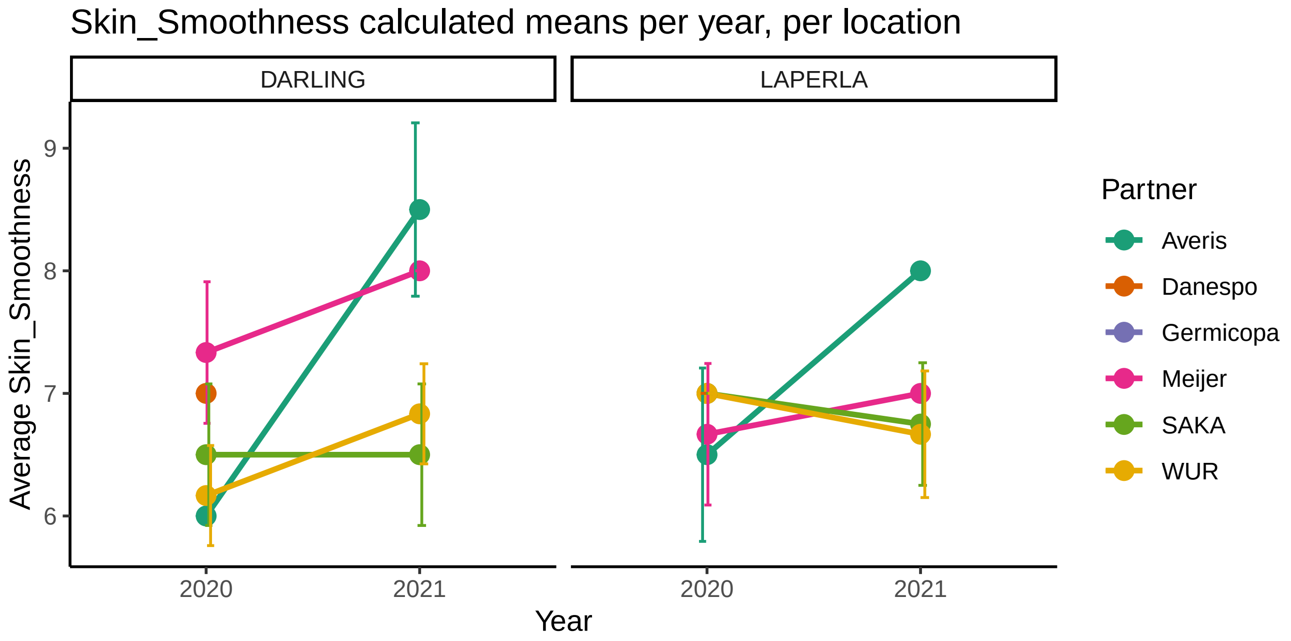
Skin_Smoothness calculated means per year, per location
DARLING
LAPERLA
Average Skin_Smoothness
6
7
8
9
2020
2021
2020
2021
Year
Partner
Averis
Danespo
Germicopa
Meijer
SAKA
WUR

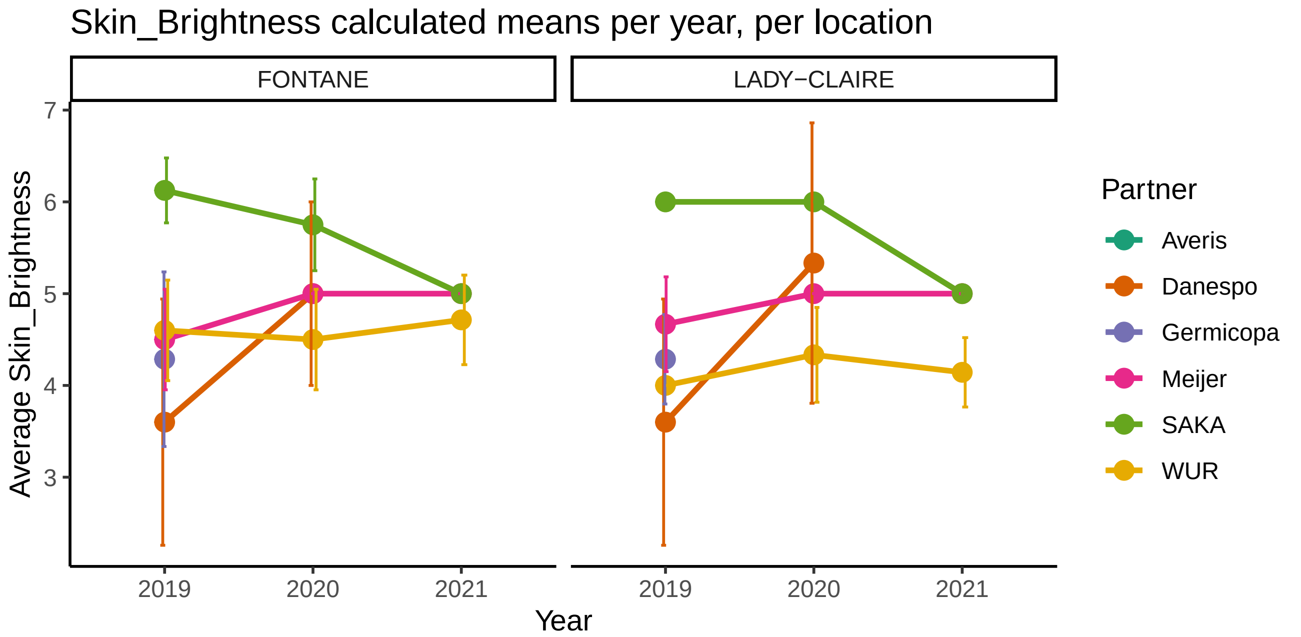
Skin_Brightness calculated means per year, per location
FONTANE
LADY−CLAIRE
Average Skin_Brightness
7
6
5
4
3
2019
2020
2021
Year
Partner
Averis
Danespo
Germicopa
Meijer
SAKA
WUR

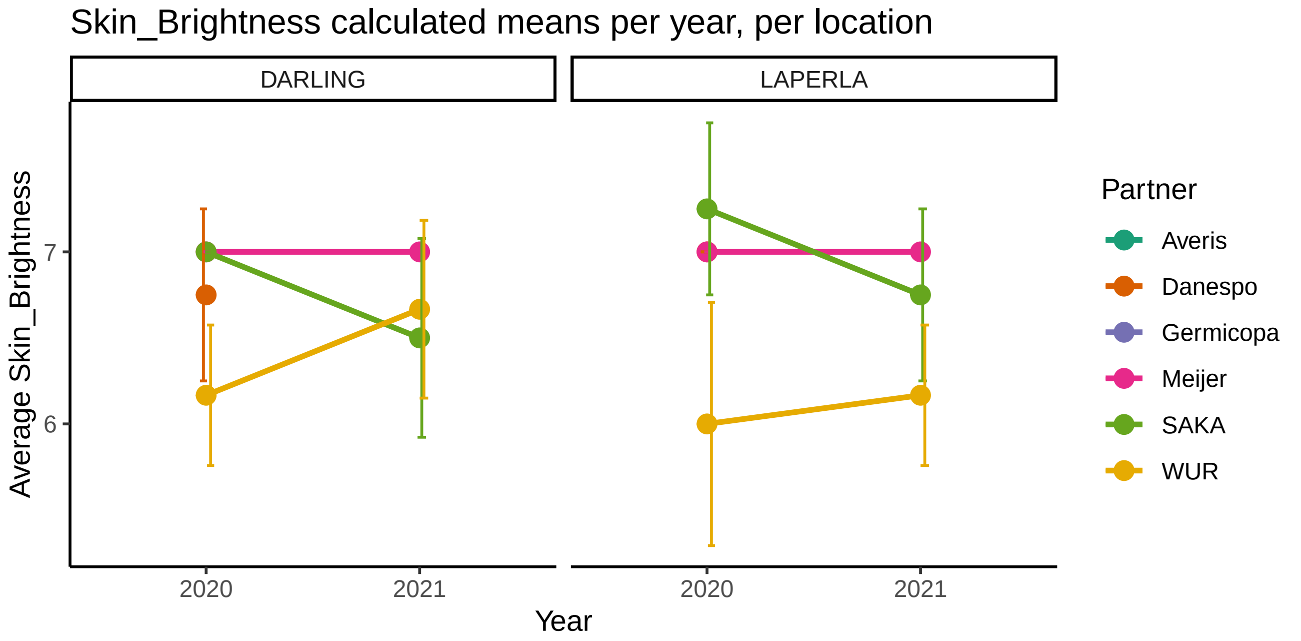
Skin_Brightness calculated means per year, per location
DARLING
LAPERLA
Average Skin_Brightness
7
6
2020
2021
Year
Partner
Averis
Danespo
Germicopa
Meijer
SAKA
WUR

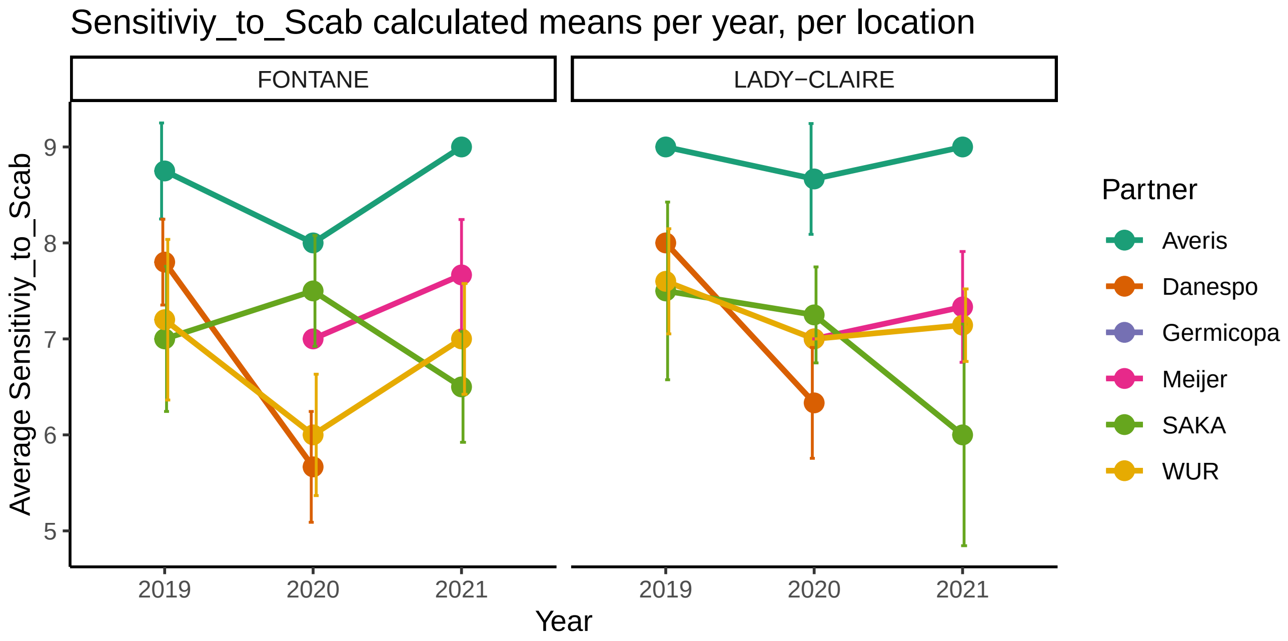
Sensitiviy_to_Scab calculated means per year, per location
FONTANE
LADY−CLAIRE
Average Sensitiviy_to_Scab
9
8
7
6
5
2019
2020
2021
Year
Partner
Averis
Danespo
Germicopa
Meijer
SAKA
WUR

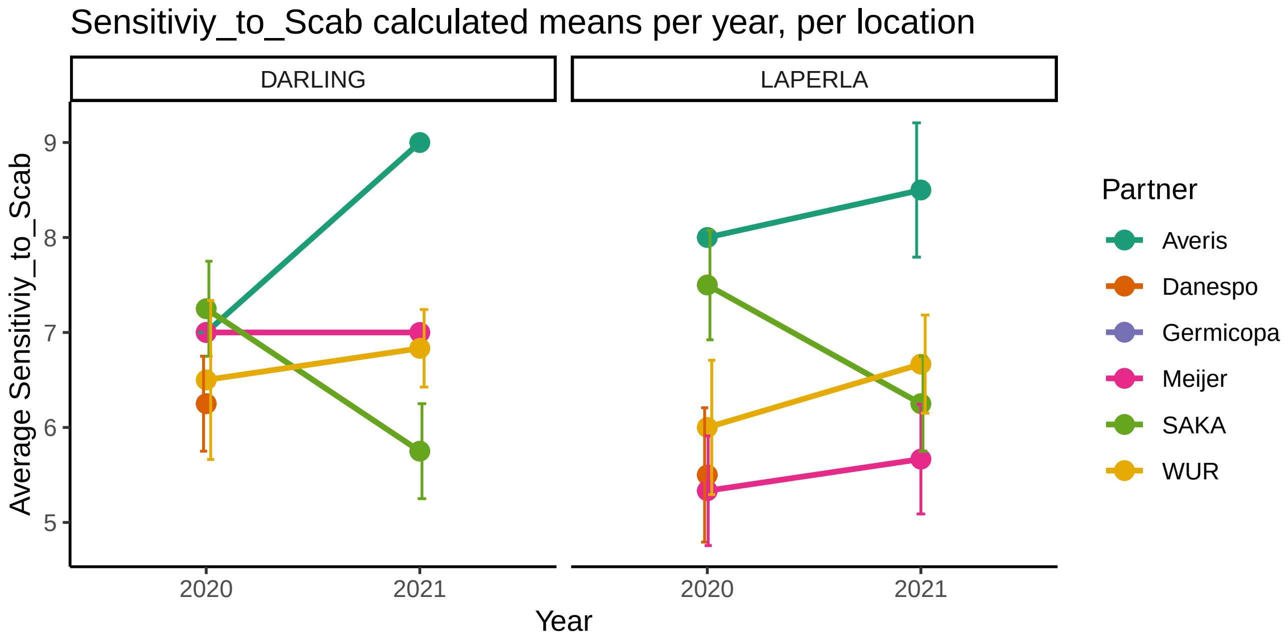
Sensitiviy_to_Scab calculated means per year, per location
DARLING
LAPERLA
Average Sensitiviy_to_Scab
9
8
7
6
5
2020
2021
Year
Partner
Averis
Danespo
Germicopa
Meijer
SAKA
WUR

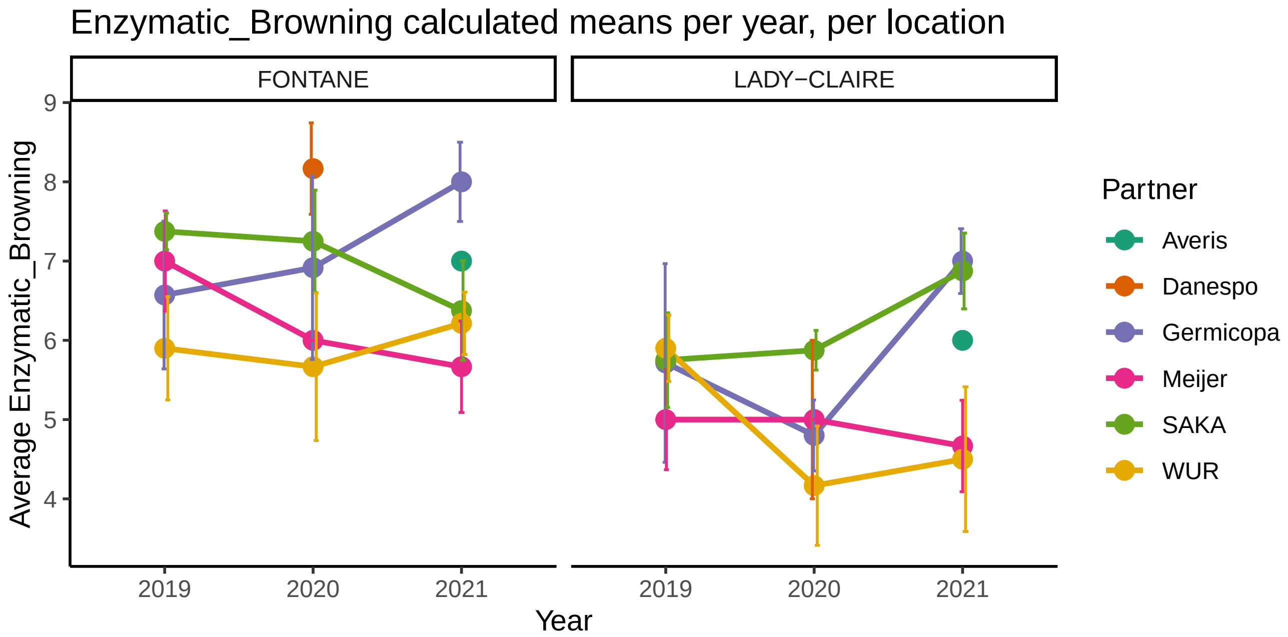
Enzymatic_Browning calculated means per year, per location
FONTANE
LADY−CLAIRE
Average Enzymatic_Browning
9
8
7
6
5
4
2019
2020
2021
Year
Partner
Averis
Danespo
Germicopa
Meijer
SAKA
WUR

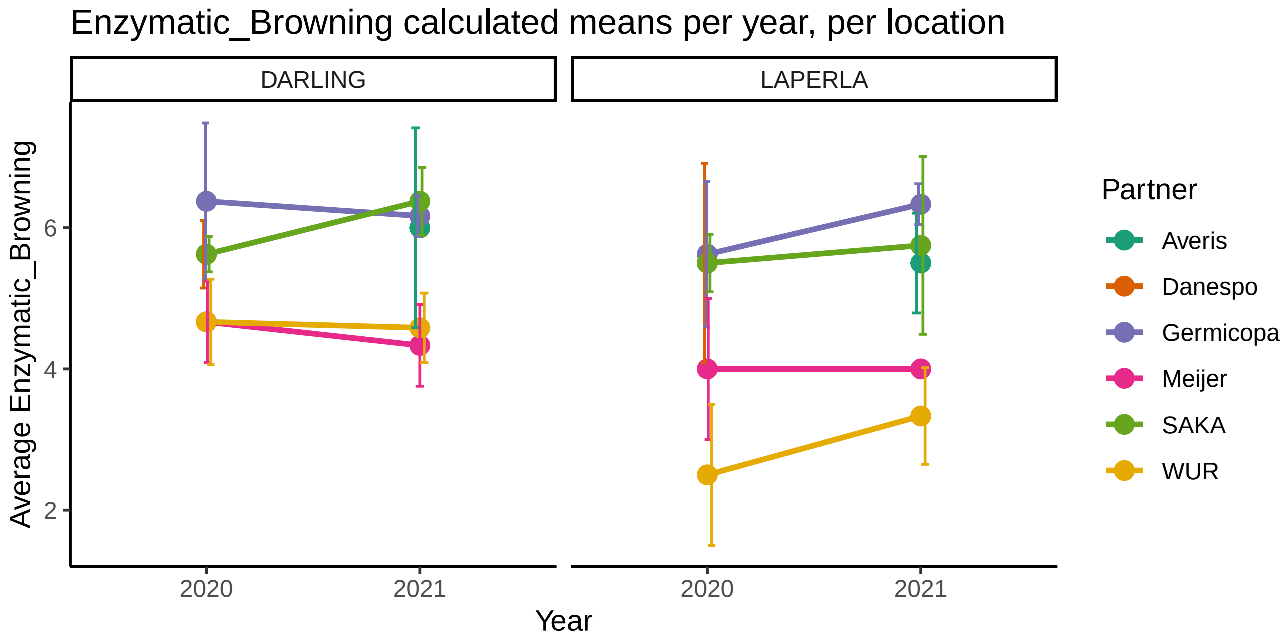
Enzymatic_Browning calculated means per year, per location
DARLING
LAPERLA
Average Enzymatic_Browning
6
4
2
2020
2021
Year
Partner
Averis
Danespo
Germicopa
Meijer
SAKA
WUR

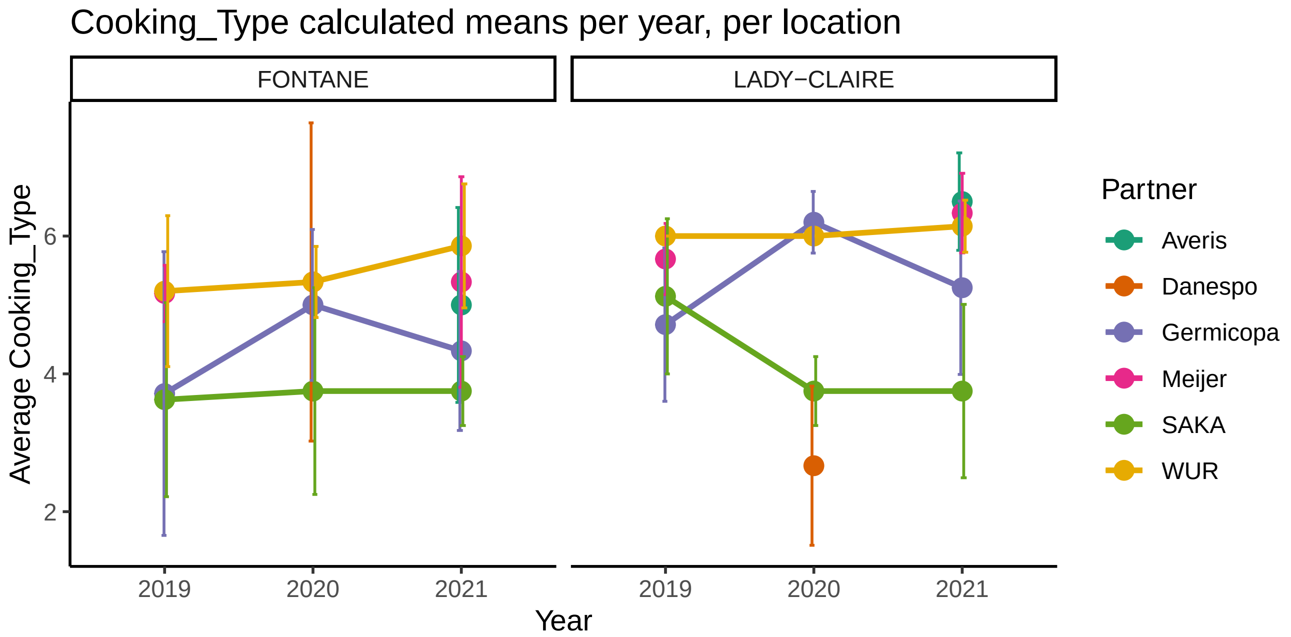
Cooking_Type calculated means per year, per location
FONTANE
LADY−CLAIRE
Average Cooking_Type
2
4
6
2019
2020
2021
Year
Partner
Averis
Danespo
Germicopa
Meijer
SAKA
WUR

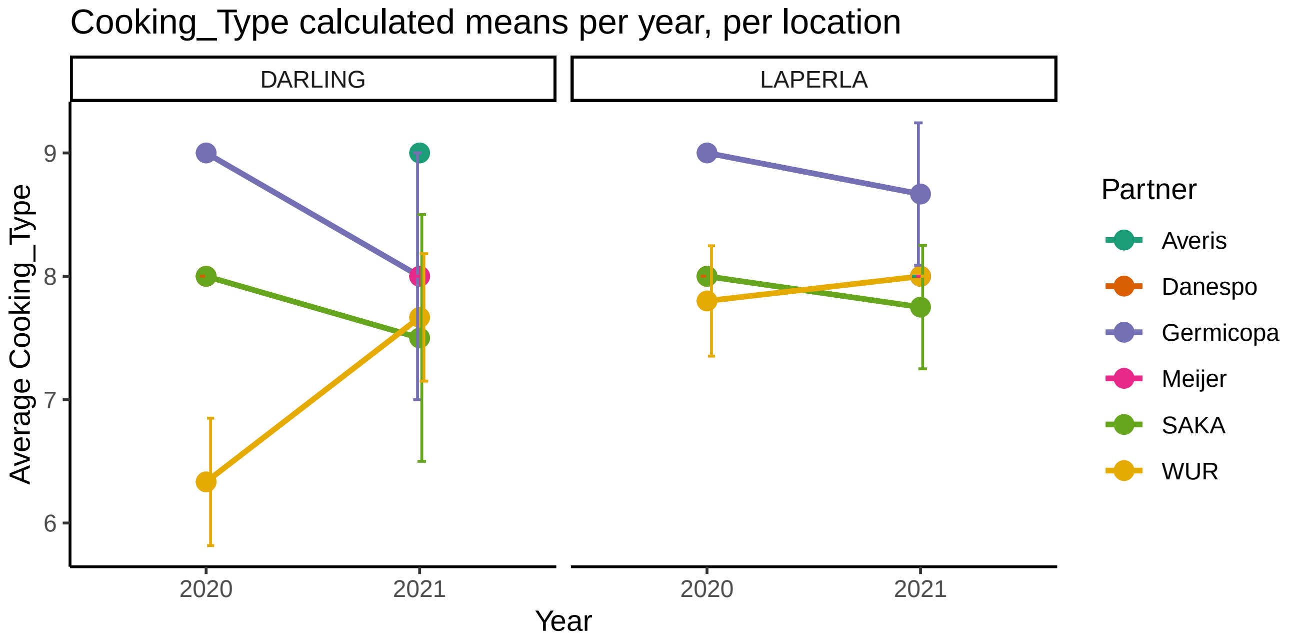
Cooking_Type calculated means per year, per location
DARLING
LAPERLA
Average Cooking_Type
6
7
8
9
2020
2021
Year
Partner
Averis
Danespo
Germicopa
Meijer
SAKA
WUR

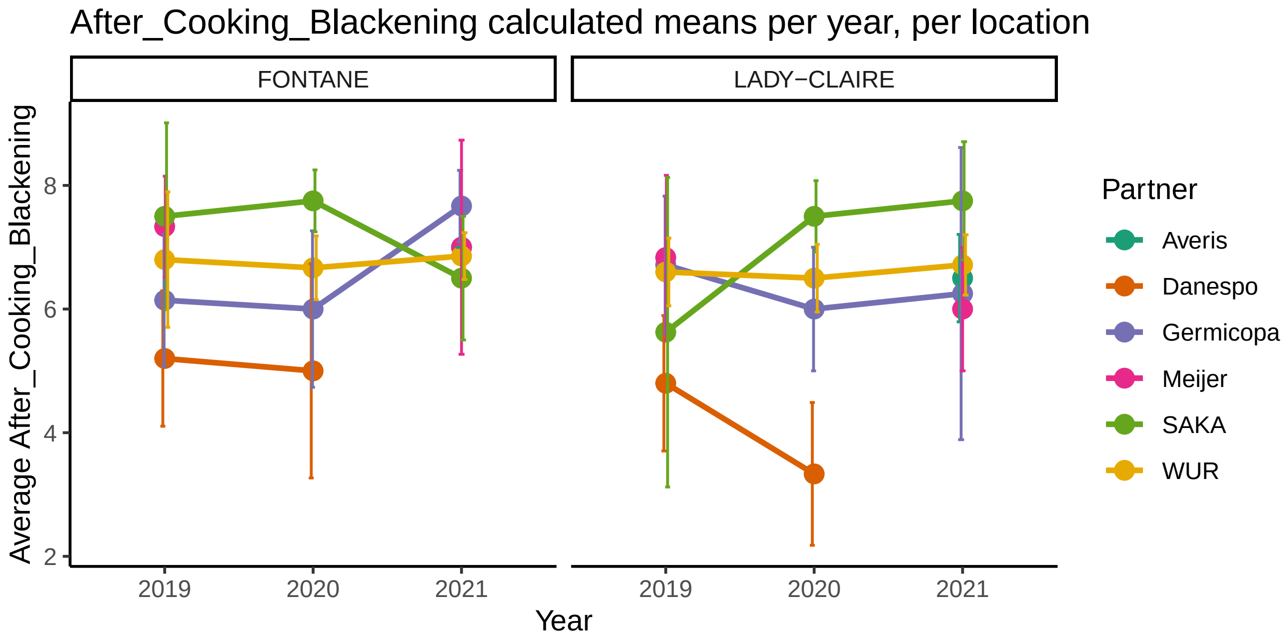
After_Cooking_Blackening calculated means per year, per location
FONTANE
LADY−CLAIRE
Average After_Cooking_Blackening
2
4
6
8
2019
2020
2021
Year
Partner
Averis
Danespo
Germicopa
Meijer
SAKA
WUR

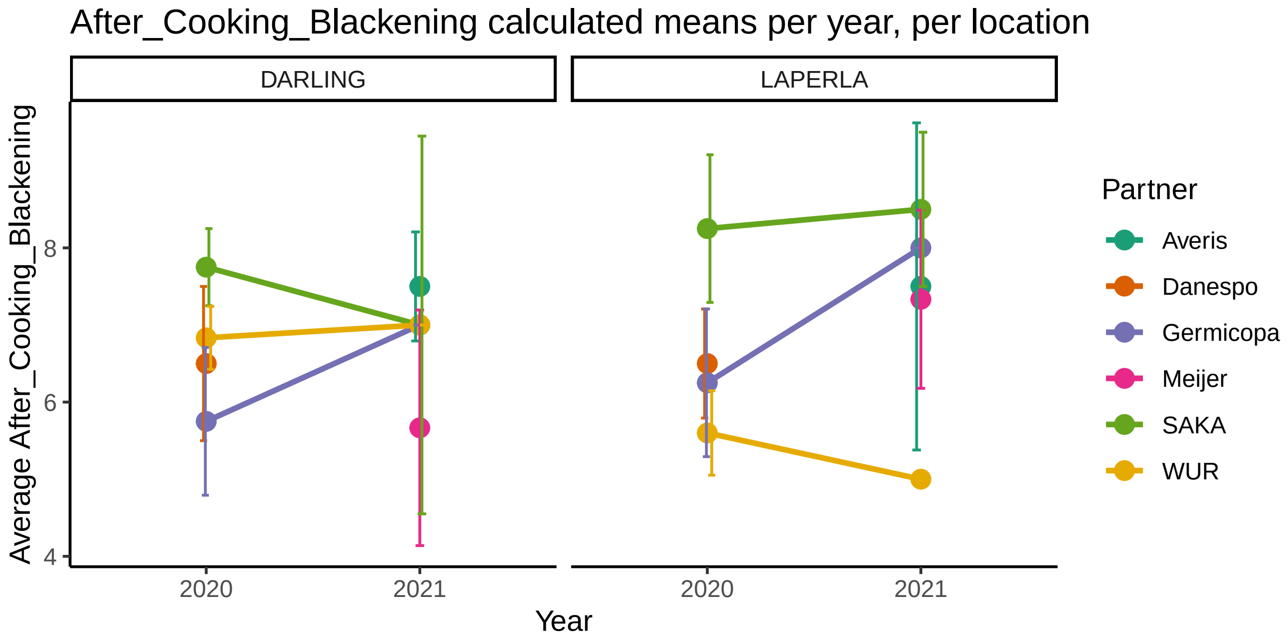
After_Cooking_Blackening calculated means per year, per location
DARLING
LAPERLA
Average After_Cooking_Blackening
4
6
8
2020
2021
Year
Partner
Averis
Danespo
Germicopa
Meijer
SAKA
WUR

Chipping_color_1_8 calculated means per year, per location

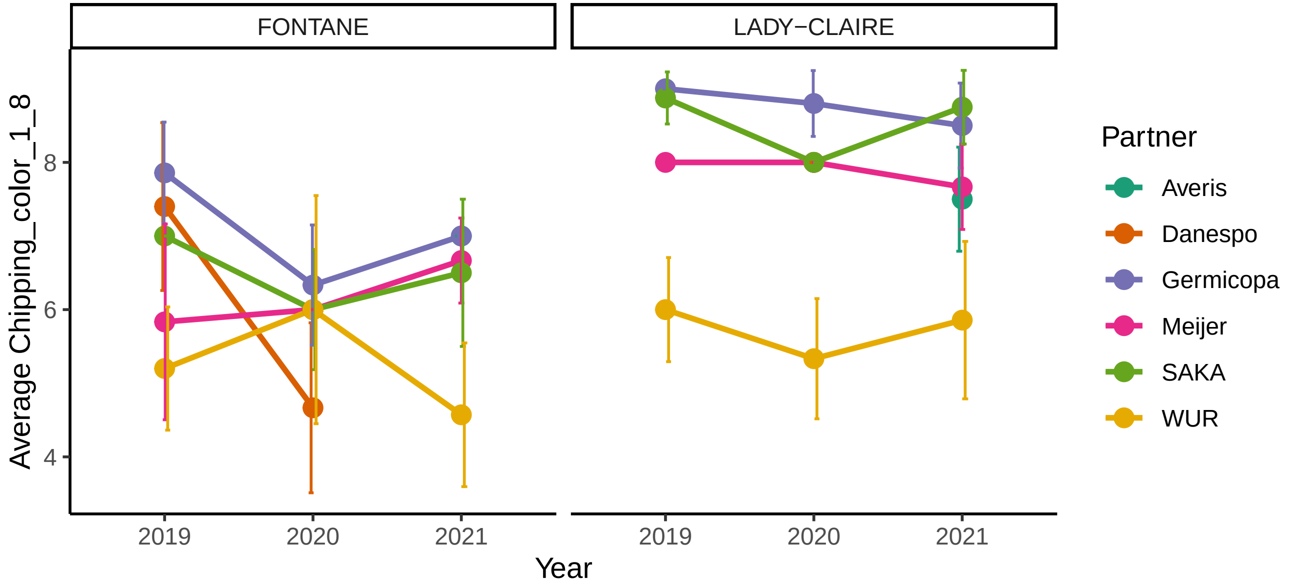

Chipping_color_1_8 calculated means per year, per location

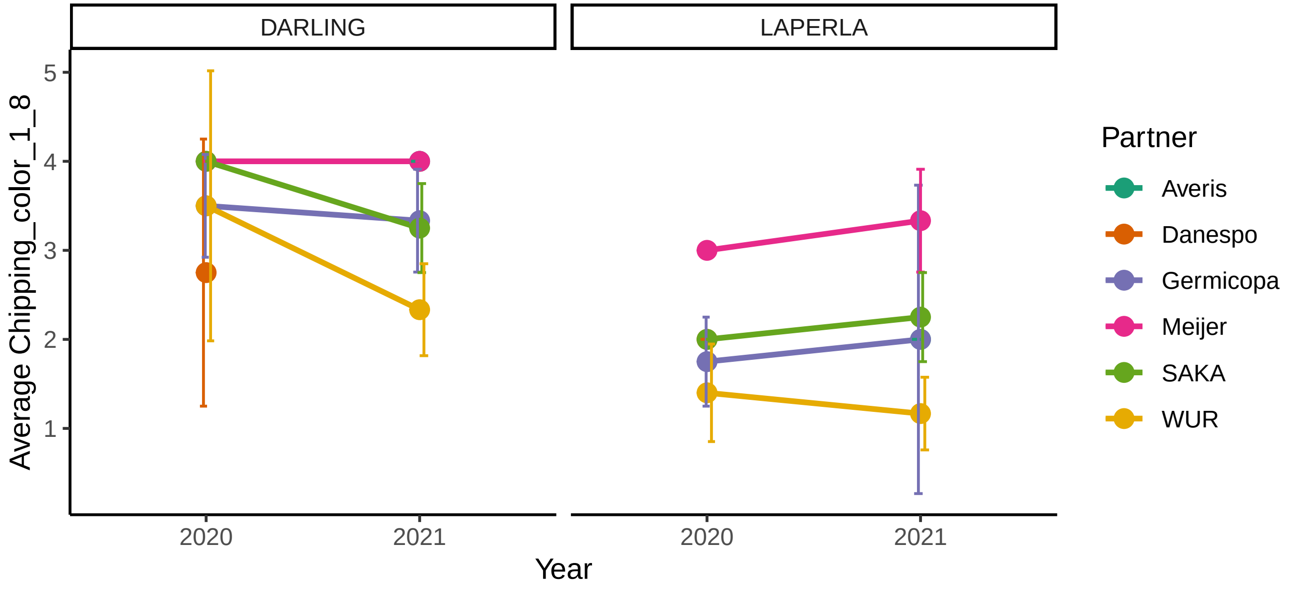

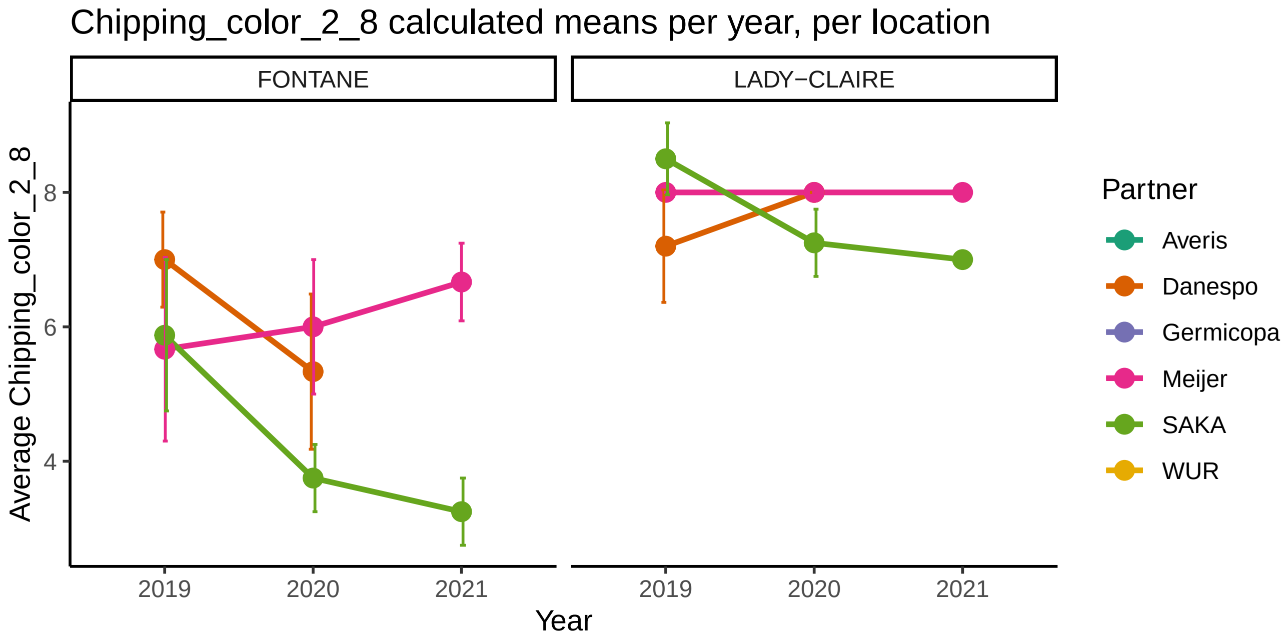
Chipping_color_2_8 calculated means per year, per location
FONTANE
LADY−CLAIRE
Average Chipping_color_2_8
4
6
8
2019
2020
2021
Year
Partner
Averis
Danespo
Germicopa
Meijer
SAKA
WUR

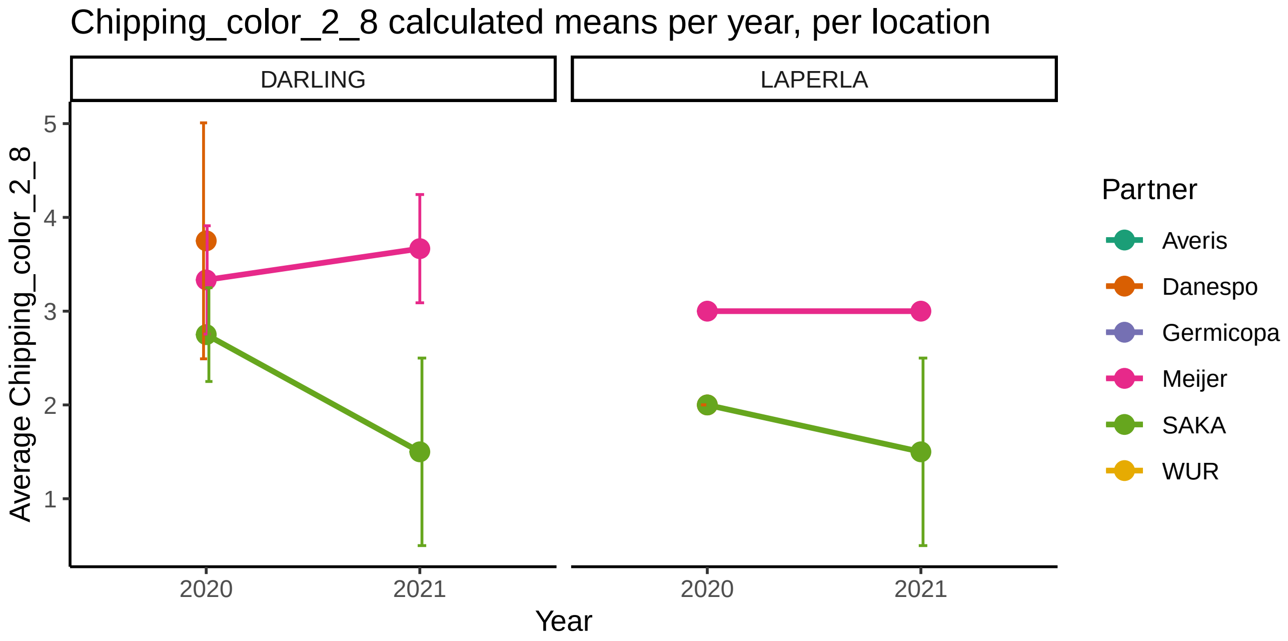
Chipping_color_2_8 calculated means per year, per location
DARLING
LAPERLA
Average Chipping_color_2_8
1
2
3
4
5
2020
2021
Year
Partner
Averis
Danespo
Germicopa
Meijer
SAKA
WUR

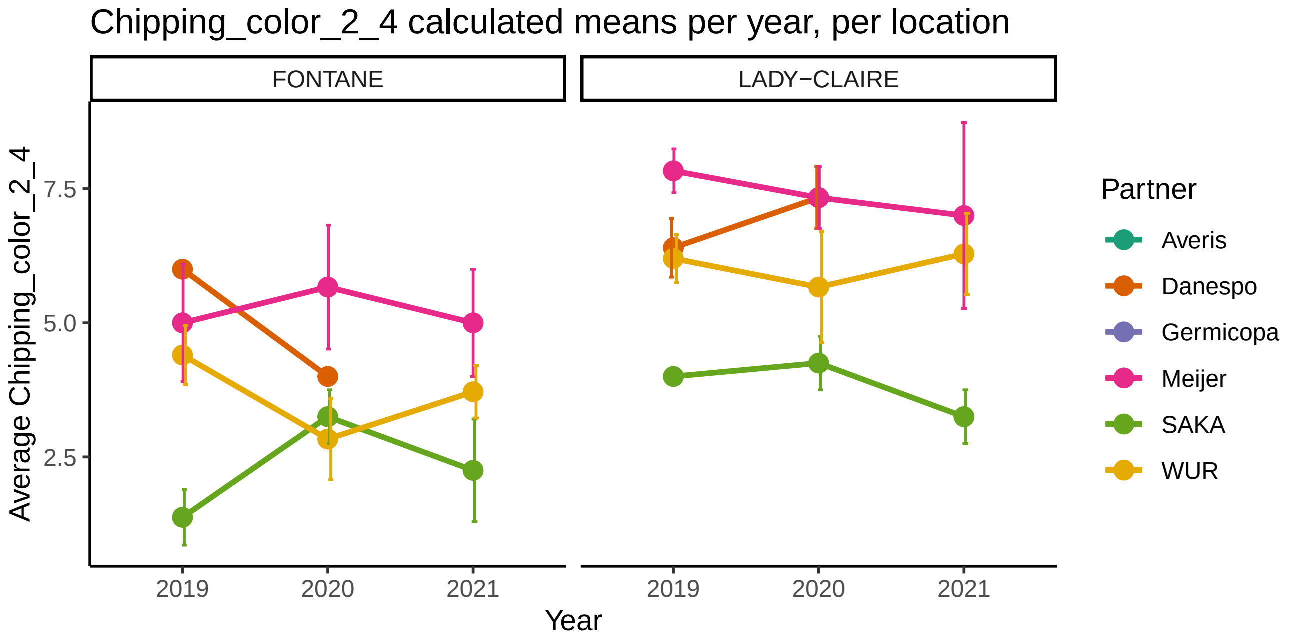
Chipping_color_2_4 calculated means per year, per location
FONTANE
LADY−CLAIRE
Average Chipping_color_2_4
7.5
5.0
2.5
2019
2020
2021
Year
Partner
Averis
Danespo
Germicopa
Meijer
SAKA
WUR

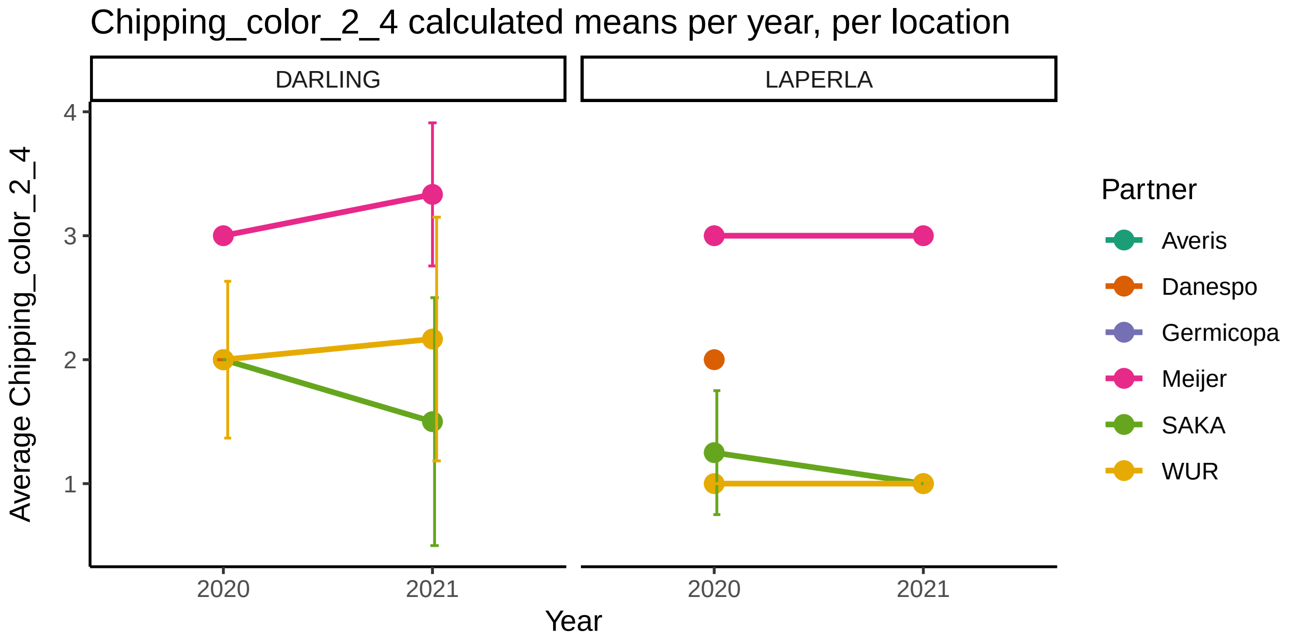
Chipping_color_2_4 calculated means per year, per location
DARLING
LAPERLA
Average Chipping_color_2_4
4
3
2
1
2020
2021
Year
Partner
Averis
Danespo
Germicopa
Meijer
SAKA
WUR

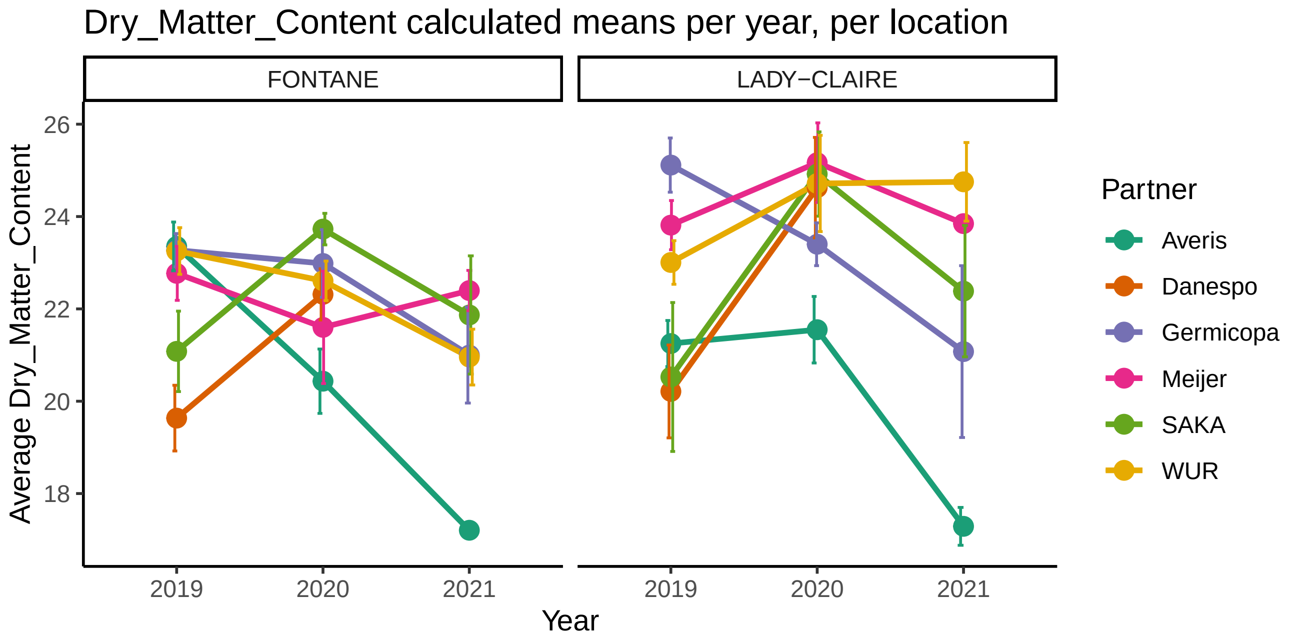
Dry_Matter_Content calculated means per year, per location
FONTANE
LADY−CLAIRE
Average Dry_Matter_Content
26
24
22
20
18
2019
2020
2021
Year
Partner
Averis
Danespo
Germicopa
Meijer
SAKA
WUR

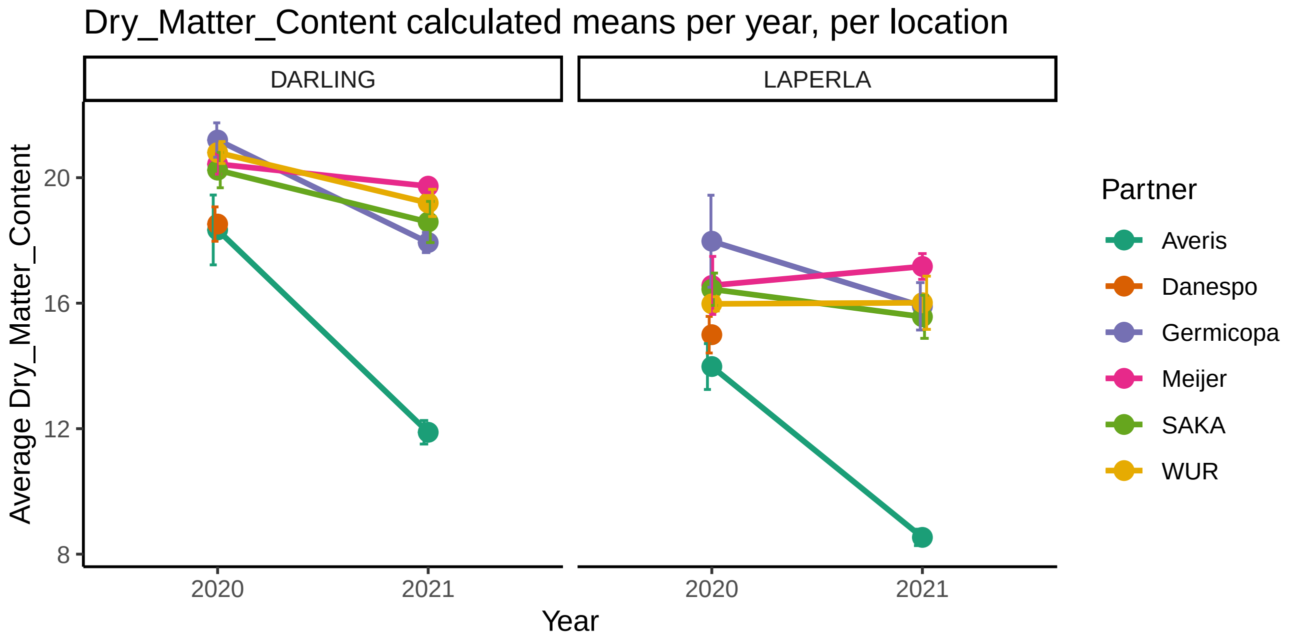
Dry_Matter_Content calculated means per year, per location
DARLING
LAPERLA
Average Dry_Matter_Content
20
16
12
8
2020
2021
Year
Partner
Averis
Danespo
Germicopa
Meijer
SAKA
WUR

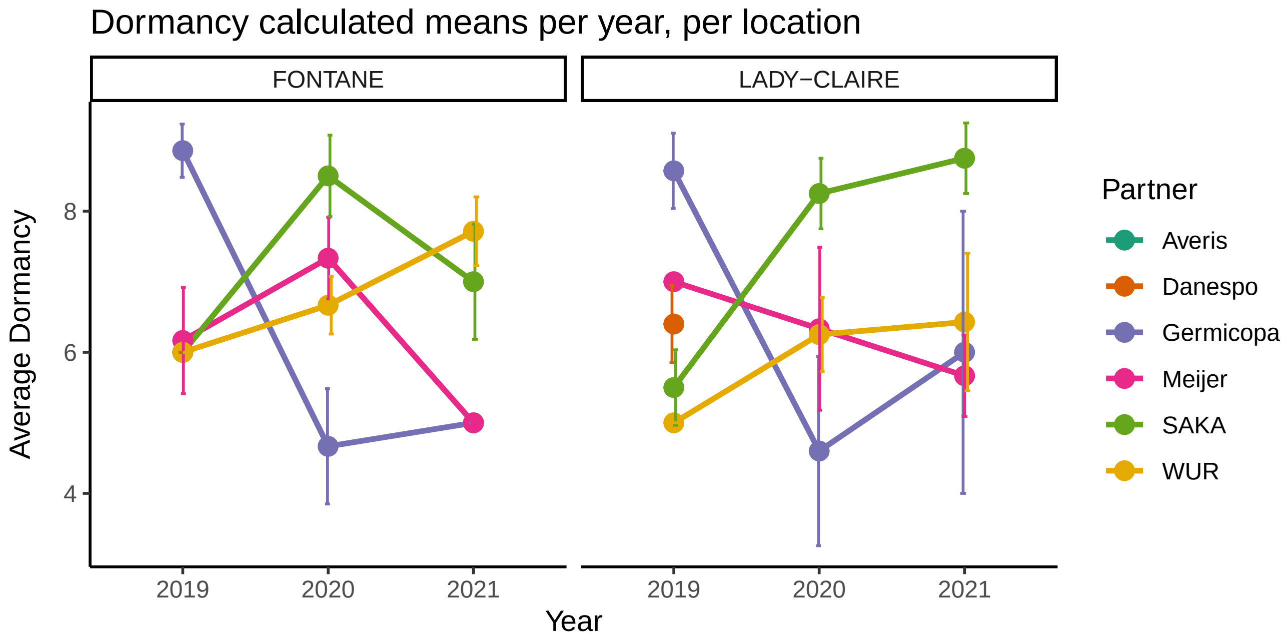

Dormancy calculated means per year, per location
FONTANE
LADY−CLAIRE
Average Dormancy
8
6
4
2019
2020
2021
Year
Partner
Averis
Danespo
Germicopa
Meijer
SAKA
WUR

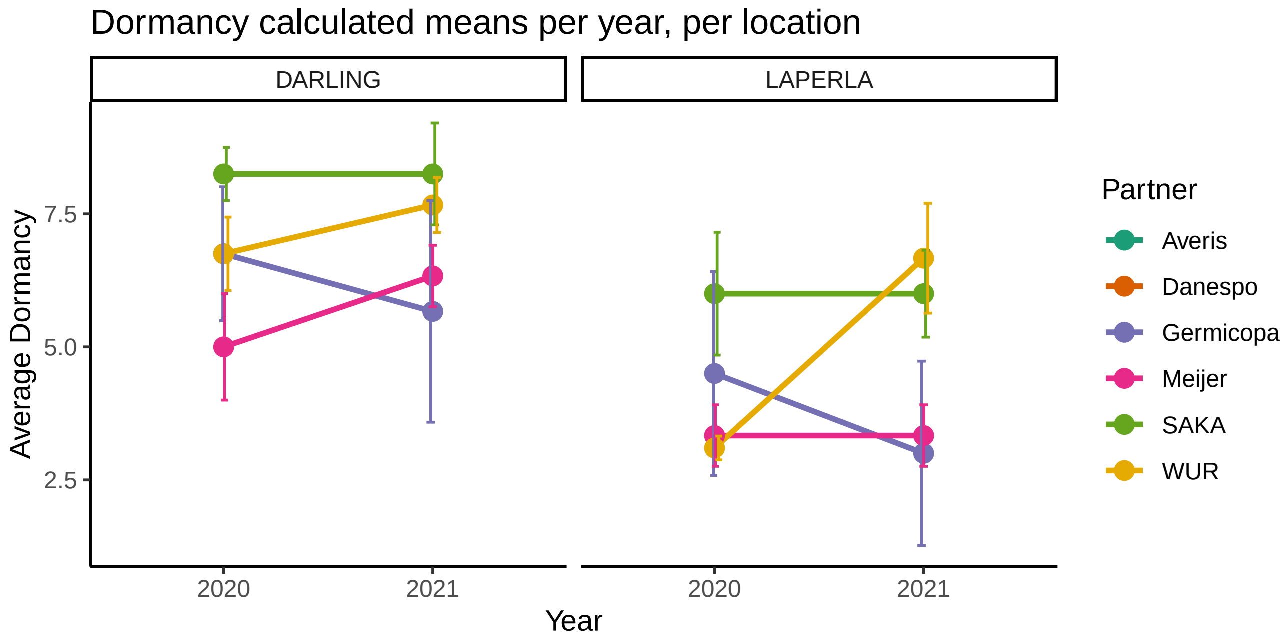

Dormancy calculated means per year, per location
DARLING
LAPERLA
Average Dormancy
7.5
5.0
2.5
2020
2021
Year
Partner
Averis
Danespo
Germicopa
Meijer
SAKA
WUR

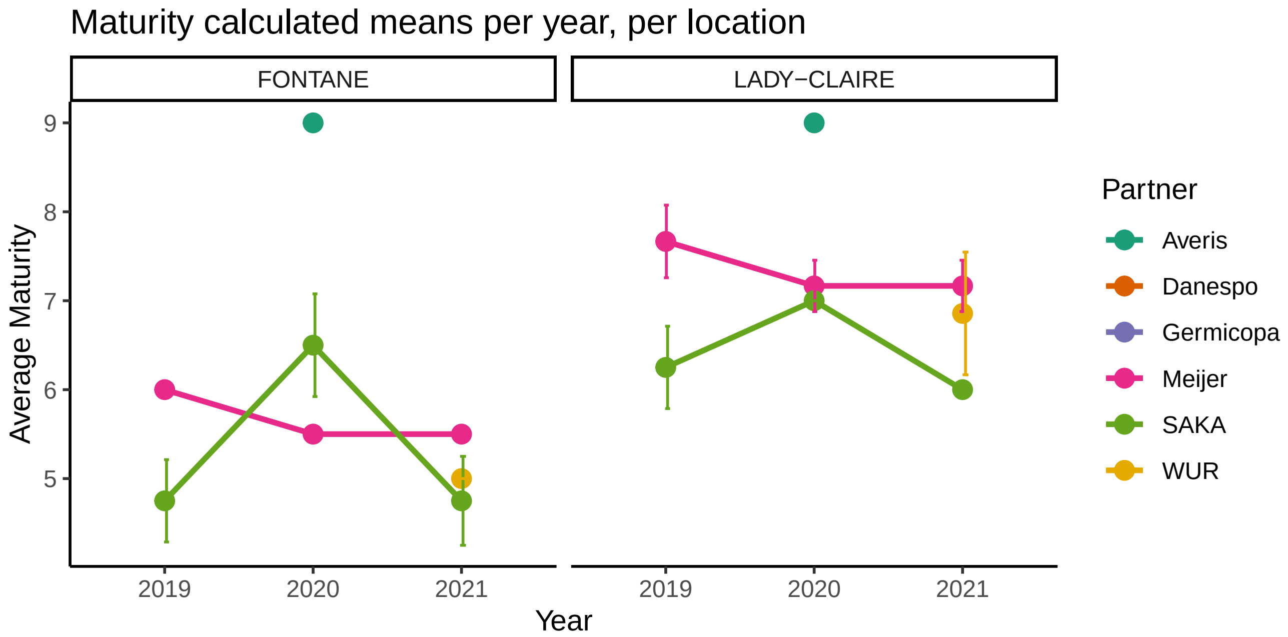
Maturity calculated means per year, per location
FONTANE
LADY−CLAIRE
Average Maturity
Year
Partner
Averis
Danespo
Germicopa
Meijer
SAKA
WUR

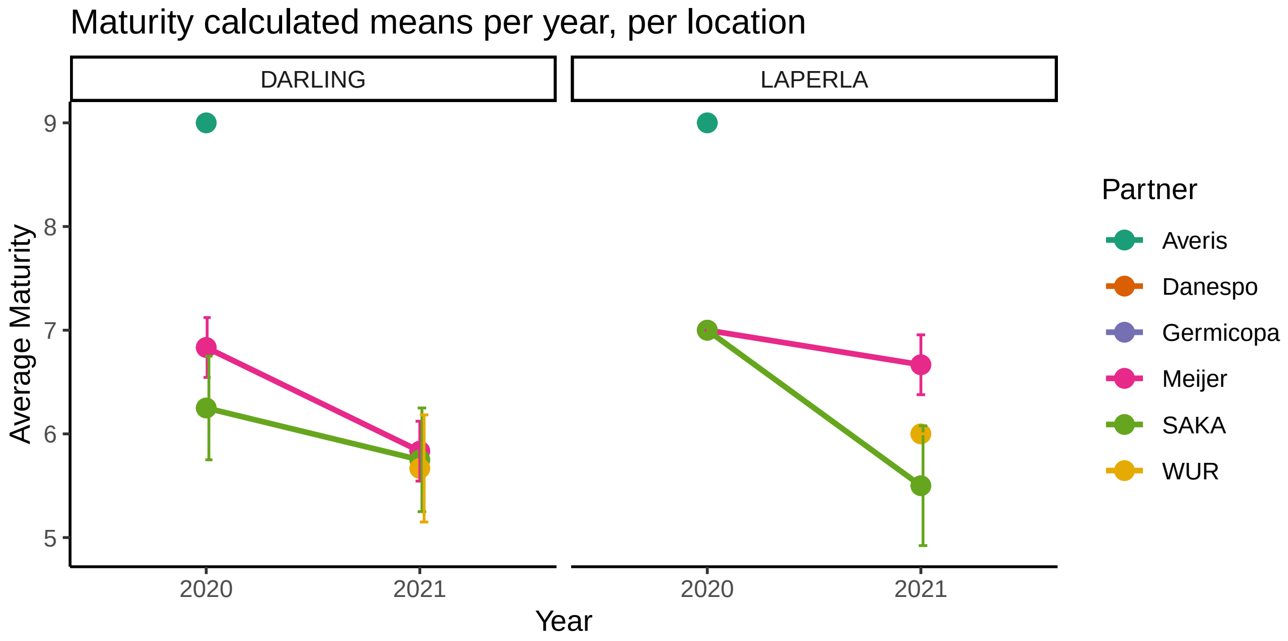
Maturity calculated means per year, per location
DARLING
LAPERLA
Average Maturity
Year
Partner
Averis
Danespo
Germicopa
Meijer
SAKA
WUR
