# Supplementary material for: QTL discovery for agronomic and quality traits in diploid potato clones using PotatoMASH amplicon sequencing
Source: G3 (Bethesda). 2024 Jul 19;14(10):jkae164. doi: 10.1093/g3journal/jkae164 (PMC11457057; doi:10.1093/g3journal/jkae164)
Supplement: jkae164_Supplementary_Data [file jkae164_supplementary_data.zip › Supplemental_Material_Legends_G3-2024-405051.docx]

## Supplementary files

Supplement file 1 - Experimental plot information

Supplement file 2 - Correction table for the number of tubers

Supplement file 3 – Normality plots for the full panel

Supplement file 4 – SNP dosage data

Supplement file 5 – Haplotag dosage data

Supplement file 6 – Variety controls performance

Supplement file 7 - Marker-based heritability

Supplement file 8 – Estimated means all traits for the full panel

Supplement file 9 – Significant markers, data with scores, effects and R^2^

Supplement file 10 - Manhattan and qq plots for all traits available at <https://doi.org/10.6084/m9.figshare.26163616>
