# Supplementary figures and images for: QTL discovery for agronomic and quality traits in diploid potato clones using PotatoMASH amplicon sequencing
Source: G3 (Bethesda). 2024 Jul 19;14(10):jkae164. doi: 10.1093/g3journal/jkae164 (PMC11457057; doi:10.1093/g3journal/jkae164)

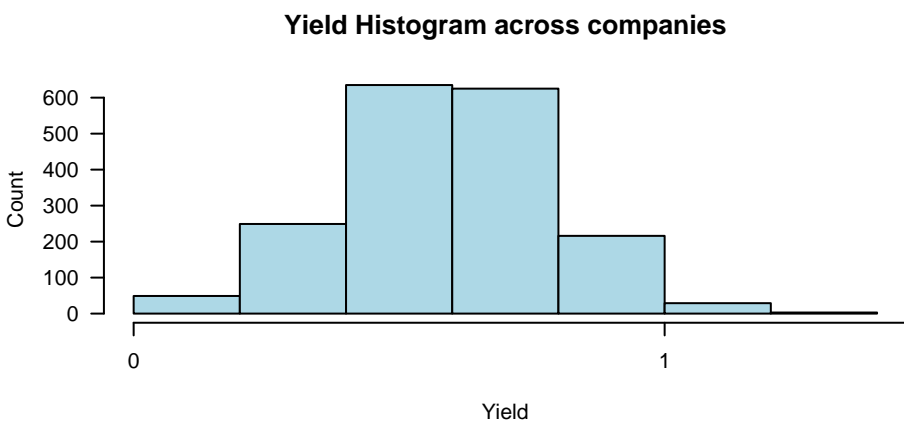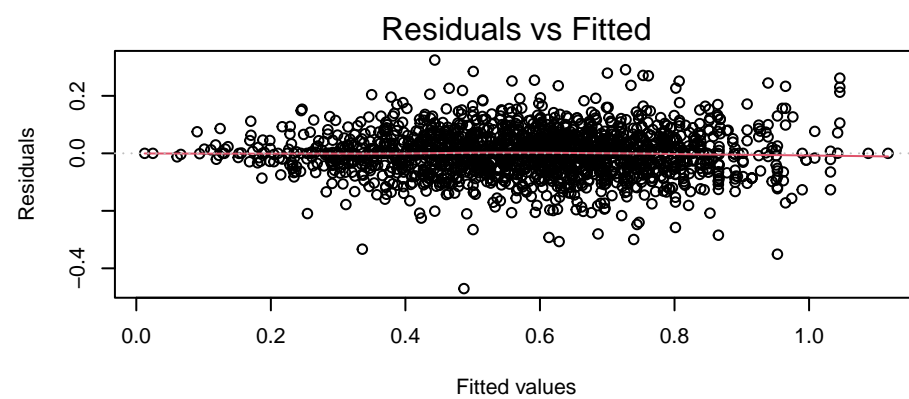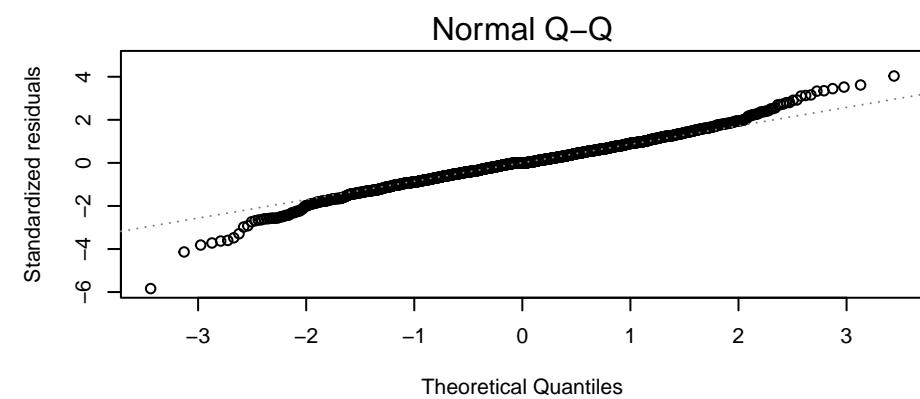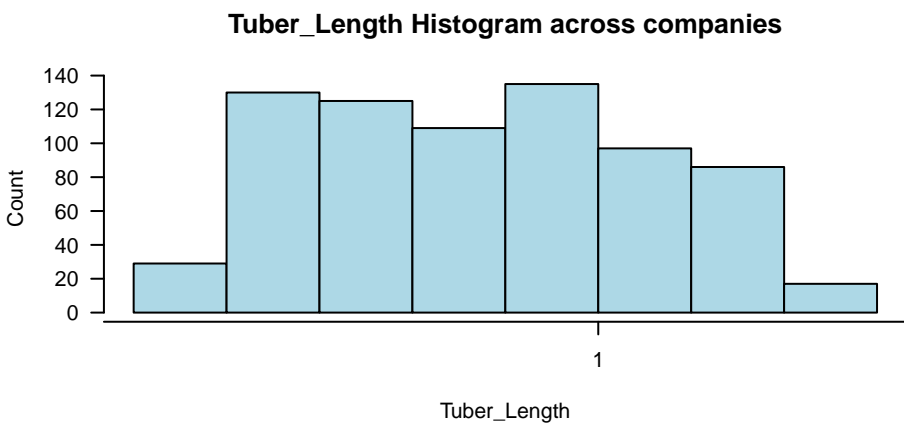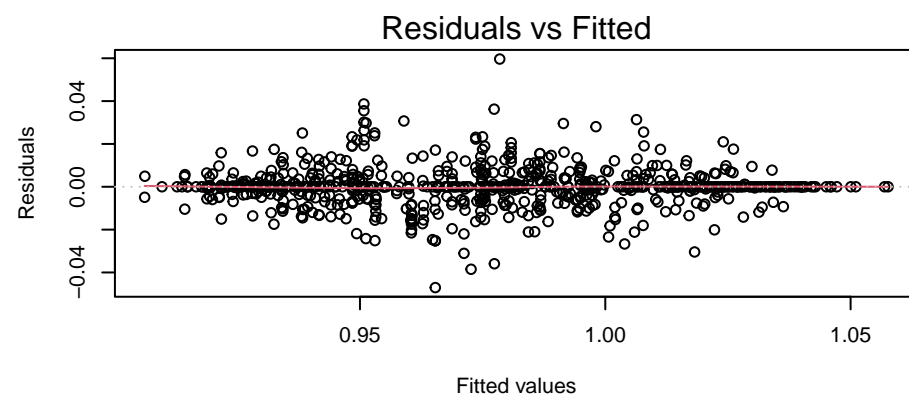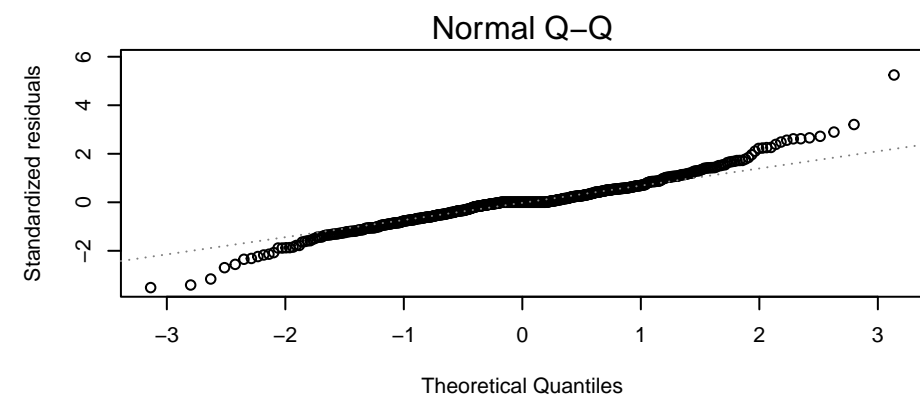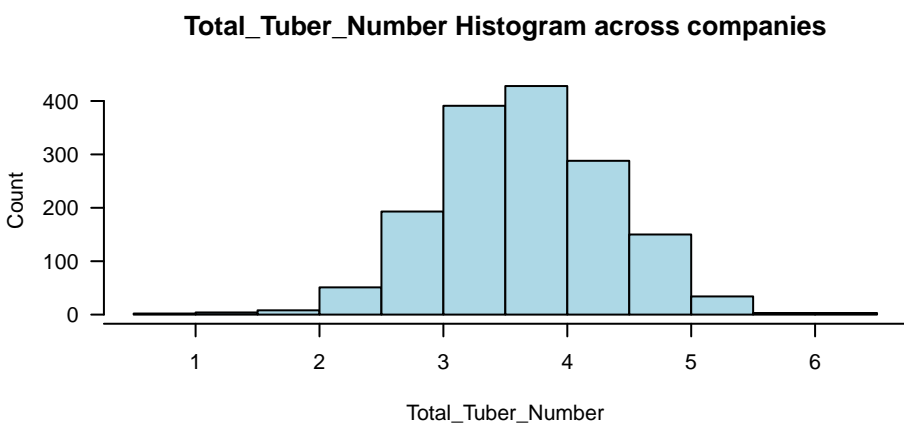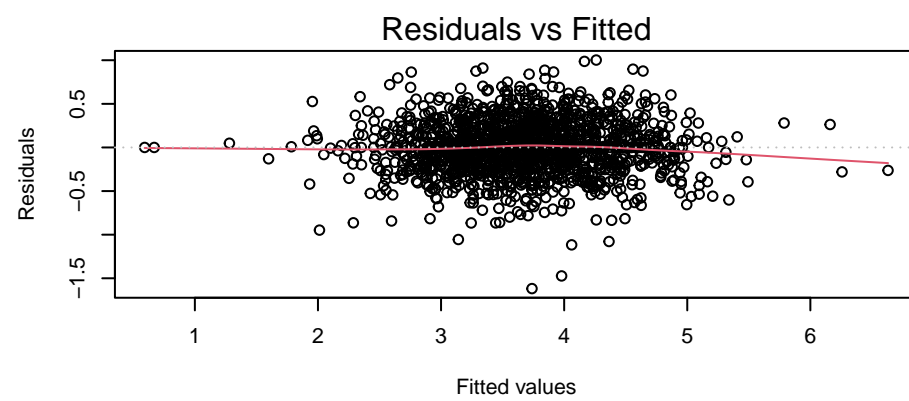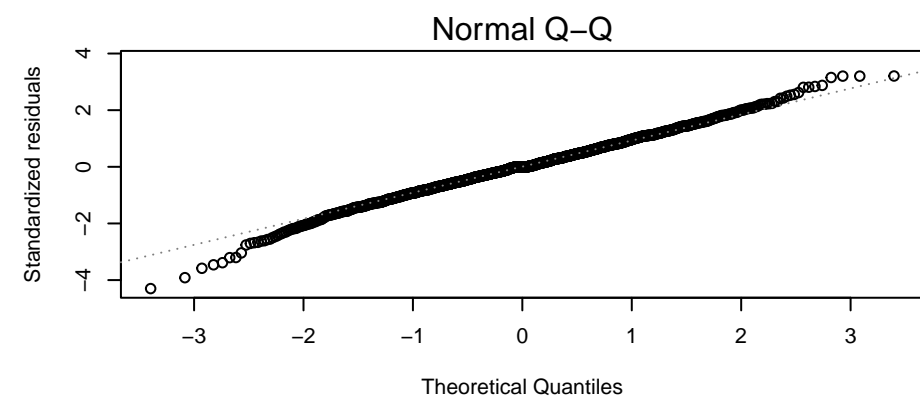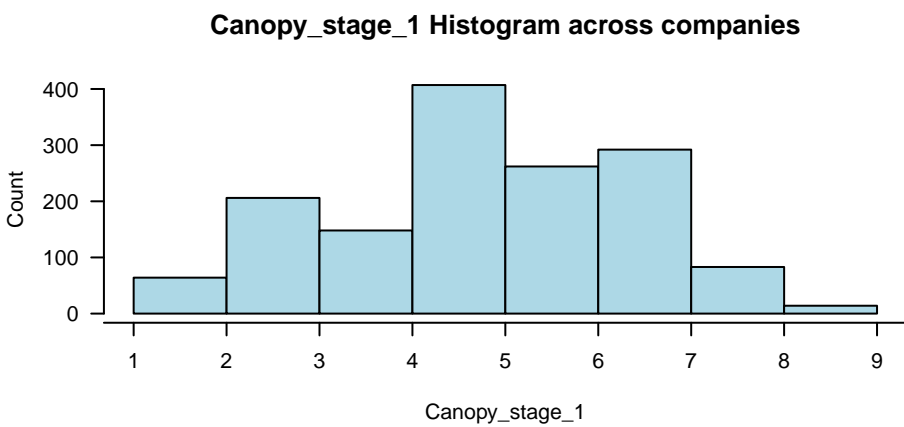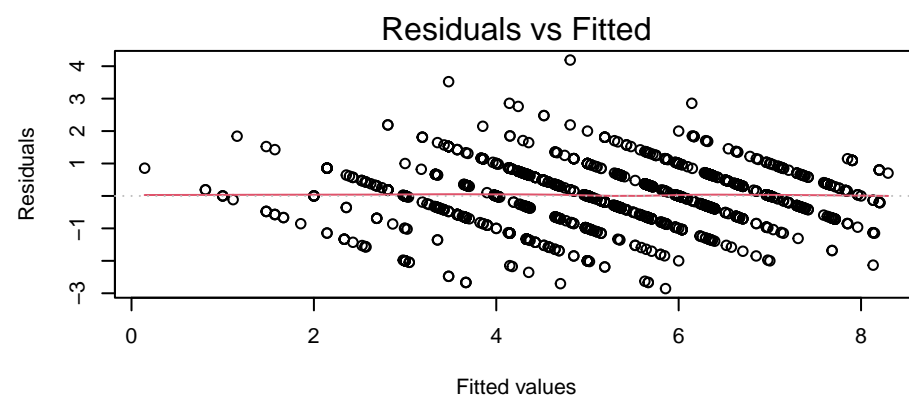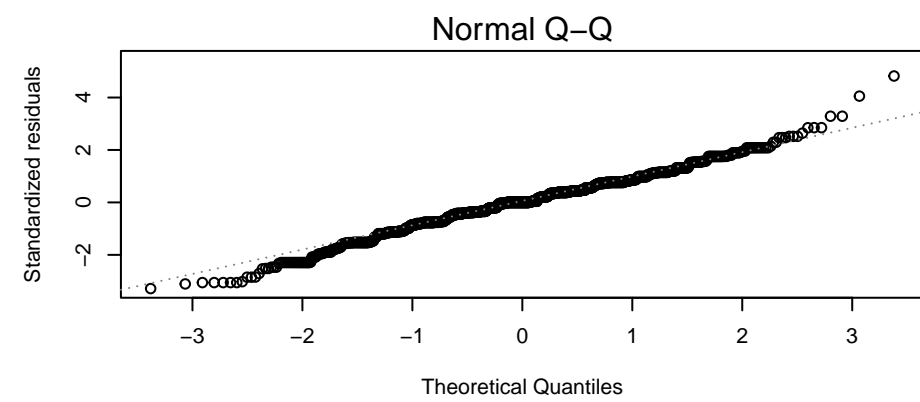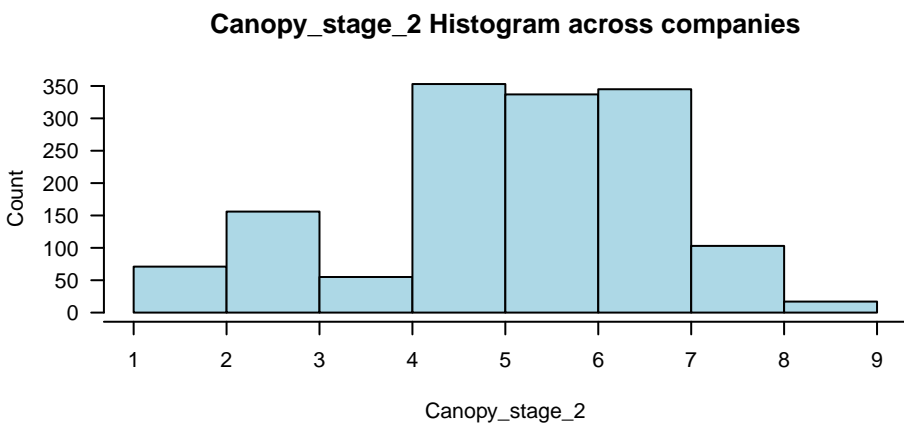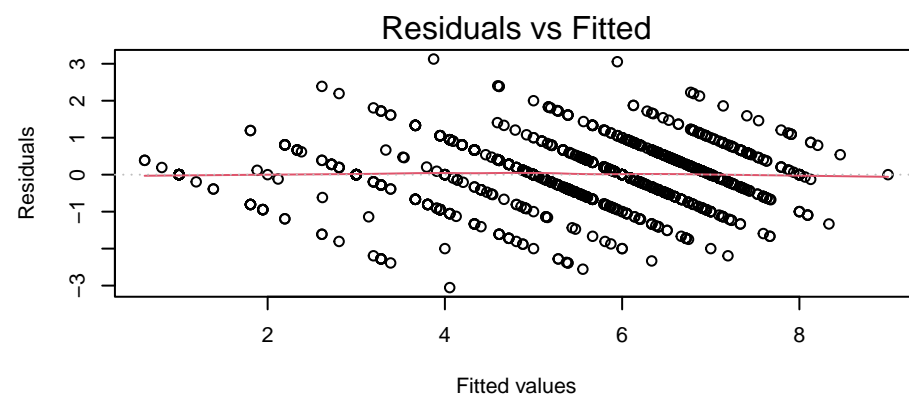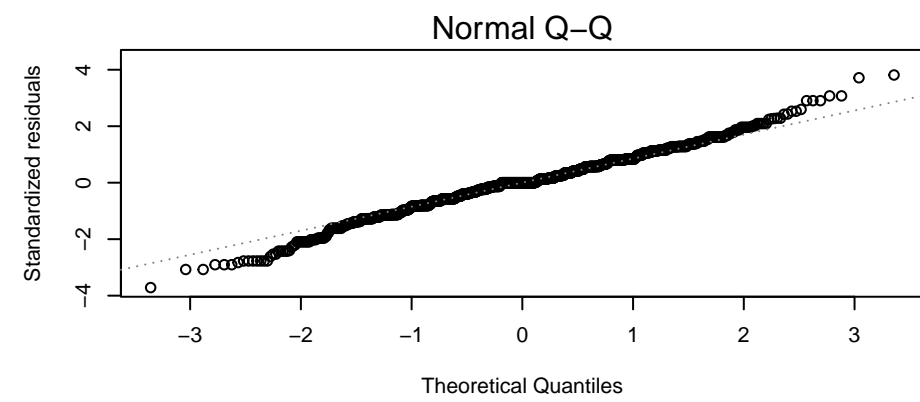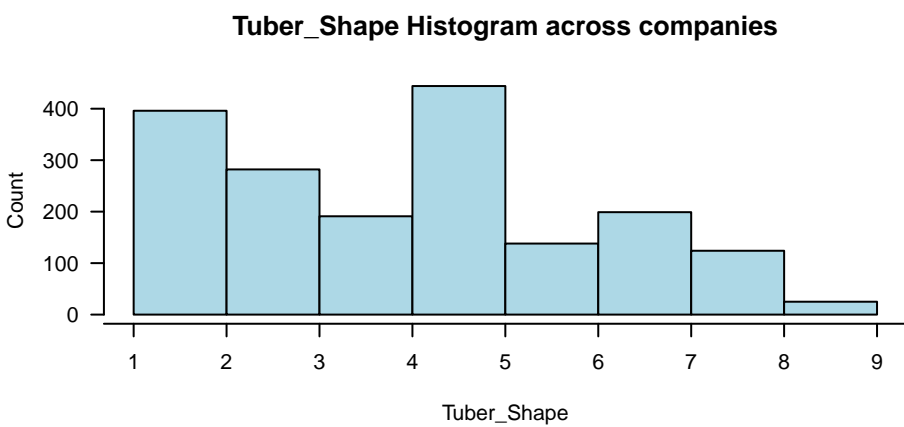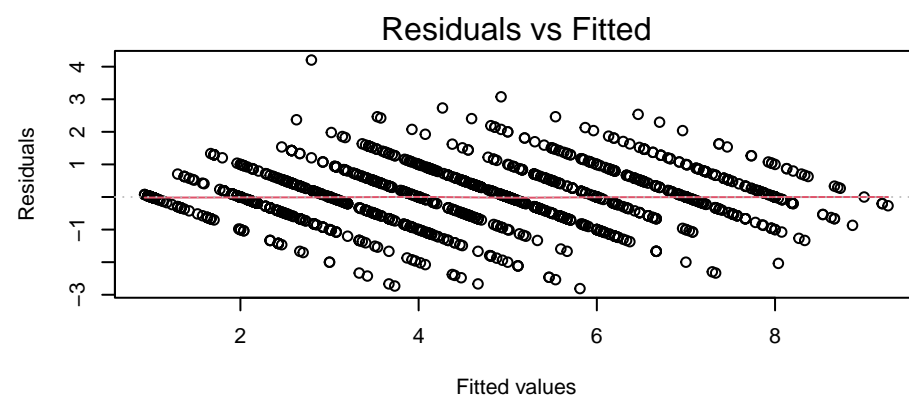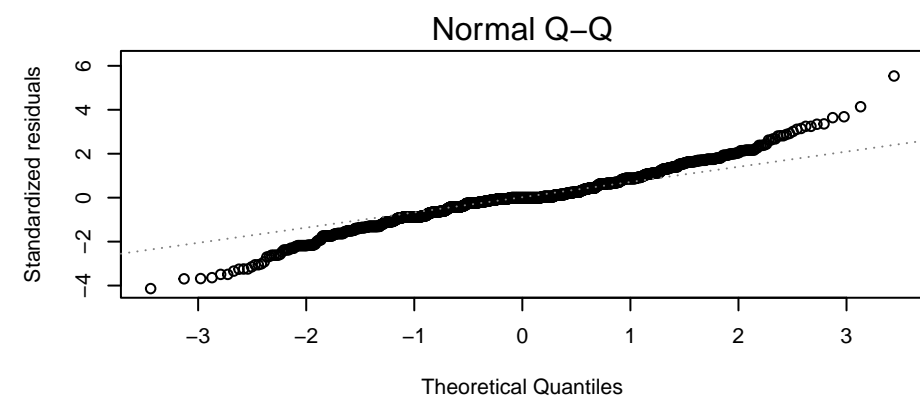

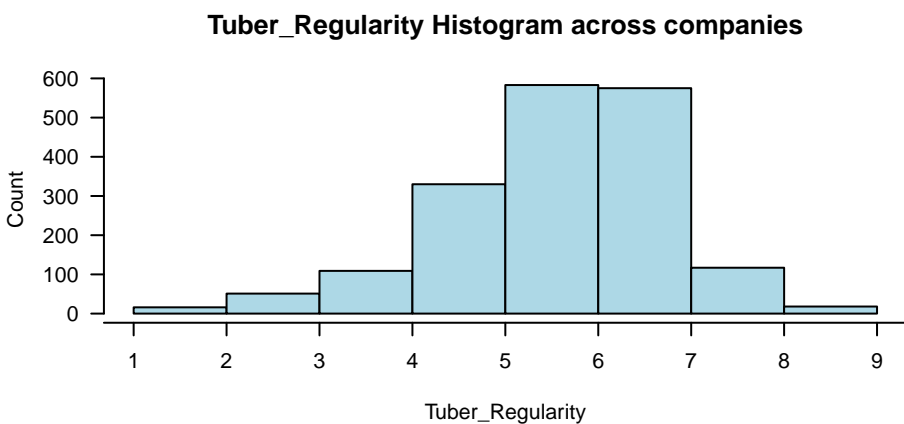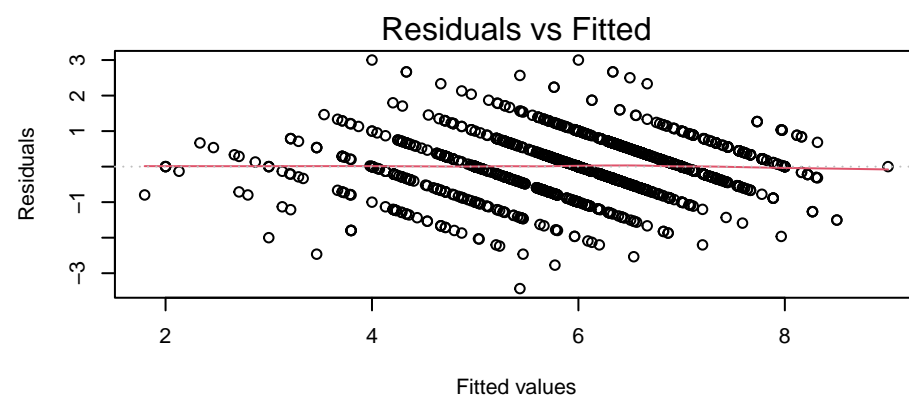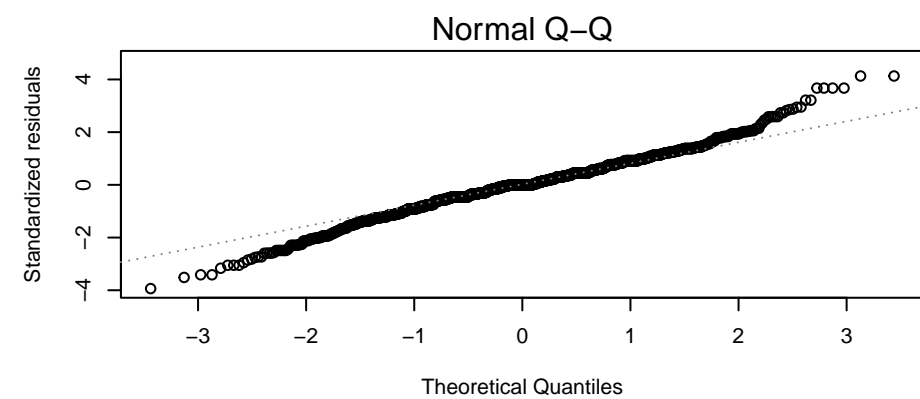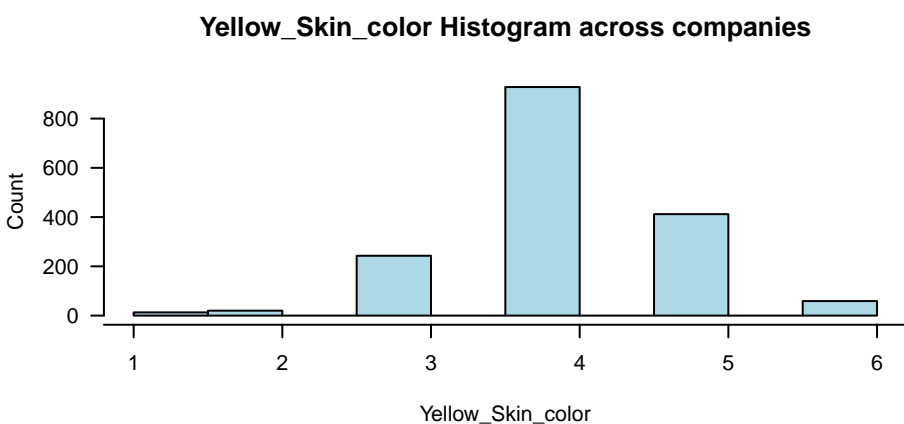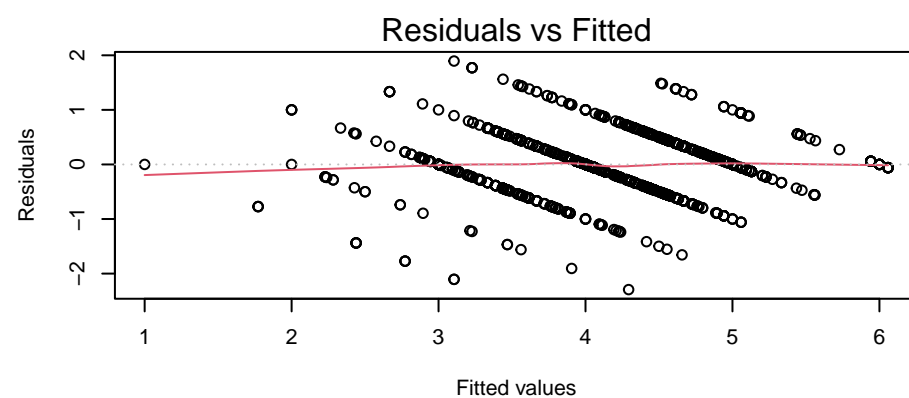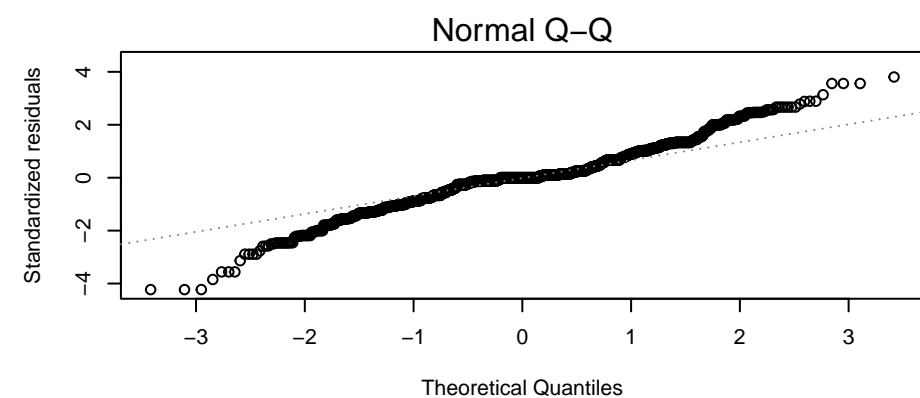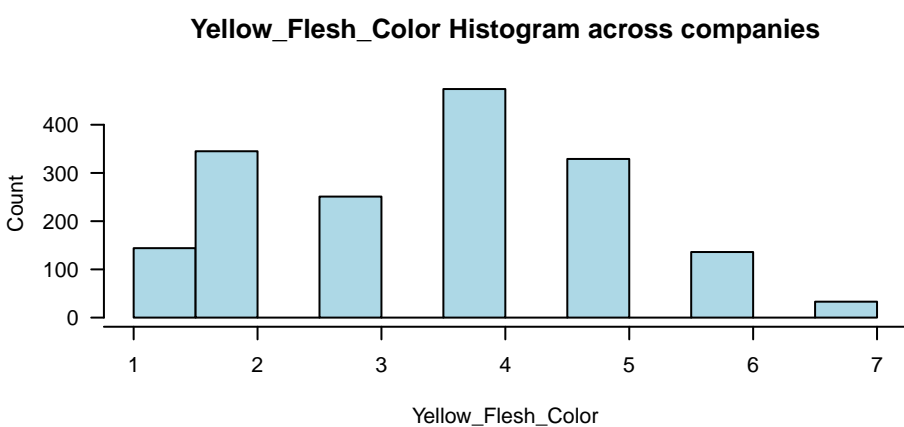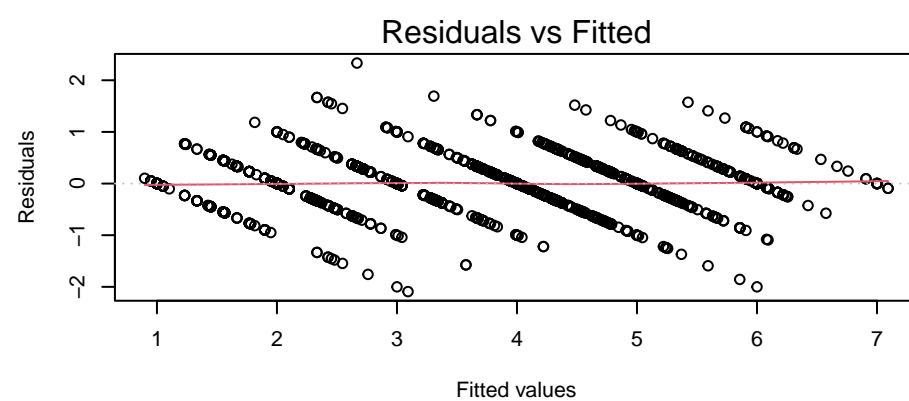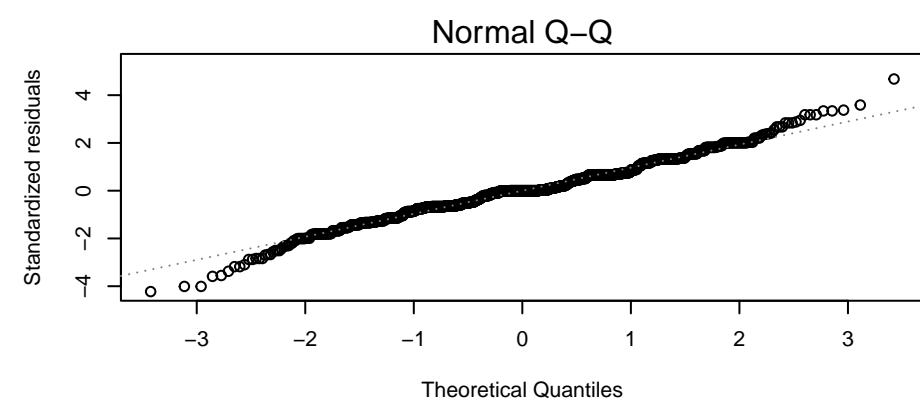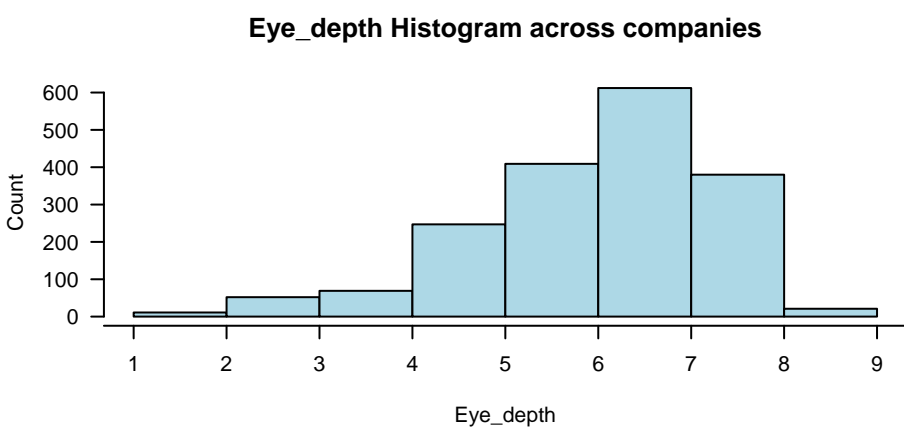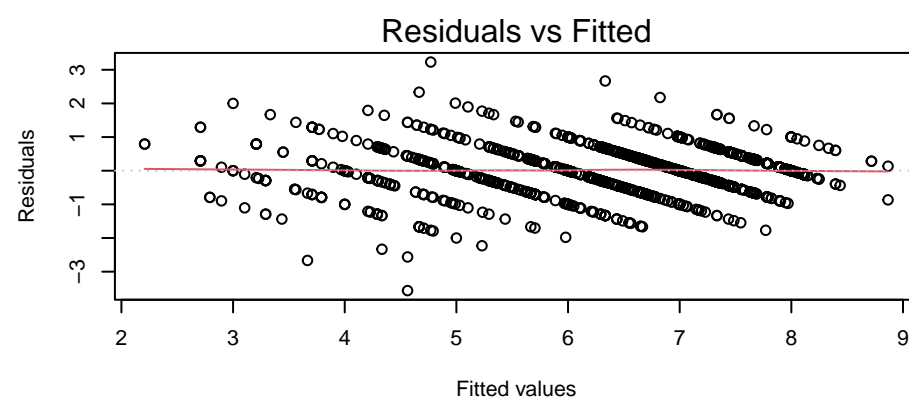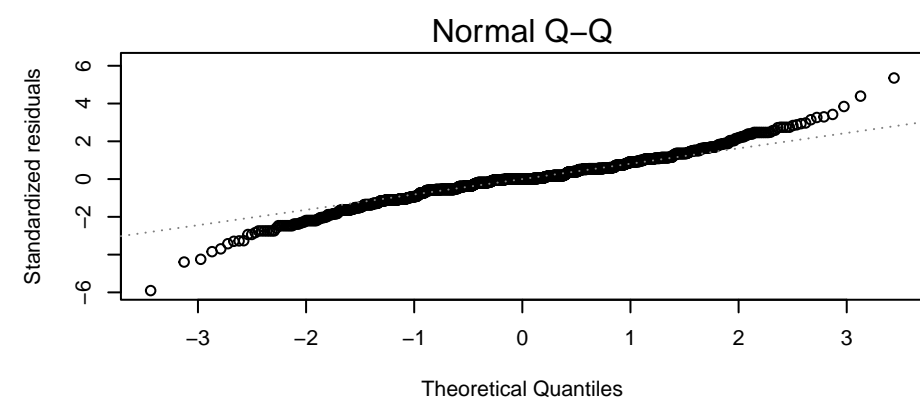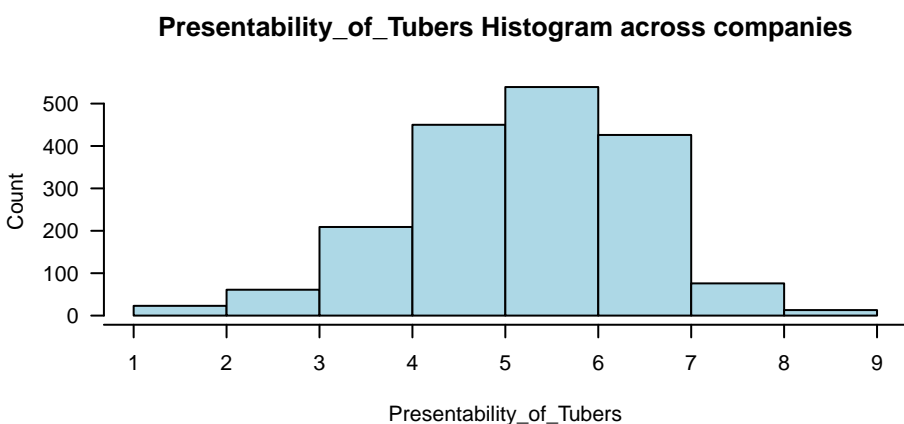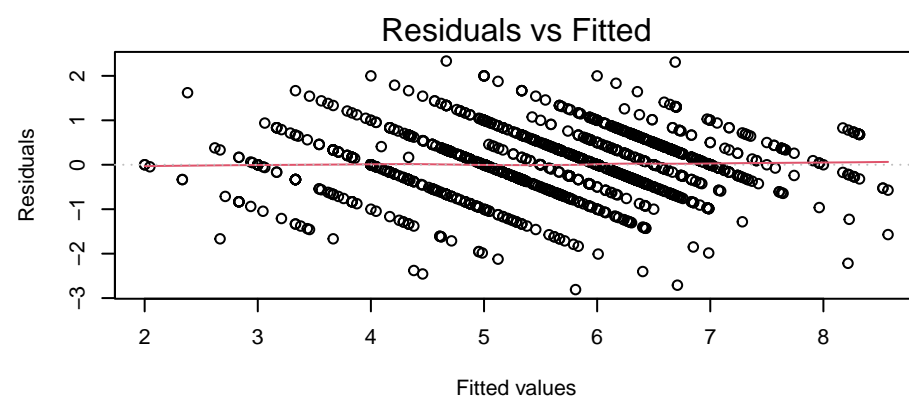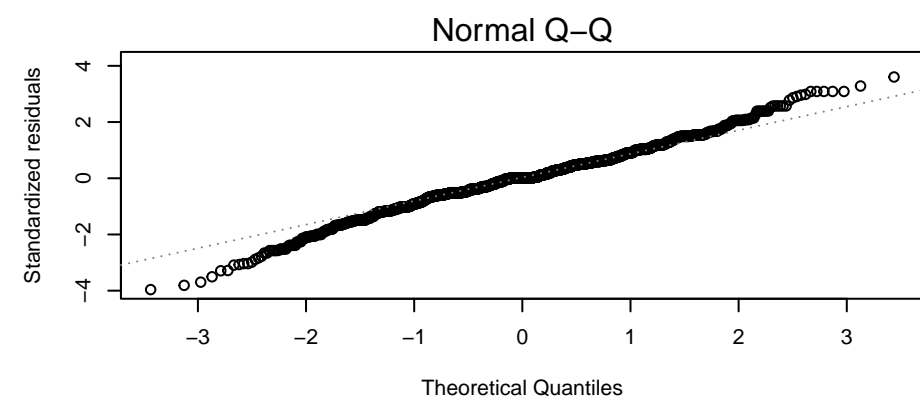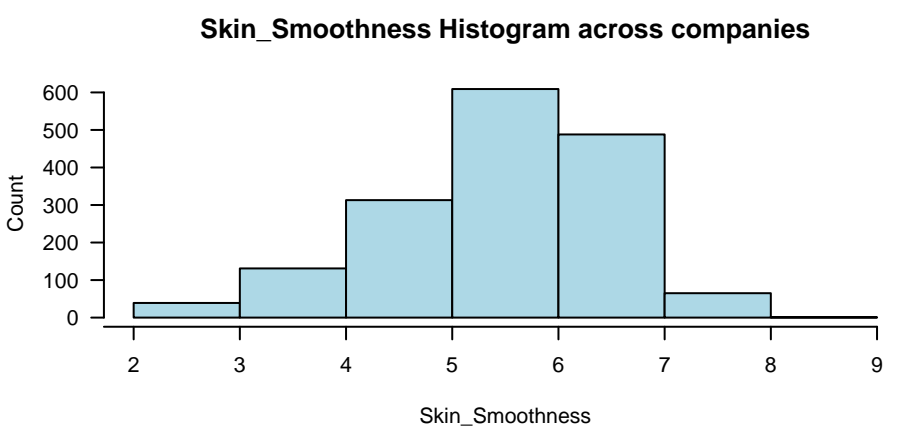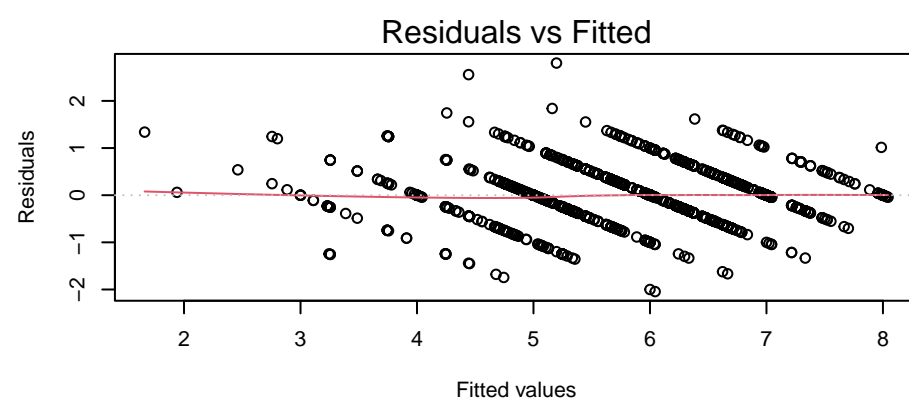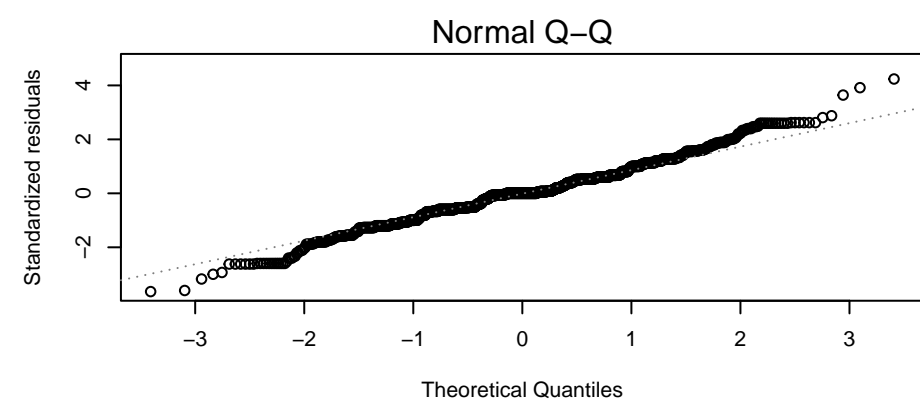

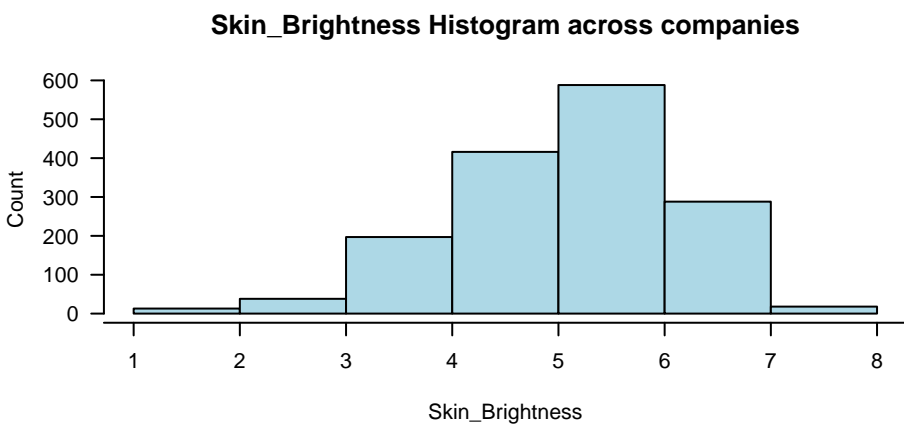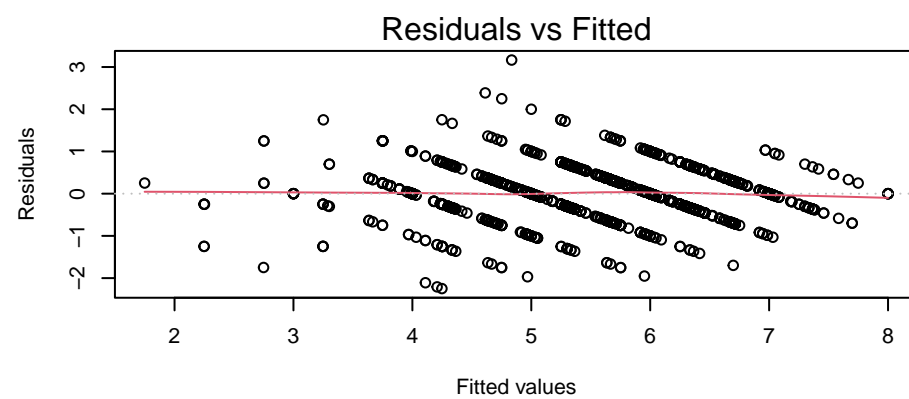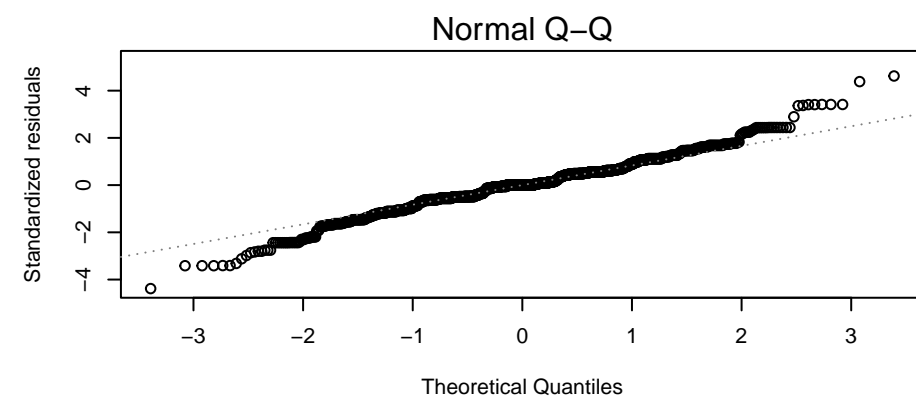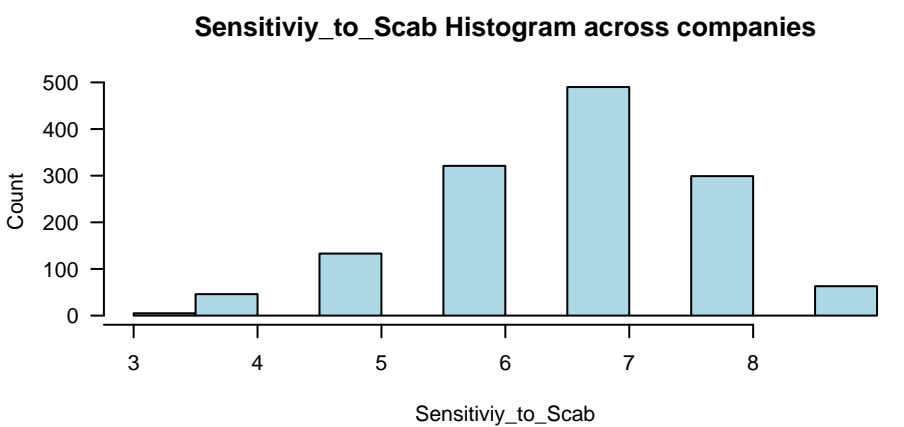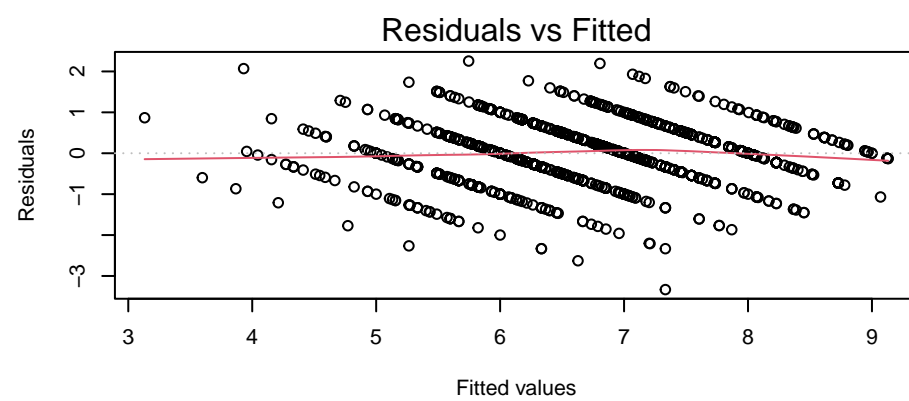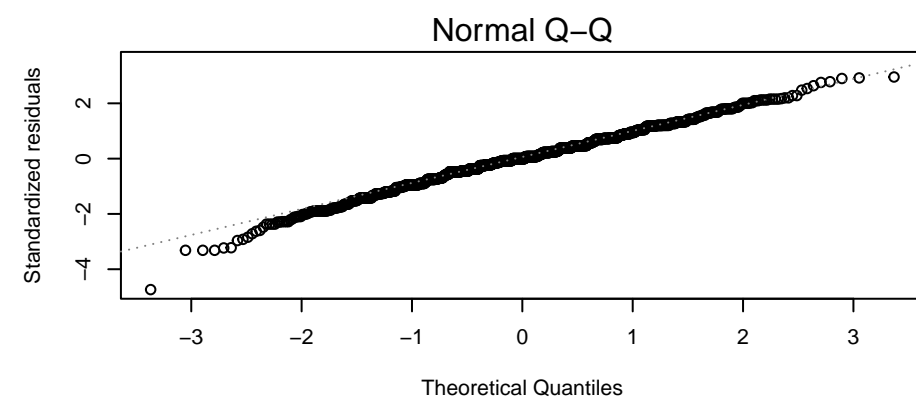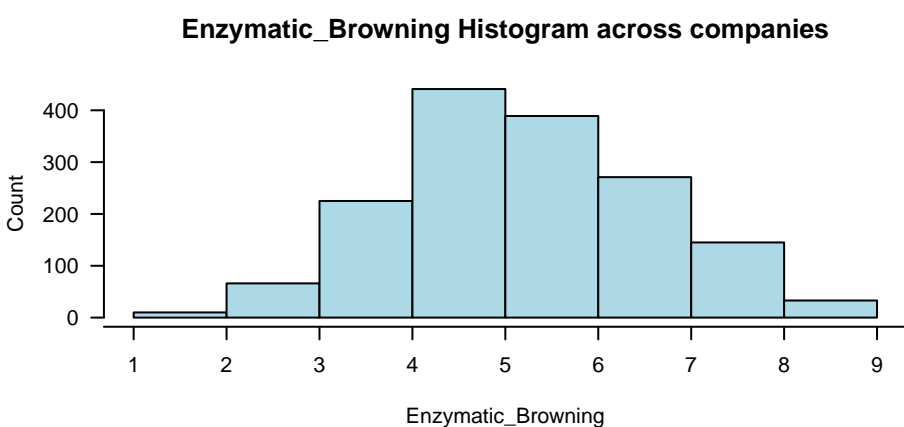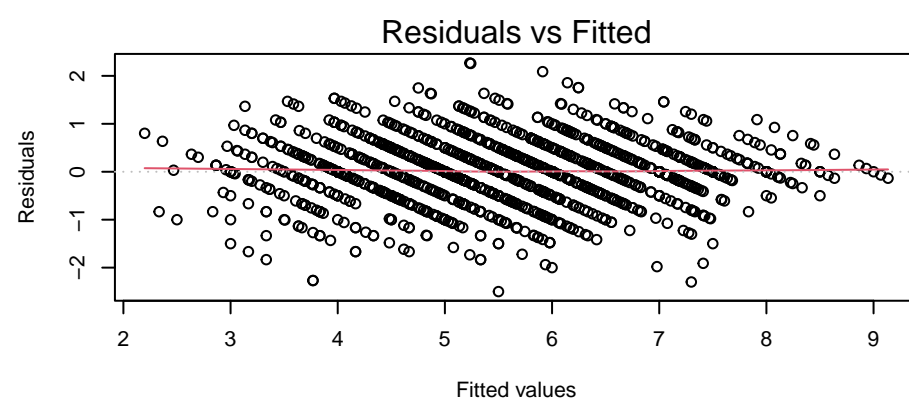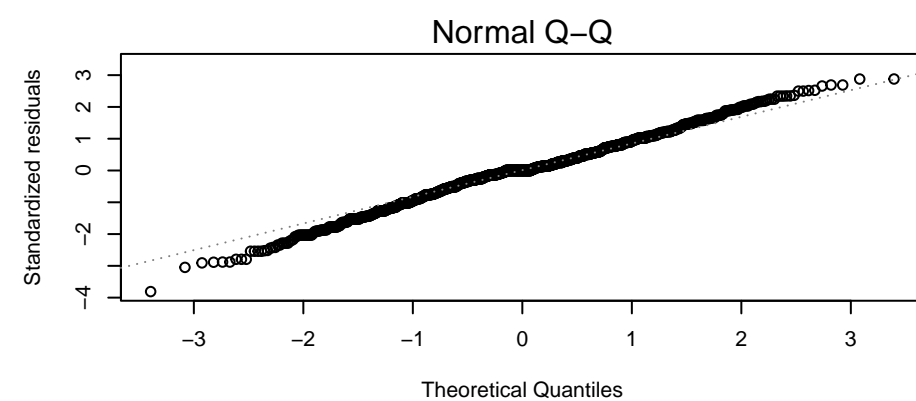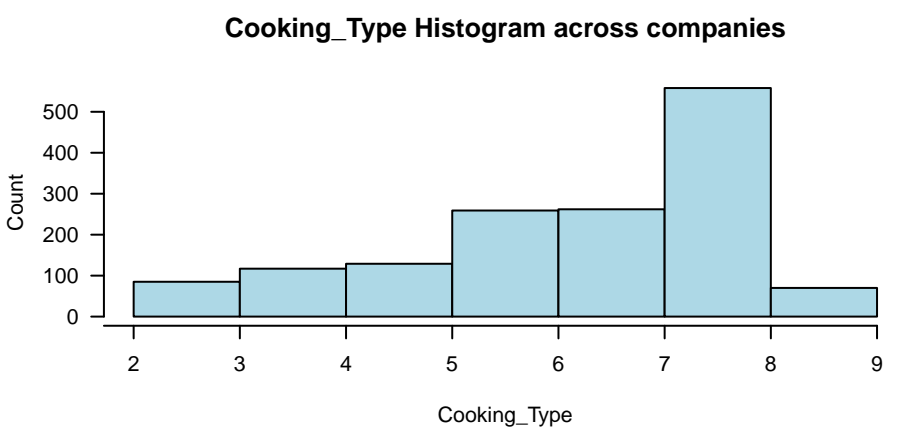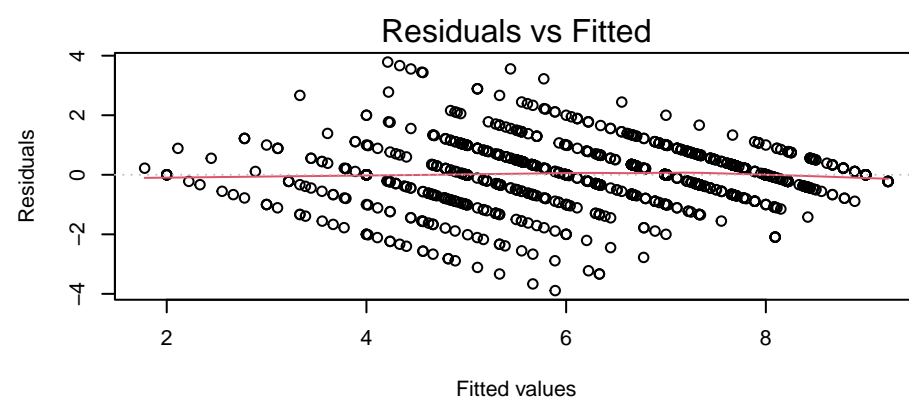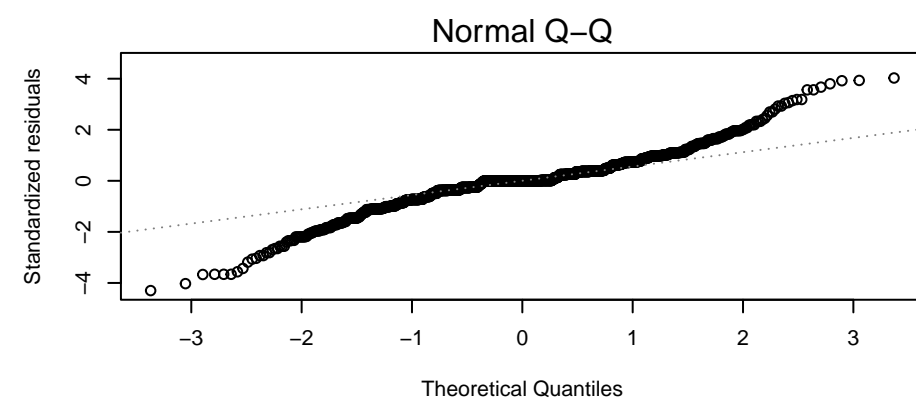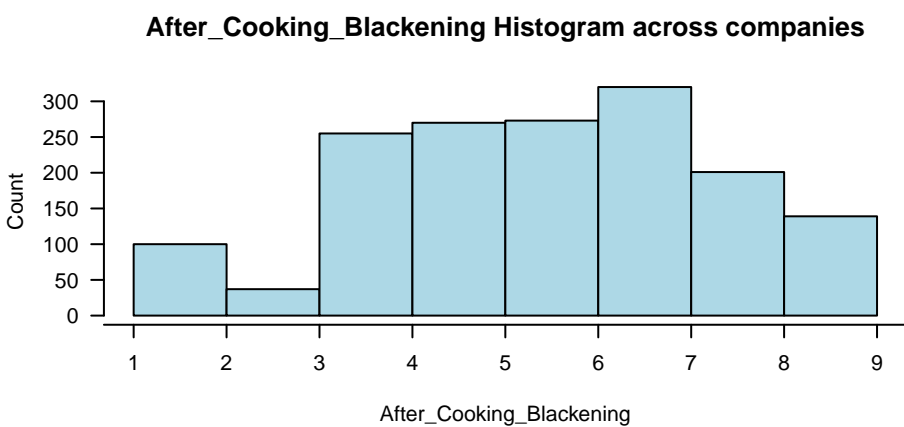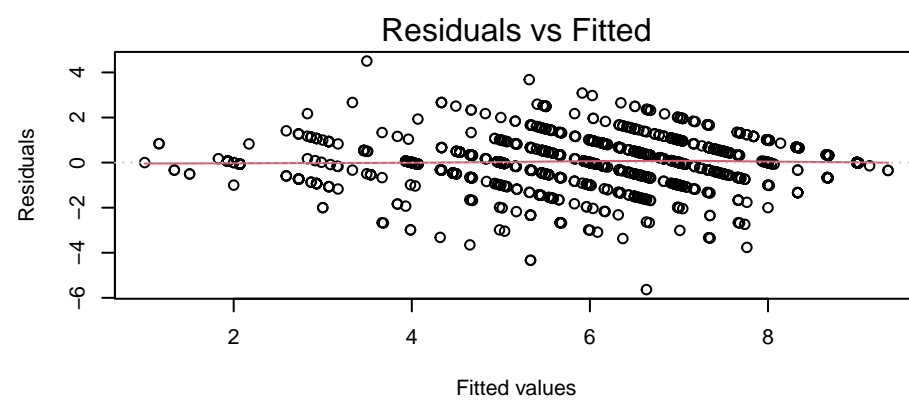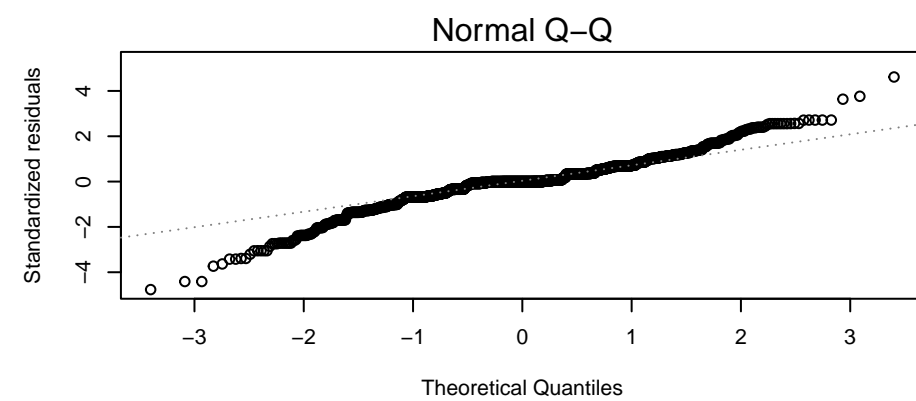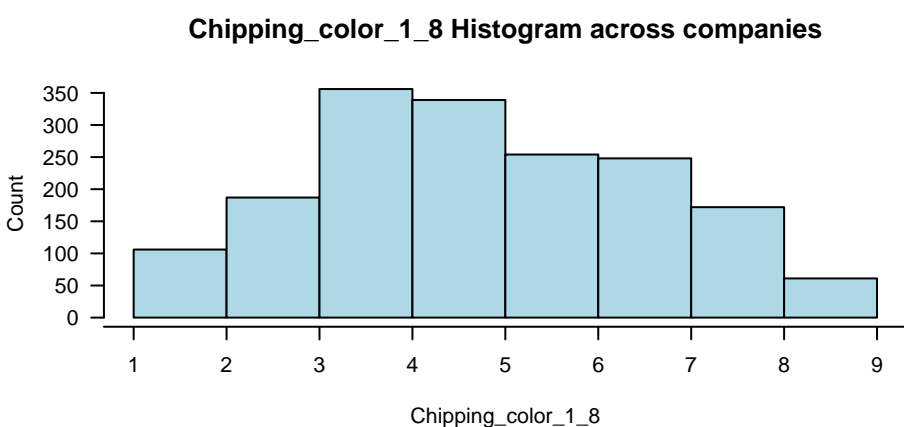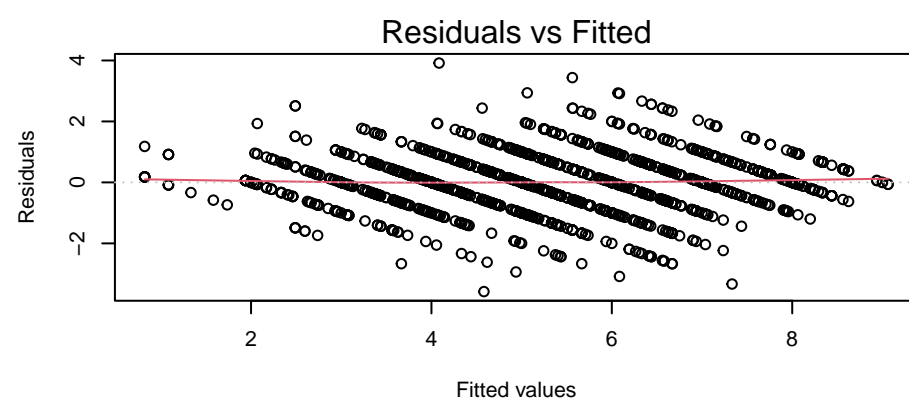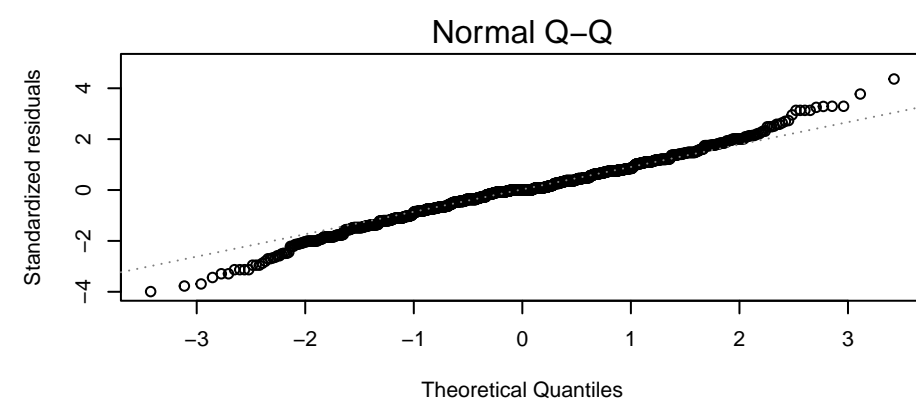

Histograms across all companies

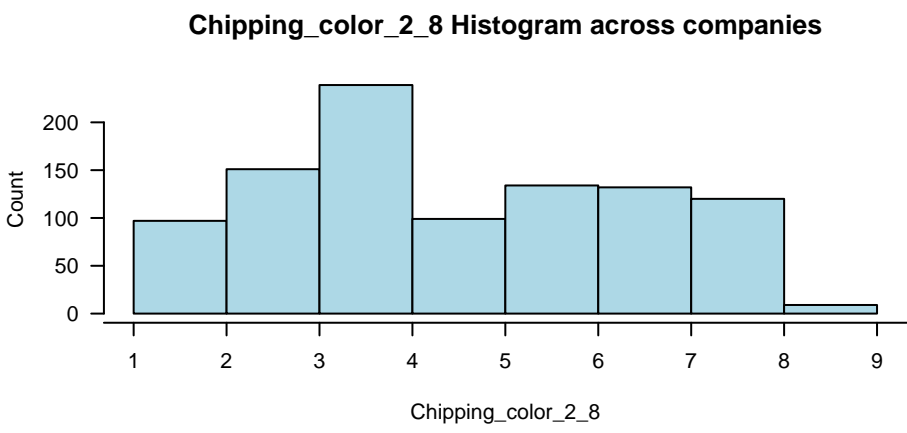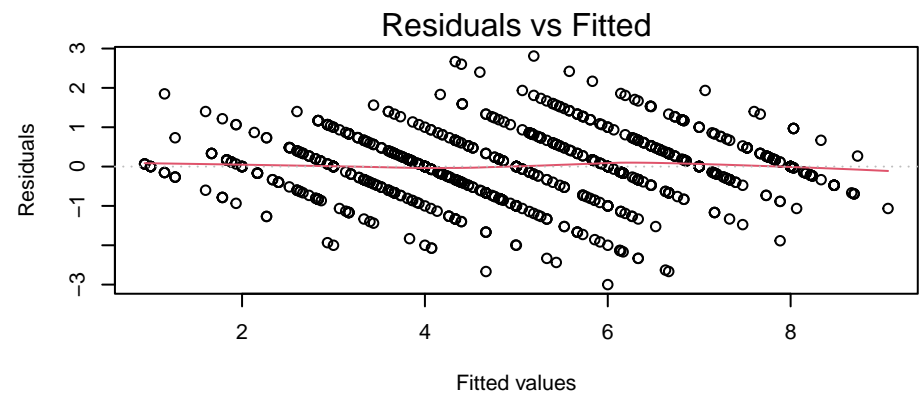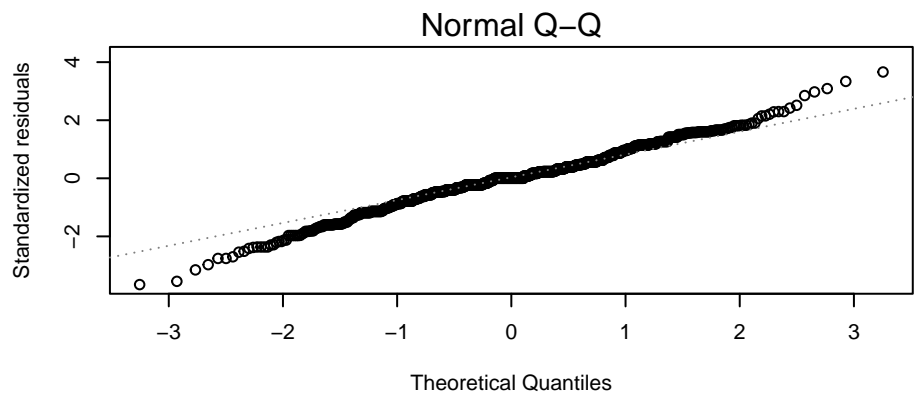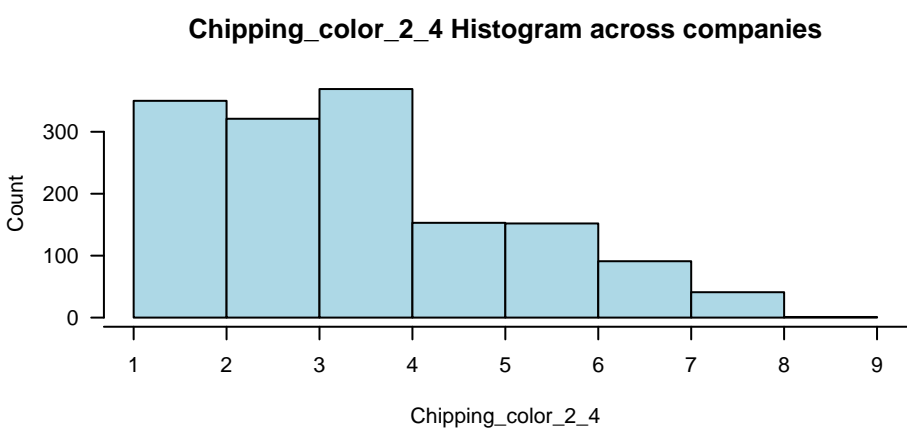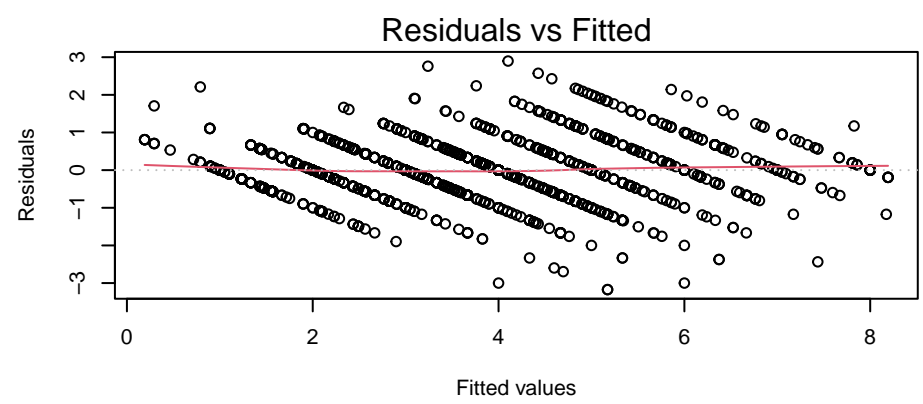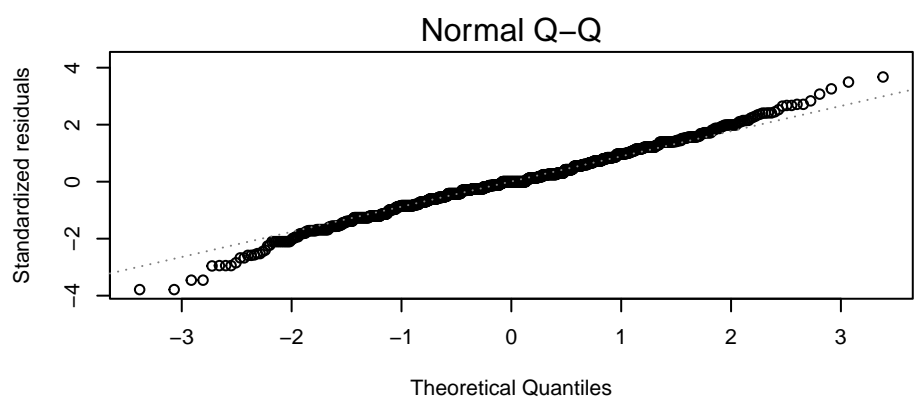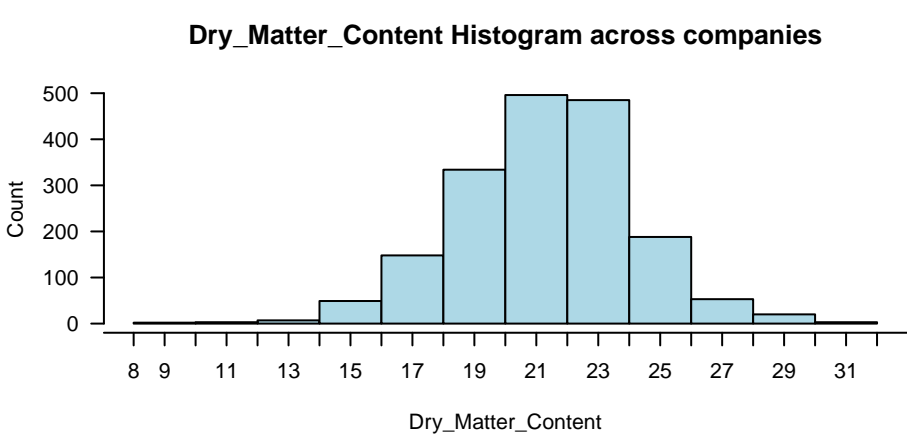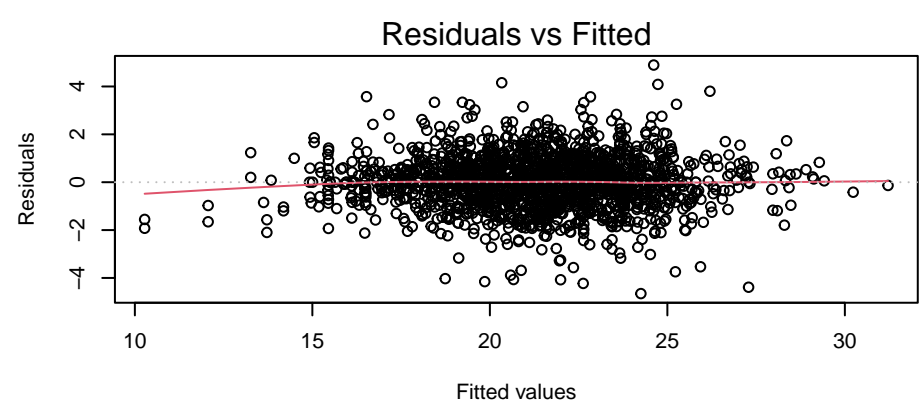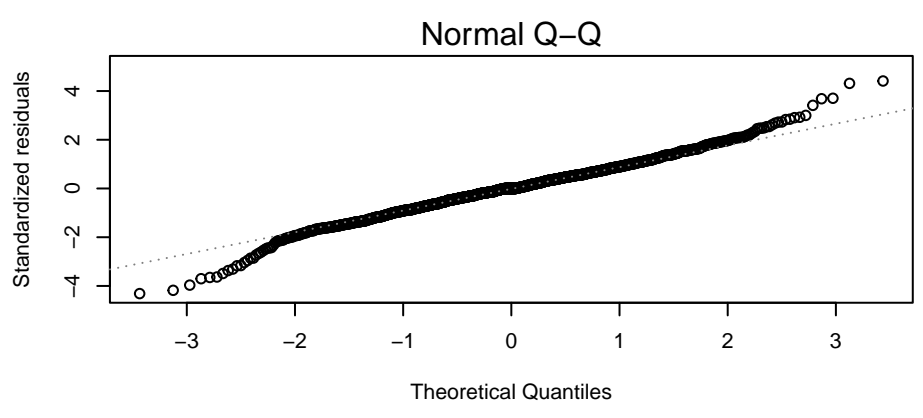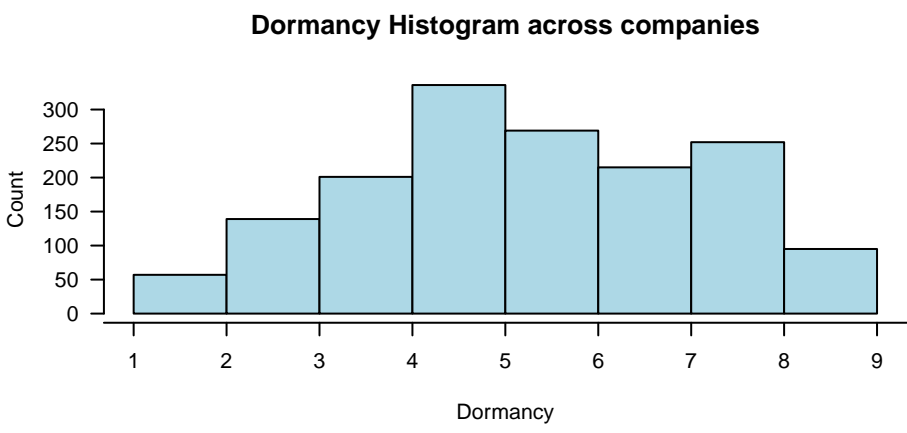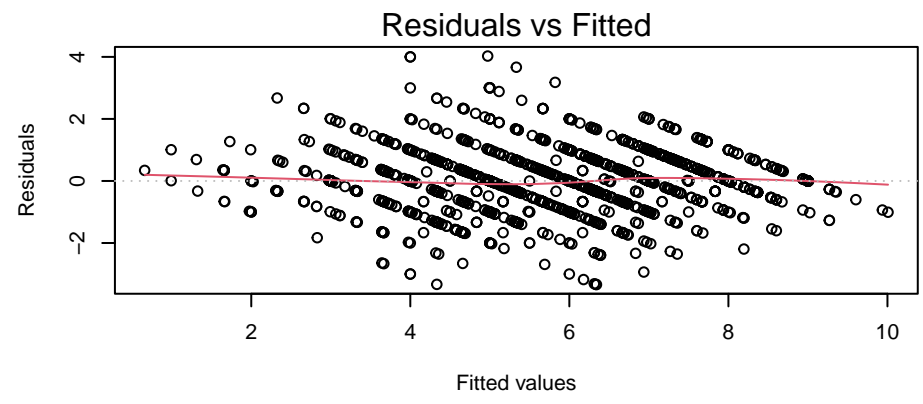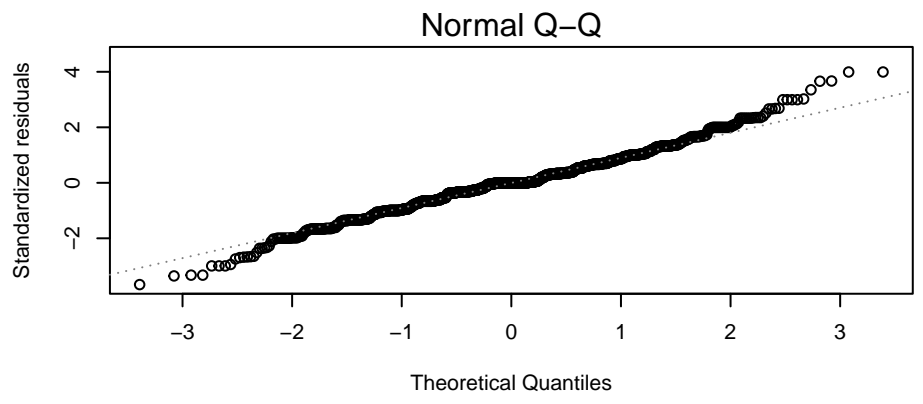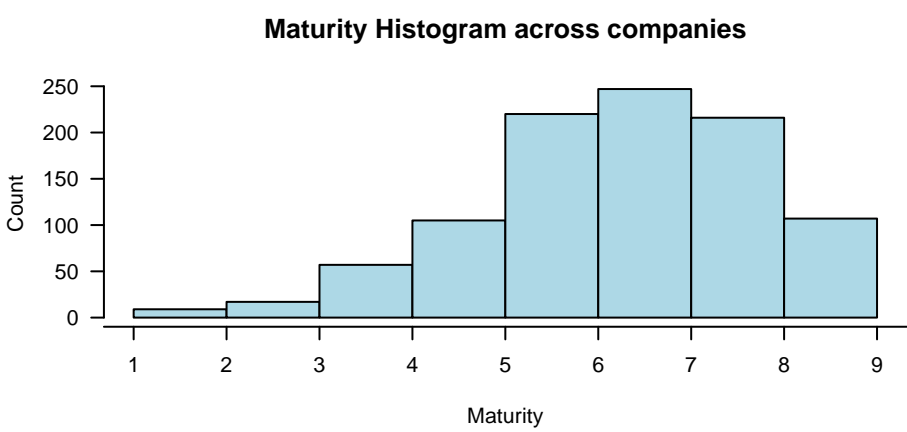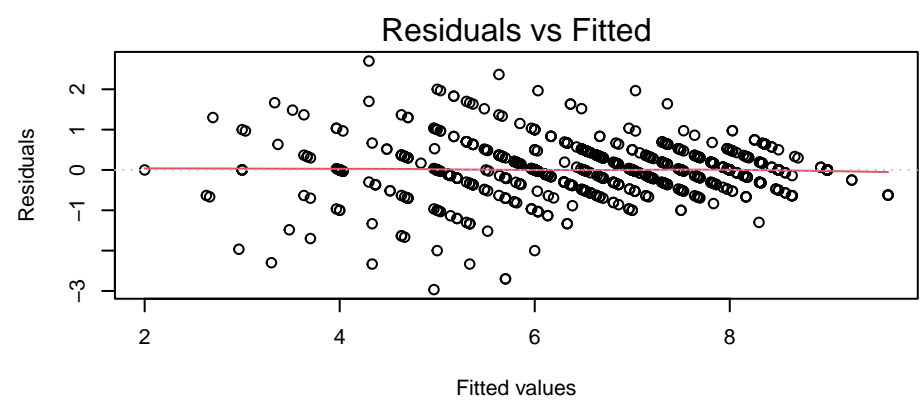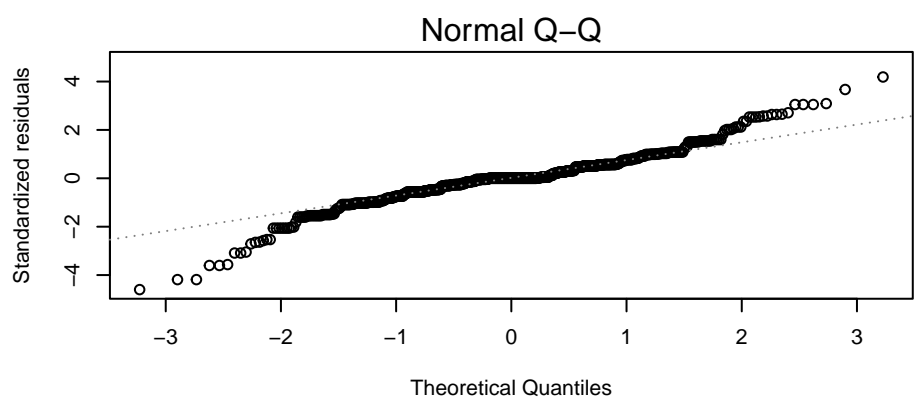

Supplement: jkae164_Supplementary_Data [file jkae164_supplementary_data.zip › Supplemental_File_3_G3-2024-405051.pdf]
